# Asymmetric Synthesis of the C15–C32 Fragment of Alotamide and Determination of the Relative Stereochemistry

**DOI:** 10.3390/md16110414

**Published:** 2018-10-30

**Authors:** Hao-yun Shi, Yang Xie, Pei Hu, Zi-qiong Guo, Yi-hong Lu, Yu Gao, Cheng-gang Huang

**Affiliations:** 1 Shanghai Institute of Materia Medica, Chinese Academy of Sciences, 501 Haike Road, Shanghai 201203, China; baiyinyun@hotmail.com (H.-y.S.), sunshine_xie@163.com (Y.X.); hupeishtcm@126.com (P.H.); 18673675362@163.com (Z.-q.G.); xiong1743771101@163.com (Y.-h.L.); 2 School of Pharmacy, University of Chinese Academy of Sciences, No. 19A Yuquan Road, Beijing 100049, China

**Keywords:** alotamide, asymmetric synthesis, relative structural determination

## Abstract

Alotamide is a cyclic depsipetide isolated from a marine cyanobacterium and possesses a unique activation of calcium influx in murine cerebrocortical neurons (EC_50_ 4.18 µM). Due to its limited source, the three stereocenters (C19, C28, and C30) in its polyketide fragment remain undetermined. In this study, the first asymmetric synthesis of its polyketide fragment was achieved. Four relative possible diastereomers were constructed with a boron-mediated enantioselective aldol reaction and Julia–Kocienski olefination as the key steps. Comparison of ^13^C NMR spectra revealed the relative structure of fragment C15–C32 of alotamide.

## 1. Introduction

Recently, several active secondary metabolites have been isolated from marine cyanobacterium and some of these metabolites demonstrate excellent bioactivities such as cytotoxic, antimicrobial, and antiprotozoal properties [1,2]. For example, apratoxins display potent cytotoxicity against several cancer cells at the nanoscale level and have become the new lead compounds in anticancer drug discovery [3,4,5,6].

Alotamide was also isolated from the marine cyanobacterium *Lyngbya bouillonii* in 2009 [7]. It is a cyclic depsipeptide and structurally has two parts. The northern part is a tripetide that consists of *N*-Me-Val, Cys-derived thiazolene ring, and Pro and the southern part is a special unsaturated polyketide with three undetermined stereocenters (C19, C28, and C30). Functionally, alotamide is a unique calcium influx activator in murine cerebrocortical neurons (EC_50_ 4.18 µM). Given that calcium overload is involved in physiological processes and may lead to several nervous diseases such as AD and epilepsy, this compound has gained increasing attention as a new neurotoxin from the marine resource [8]. In view of the limited natural source, a concise synthetic strategy of alotamide should be developed. In this study, we described the first asymmetric synthesis of its polyketide fragment C15–C32 and obtained four possible diastereomers. The relative stereochemistry was assigned after the NMR comparison.

## 2. Results

**Figure 1:**
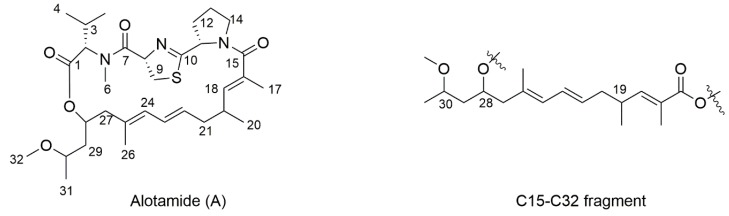
Structure of alotamide.

**Figure 2:**
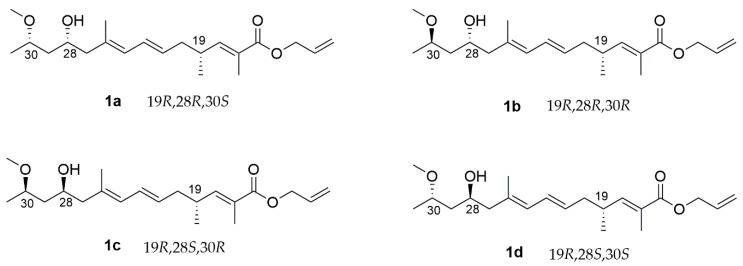
Structures of four diastereomers **1a**–**1d**.

The chemical structure of alotamide is shown in Figure 1. The southern polyketide with three undetermined stereocenters established eight possible isomers and only four needed to be evaluated for relative stereochemical determination. In this regard, we set C19 as *R* and listed four diastereomers, which are shown in Figure 2 (**1a**–**1d**).

**Scheme 1:**
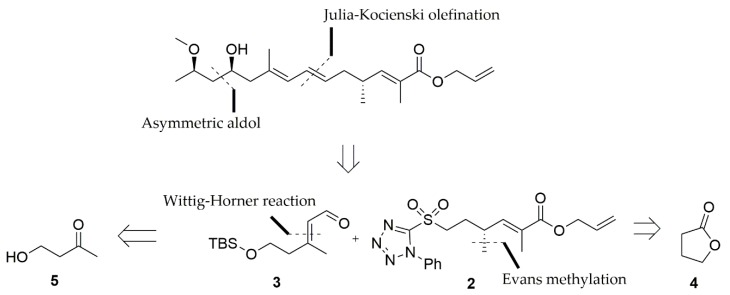
Retrosynthetic analysis.

According to the retrosynthetic analysis (Scheme 1), the dihydroxy unit would arise from an asymmetric aldol reaction and the Julia–Kocienski olefination would be applied to form the diene part. The polyketide fragment would be separated into two subunits, **2** and **3**, which both could be prepared from commercial compounds.

**Scheme 2:**
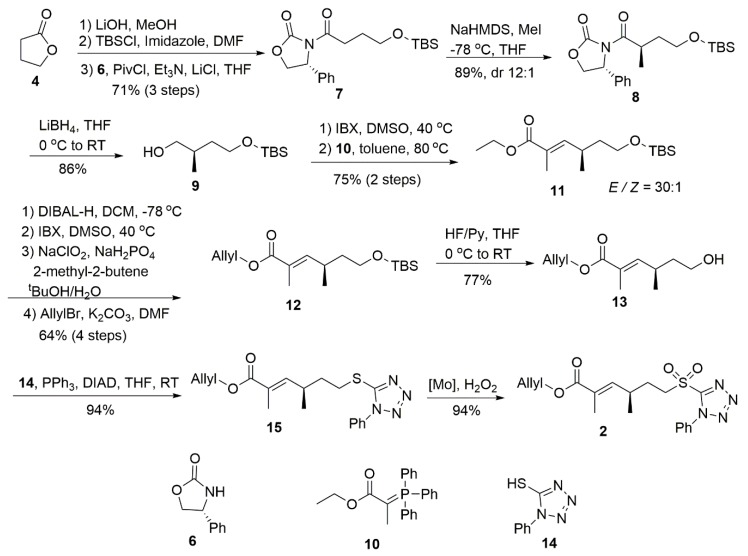
Synthesis of subunit **2**.

Compound **2** was prepared from commercial lactone **4** and treated with LiOH to open the lactone ring (Scheme 2) [9]. The resulting compound was subjected to TBS protection and condensation with Evan’s protocol **6** [10] in sequence to obtain **7** in 71% yield over three steps. Compound **7** was treated with NaHMDS/MeI at −78 °C to obtain the desired *R*-methyl **8** in 89% yield (dr = 12:1). After the reduction of **8** by LiBH_4_ [11], alcohol **9** was oxidized into the corresponding aldehyde, which was immediately refluxed with the Wittig reagent **10** to generate olefin **11**. In this reaction, the *E/Z* selectivity reached 30:1 and the little *Z* isomer was separated in the following DIBAL-H reduction. The sequential oxidation with IBX and Pinnick reaction generated a free acid and the allyl protection was conducted to acquire **12** in 64% yield for the above four steps. After the deprotection of the TBS group, the Mitsunobu reaction was applied to convert the compound **13** into tetrazole **15** in 94% yield. Then the SPT part was oxidized by H_2_O_2_ (20 eq) in the presence of (NH_4_)_6_Mo_7_O_24_ (0.2 eq) to obtain the desired **2** without the *Z* isomer [12]. 

**Scheme 3:**
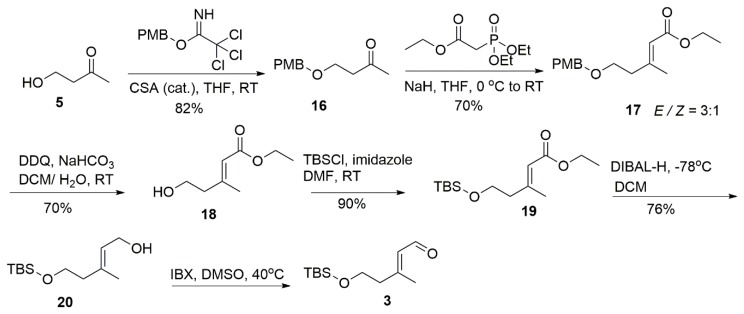
Synthesis of subunit **3**.

The preparation of subunit **3** was initiated with 4-hydroxy-2-butanone (Scheme 3). The Wittig–Horner reaction was conducted after PMB protection to smoothly obtain the desired product **17** (70% yield, *E/Z* = 3:1) while a complex mixture appeared when TBS protected alcohol was applied in this HWE reaction [13,14]. After the removal of the PMB group followed by the TBS protection, compound **19** was obtained and then converted into the corresponding aldehyde **3** by sequential steps of DIBAL-H reduction and IBX oxidation prior to the Julia–Kocienski olefination.

The different bases were tested through olefination (Table 1). When 1 eq KHMDS (entry 1) was applied, the yield was 50% and numerous reactants **2** and **3** remained. The product diene was an *E/Z* mixture with *E/Z* selectivity of up to 15:1. When the amount of KHMDS was increased to 1.5 eq, the yield improved and the two reactants remained in small quantities. When 2 eq KHMDS was used, the two reactants were completely consumed and the yield reached 86% while the high selectivity (15:1) was maintained. Other bases such as LiHMDS were also tried, but the resulting *E/Z* selectivity was low. We also exchanged the aldehyde and SO_2_PT functional groups and subjected them to olefination (entry 5). The yield of the *Z* isomer greatly increased and the selectivity was 1.5:1 [15,16].

**Scheme 4:**
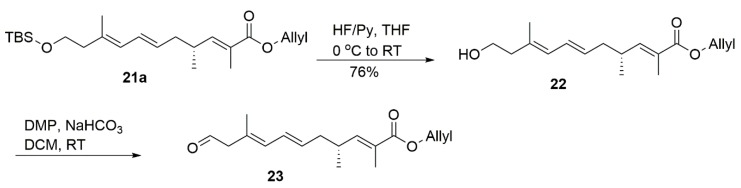
Synthesis of compound **23**.

Having completed the construction of diene **21a**, we started to prepare the dihydroxy unit (Scheme 4). To our delight, under the condition of the HF/Py complex, we fairly achieved the alcohol **22** and oxidized it to corresponding aldehyde **23** by DMP prior to the aldol reaction (deprotection of PMB group in this step led to a complex mixture).

IPCBCl-controlled aldol reaction [17,18] was selected to install the C28 stereo-center (Table 2). The application of (−)-IPCBCl at −20 °C successfully generated **24b** with the C28 (*S*) configuration in 59% yield (dr = 99:1). The chiral reactant was changed into (+)-IPCBCl, which afforded the C28 (*R*) product as expected and maintained the high diastereoselectivity (dr = 98:2). LiHMDS also proceeded and a 1:1 mixture was obtained in this aldol reaction.

**Scheme 5:**
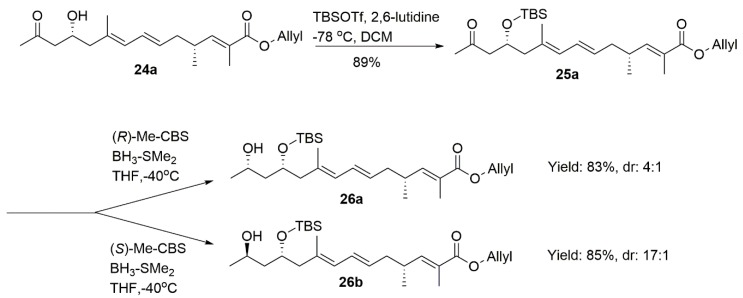

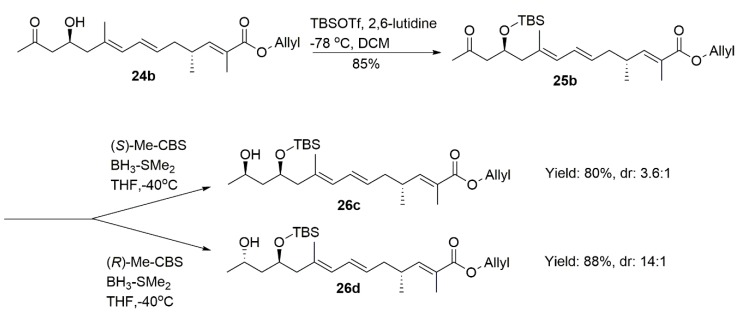
Synthesis of **26a**–**26d**.

After TBS protection followed by the reduction of the combination of BH_3_-DMS and CBS catalysts, we obtained **26a** (dr = 4:1, determined by ^1^H NMR) and **26b** (dr = 17:1, determined by ^1^H NMR), respectively. Compounds **26c** (dr = 3.6:1, determined by ^1^H NMR) and **26d** (dr = 14:1, determined by ^1^H NMR) were achieved from **25b** with the similar dr value (Scheme 5).

**Scheme 6:**
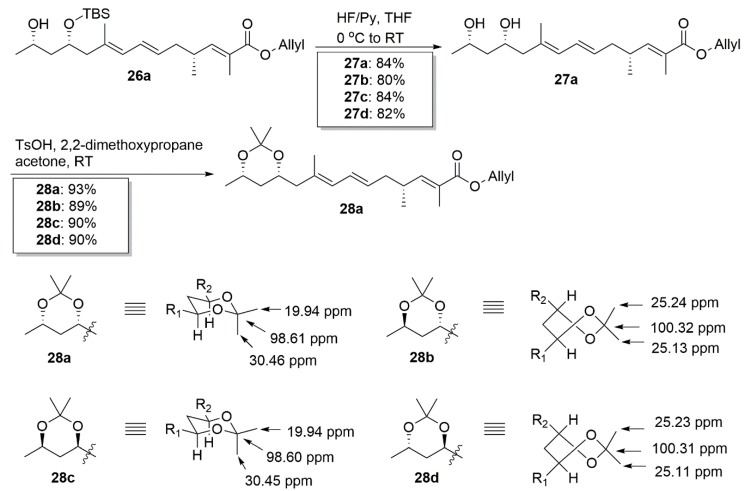
Stereo-chemical assignments of **28a**–**28d**.

The stereochemistries of the 1,3-diol part in **26a**–**26d** were confirmed by their ^13^C NMR chemical shifts of the corresponding acetonides **28a**–**28d** (Scheme 6). *Syn*-diol acetonide preferred a chair conformation and two ketal methyl groups were significantly different (e.g., 19.94 and 30.46 ppm for **28a**). Meanwhile *anti*-diol acetonide preferred a twist-boat conformation and two similar methyl groups existed (e.g., 25.24 and 25.13 ppm for **28b**) [19,20,21].

**Scheme 7:**
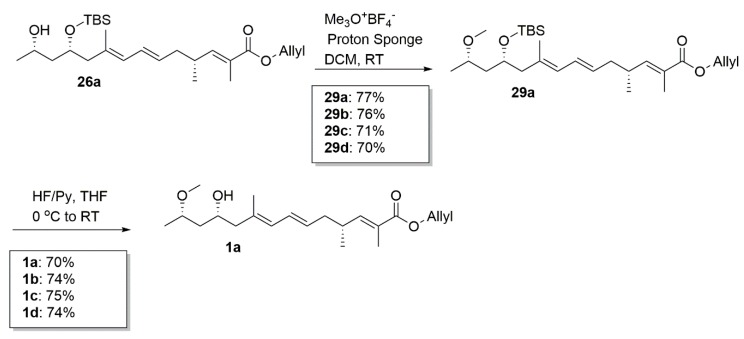
Synthesis of **1a**–**1d**.

After the sequential methylation and removal of the TBS group from **26a**–**26d**, we successfully furnished the four desired analogues **1a**–**1d** (Scheme 7). The total yield was 2.5% for **1a** from the lactone and 2.7% for **1b**, 2.5% for **1c**, and 2.6% for **1d**.

## 3. Discussion

**Figure 3:**
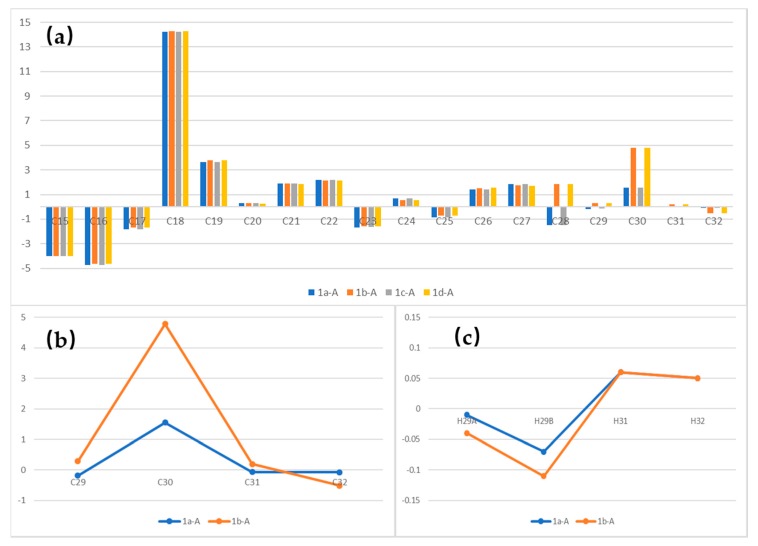
(**a**) Δδ ^13^C (ppm) between alotamide and **1a**–**1d**, (**b**) Δδ ^13^C (ppm) between alotamide and **1a** and **1b** from C29–C32, (**c**) Δδ ^1^H (ppm) between alotamide and **1a** and **1b** in dihydroxy unit.

A careful comparison between four isomers and alotamide was conducted. The differences in ^13^C NMR chemical shifts are shown in Figure 3a. From C15 to C28, several significant variations (Δδ > 1 ppm) existed between all four isomers and alotamide possibly because of the difference between the “straight-chain” mode in our analogues and the “ring” mode in the original structure. From C28 to C32, the dihydroxy unit is a linear chain both in our analogues and natural compound. Thus, comparing the data in this portion is suitable for relative stereochemistry determination. 

At the same time, several obvious variations in ^13^C spectra were observed between 1,3-*syn* isomers (**1a** and **1c**) and 1,3-*anti* isomers (**1b** and **1d**) from C29 to C32 (Figure 3a). Therefore, we chose **1a** and **1b** to represent the *syn*-analogues and *anti*-analogues to distinguish *syn*-configurations and *anti*-configurations (Figure 3b,c).

A significant variation at C30 position of **1b** (Δδ > 4 ppm) and a closer correlation of **1a** at C29, C31 and C32 in the ^13^C spectra revealed the *syn* configuration in the C28–C30 unit. In addition, smaller variations with alotamide in ^1^H spectra were noticed for analogue **1a** at protons H29A and H29B (differences between alotamide and **1a** and **1b** at proton H30 were not plotted in Figure 3c because the differences were all greater than 0.4 ppm and insignificant). This finding confirmed the 1,3-*syn* structure in the dihydroxy unit.

Two 1,3-*syn* isomers **1a** and **1c** were also compared. Their ^1^H spectra were the same and the differences in ^13^C NMR chemical shifts are listed in Table 3. A closer correlation of **1c** was observed especially at the sites C20, C29, and C31. Thus, it appeared that **1c** (19*R*, 28*S*, 30*R*) most closely fit the original natural alotamide. The total synthesis of alotamide with fragment **1c** and another (19*S*, 28*R*, 30*S*) enantiomer is in progress.

## 4. Materials and Methods

All anaerobic and moisture-sensitive manipulations were carried out with standard Schlenk techniques under argon. Solvents were dried and distilled by standard procedures. ^1^H- NMR and ^13^C-NMR spectra were recorded in CDCl_3_ on a Bruker Ascend-400 400 MHz or Bruker Ascend-500 500 MHz at room temperature. Chemical shifts (δ) are reported in ppm and are referenced to chloroform (δ 7.26 ppm for 1H, δ 77.16 ppm for ^13^C). Data for NMR spectra are reported as follows: s = singlet, d = doublet, t = triplet, q = quartet, quint. = quintet, sext. = sextet, m = multiplet, br = broad signal, J = coupling constant in Hz. HRMS were recorded on an Agilent 6530-Q-TOF mass spectrometer equipped with an Agilent 1260- HPLC. Optical rotations were measured on a PerkinElmer 241 MC polarimeter.

### 4.1. (R)-3-(4-((Tert-Butyldimethylsilyl)Oxy)Butanoyl)-4-Phenyloxazolidin-2-One (**7**)

To a solution of the lactone (5 g, 58 mmol) in MeOH (50 mL) at room temperature was added LiOH (2.44 g, 58 mmol) and stirred overnight. The solvent was removed from the reaction and the residue was dissolved in dimethylformamide (50 mL) at 0 °C. Imidazole (8 g, 116 mmol) was added to this solution, which was followed by TBSCl (8.7 g, 58 mmol) in two portions over 15 min. The reaction mixture was warmed to room temperature overnight with stirring and then diluted with 1 M HCl and extracted with EtOAc (3 × 100 mL). The combined organic layers were washed with brine and dried over Na_2_SO_4_. The organic layer was removed under vacuum and the crude acid was used without further purification.

To a stirred solution of the acid in dry THF (200 mL) at 0 °C under argon, Et_3_N (20 mL, 145 mmol) and PivCl (7.1 mL, 58 mmol) were added sequentially. After 1 h stirring at 0 °C, LiCl (0.62 g, 14.5 mmol), followed by oxazolidinone **6** (9.4 g, 58 mmol), were added. The reaction was continued for 1 h at 0 °C and another 2 h at room temperature prior to quenching with a saturated NH_4_Cl solution (50 mL) and extracted with DCM (2 × 200 mL). The combined organic layers were washed with brine, dried over Na_2_SO_4_, and concentrated in vacuum. Purification by column chromatography (PE/EA = 9:1) afforded compound **7** (15 g, 71% for three steps) as a colorless oil.

**^1^H NMR** (400 MHz, CDCl_3_) δ 7.47–7.21 (m, 5H), 5.43 (dd, *J* = 8.7, 3.6 Hz, 1H), 4.69 (td, *J* = 8.8, 1.0 Hz, 1H), 4.28 (ddd, *J* = 8.9, 3.6, 1.3 Hz, 1H), 3.64 (t, *J* = 6.3 Hz, 2H), 3.03 (qt, *J* = 17.7, 7.4 Hz, 2H), 1.92–1.77 (m, 2H), 0.90 (s, 9H), 0.04 (d, *J* = 1.3 Hz, 6H). **^13^C NMR** (101 MHz, CDCl_3_) δ 172.62, 153.76, 139.27, 129.18, 128.68, 125.93, 70.00, 62.25, 61.91, 57.56, 32.08, 27.09, 25.96, 18.32, −5.34, −5.62. ${\left[\alpha \right]}_{D}^{20}$ = −42.63, (*c* 3.38, CHCl_3_). HRMS (ESI+): calcd. for C_19_H_29_NO_4_S_i_ [M + H]^+^, 364.1939; found 364.1940.

### 4.2. (R)-3-((R)-4-((Tert-Butyldimethylsilyl)oxy)-2-Methylbutanoyl)-4-Phenyloxazolidin-2-one (**8**)

To a stirred solution of compound **7** (15 g, 41.2 mmol) in dry THF (100 mL) at −78 °C under argon, NaHMDS (2.0 M solution in THF, 24.7 mL, 49.4 mmol) was added dropwise. After 1 h, MeI (7.7 mL, 123.5 mmol) was added and the mixture was stirred overnight at the same temperature. The reaction mixture was quenched with a saturated NH_4_Cl solution (100 mL) and warmed up to room temperature before being extracted with DCM (2 × 100 mL). The combined organic extracts were washed with water and brine, dried over Na_2_SO_4_, and concentrated in vacuum. Purification by column chromatography (PE/EA = 13:1) gave pure **8** (13.8 g, 89%) as a white solid.

**^1^H NMR** (400 MHz, CDCl_3_) δ 7.45–7.23 (m, 5H), 5.44 (dd, *J* = 8.7, 3.7 Hz, 1H), 4.67 (t, *J* = 8.8 Hz, 1H), 4.25 (dd, *J* = 8.9, 3.7 Hz, 1H), 4.02–3.85 (m, 1H), 3.73–3.55 (m, 2H), 2.00 (td, *J* = 13.9, 6.4 Hz, 1H), 1.59 (dq, *J* = 12.2, 6.1 Hz, 1H), 1.15 (d, *J* = 7.0 Hz, 3H), 0.91 (s, 9H), 0.06 (s, 6H). **^13^C NMR** (101 MHz, CDCl_3_) δ 176.36, 153.35, 139.47, 129.28, 128.70, 125.75, 69.80, 61.02, 57.74, 35.72, 34.71, 26.00, 18.39, 17.83, −5.33. ${\left[\alpha \right]}_{D}^{20}$ = −79.14, (*c* 2.34, CHCl_3_). HRMS (ESI+): calcd. for C_20_H_31_NO_4_S_i_ [M + H]^+^, 378.2095, found 378.2095.

### 4.3. (R)-4-((Tert-Butyldimethylsilyl)Oxy)-2-Methylbutan-1-Ol (**9**)

To an ice-cold solution of compound **8** (13.8 g, 36.6 mmol) in THF (100 mL) moist with a catalytic amount of water, LiBH_4_ (1.2 g, 54.9 mmol) was added portion wise under argon. After 12 h of stirring at room temperature, the reaction was quenched cautiously with a saturated NH_4_Cl solution (50 mL) and then distilled under a reduced pressure followed by extraction with DCM. The combined organic solution was dried over Na_2_SO_4_ and concentrated in vacuum. Purification by column chromatography (PE/EA = 9:1) provided pure compound **9** (6.86 g, 86%) as a colorless oil [11].

**^1^H NMR** (400 MHz, CDCl_3_) δ 3.81–3.69 (m, 1H), 3.68–3.60 (m, 1H), 3.46 (s, 1H), 3.43–3.33 (m, 1H), 3.19 (s, 1H), 1.86–1.70 (m, 1H), 1.59–1.46 (m, 2H), 0.89 (m, 12H), 0.05 (s, 6H). **^13^C NMR** (101 MHz, CDCl_3_) δ 68.21, 61.82, 37.53, 34.46, 25.98, 18.34, 17.46, −5.33, −5.36. ${\left[\alpha \right]}_{D}^{20}$ = −10.98, (*c* 1.82, CHCl_3_). HRMS (ESI+): calcd. for C_11_H_26_O_2_S_i_ [M + H]^+^, 219.1775, found 219.1771.

### 4.4. Ethyl (R,E)-6-((Tert-Butyldimethylsilyl)Oxy)-2,4-Dimethylhex-2-Enoate (**11**)

To a stirred solution of **9** (6.86 g, 31.5 mmol) in DMSO (50 mL) IBX (10.6 g, 37.8 mmol) was added. After 1 h stirring at 40 °C, the reaction was quenched with water (50 mL) and extracted with ether (2 × 100 mL). The combined organic layers were washed with brine, dried over Na_2_SO_4_, and removed under vacuum. The residue was refluxed with 10 (22 g, 63 mmol) in toluene (100 mL) at 80 °C for 3 h and the solvent was removed under vacuum. Purification by column chromatography (PE/EA = 40:1) provided pure compound **11** (7 g, 75%) as a colorless oil [22].

**^1^H NMR** (400 MHz, CDCl_3_) δ 6.53 (dd, *J* = 10.1, 1.4 Hz, 1H), 4.17 (q, *J* = 7.1 Hz, 2H), 3.70–3.41 (m, 2H), 2.80–2.45 (m, 1H), 1.83 (d, *J* = 1.4 Hz, 3H), 1.66–1.44 (m, 2H), 1.28 (t, *J* = 7.1 Hz, 3H), 1.00 (d, *J* = 6.7 Hz, 3H), 0.87 (s, 9H), 0.01 (d, *J* = 2.1 Hz, 6H). **^13^C NMR** (101 MHz, CDCl_3_) δ 168.57, 147.56, 126.83, 61.02, 60.52, 39.83, 29.78, 26.04, 20.06, 18.37, 14.42, 12.59, −5.23, −5.25. ${\left[\alpha \right]}_{D}^{20}$ = −2.167, (*c* 0.1, CHCl_3_). HRMS (ESI+): calcd. for C_16_H_32_O_3_S_i_ [M + H]^+^, 301.2193, found 301.2194.

### 4.5. Allyl (R,E)-6-((Tert-Butyldimethylsilyl)oxy)-2,4-Dimethylhex-2-Enoate (**12**)

To a stirred solution of compound **11** (7 g, 23.6 mmol) in dry DCM (60 mL) at −78 °C under argon, DIBAL-H (1.5 M solution in toluene, 18.0 mL, 27.0 mmol) was added dropwise. After 1 h, the reaction mixture was quenched with aqueous sodium-potassium tartrate solution (20 mL) and warmed up to room temperature before being extracted with DCM (2 × 100 mL). The combined organic extracts were washed with water and brine, dried over Na_2_SO_4_, and concentrated in vacuum. Purification by column chromatography (PE/EA = 8:1) gave alcohol (4.8 g, 79%) as a colorless oil.

To a stirred solution of above alcohol in DMSO (50 mL), IBX (6.2 g, 22 mmol) was added. After 1 h stirring at 40 °C, the reaction was quenched with water (50 mL) and extracted with ether (2 × 100 mL). The combined organic layers were washed with brine, dried over Na_2_SO_4_, and removed under vacuum. The crude anhydride was used without further purification. 

To the solution of above crude aldehyde in ^t^BuOH (40 mL), NaH_2_PO_4_ (11 g, 93 mmol) in H_2_O (40 mL), 2-methyl-2-butene (19 mL, 186 mmol), and NaClO_2_ (3.1 g, 28 mmol, >79.0% purity) were added. After stirring for 2 h at room temperature, the mixture was extracted with EA (50 mL × 3) and the combined organic layers were washed with brine, dried over Na_2_SO_4_, filtrated, and concentrated. The crude acid was taken into the next step without future purification.

To a stirred solution of above acid in dry DMF (50 mL) allylBr (3.2 g, 37.2 mmol) and K_2_CO_3_ (5.1 g, 37.2 mmol) were added separately. After being stirred for 12 h, the mixture was quenched with a saturated aqueous solution of NH_4_Cl and extracted with EA (100 mL × 3). The combined organic layers were washed with brine, dried over Na_2_SO_4_, filtrated, and concentrated. The residue was purified by column chromatography on silica gel (PE/EA = 40:1) to give **12** (4.7 g, 81%) for three steps as a colorless oil.

**^1^H NMR** (400 MHz, CDCl_3_) δ 6.58 (dd, *J* = 10.1, 1.4 Hz, 1H), 5.96 (ddt, *J* = 17.1, 10.5, 5.6 Hz, 1H), 5.32 (ddd, *J* = 17.2, 3.0, 1.5 Hz, 1H), 5.23 (dd, *J* = 10.4, 1.3 Hz, 1H), 4.64 (dt, *J* = 5.6, 1.4 Hz, 2H), 3.66–3.42 (m, 2H), 2.82–2.58 (m, 1H), 1.86 (d, *J* = 1.4 Hz, 3H), 1.68–1.55 (m, 2H), 1.01 (d, *J* = 6.7 Hz, 3H), 0.88 (s, 10H), 0.02 (d, *J* = 2.2 Hz, 6H). **^13^C NMR** (101 MHz, CDCl_3_) δ 168.16, 148.14, 132.76, 126.60, 117.84, 65.27, 61.00, 39.80, 29.82, 26.04, 20.02, 18.38, 12.62, −5.22. ${\left[\alpha \right]}_{D}^{20}$= −53.365, (*c* 0.52, CHCl_3_). HRMS (ESI+): calcd. for C_17_H_32_O_3_S_i_ [M + H]^+^, 313.2193, found 313.2186.

### 4.6. Allyl (R,E)-6-Hydroxy-2,4-Dimethylhex-2-Enoate (**13**)

To a stirred solution of **12** (4.7 g, 15 mmol) in dry THF (10 mL), the HF/Py complex (2 mL) was added at 0 °C. After being stirred for 1 h, the mixture was quenched with a saturated aqueous solution of NaHCO_3_ and extracted with DCM (20 mL × 3). The combined organic layers were washed with 1 M HCl brine, dried over Na_2_SO_4_, filtrated, and concentrated. The residue was purified by column chromatography on silica gel (PE/EA = 6:1) to give **13** (2.3 g, 77%) as a colorless oil.

**^1^H NMR** (400 MHz, CDCl_3_) δ 6.59 (dd, *J* = 10.1, 1.4 Hz, 1H), 5.97 (ddt, *J* = 17.1, 10.6, 5.6 Hz, 1H), 5.34 (dq, *J* = 17.2, 1.5 Hz, 1H), 5.24 (ddd, *J* = 10.5, 2.6, 1.3 Hz, 1H), 4.65 (dt, *J* = 5.6, 1.4 Hz, 2H), 3.75–3.44 (m, 2H), 2.78–2.63 (m, 1H), 1.88 (d, *J* = 1.4 Hz, 3H), 1.75–1.62 (m, 1H), 1.62–1.50 (m, 2H), 1.05 (d, *J* = 6.7 Hz, 3H). **^13^C NMR** (101 MHz, CDCl_3_) δ 168.07, 147.61, 132.63, 126.88, 117.97, 65.34, 60.94, 39.55, 29.97, 20.09, 12.62. ${\left[\alpha \right]}_{D}^{20}$= −55.72, (*c* 0.79, CHCl_3_). HRMS (ESI+): calcd. for C_11_H_18_O_3_ [M + H]^+^, 199.1329, found 199.1328.

### 4.7. Allyl (R,E)-2,4-Dimethyl-6-((1-Phenyl-1H-Tetrazol-5-yl)Thio)Hex-2-Enoate (**15**)

To a stirred solution of **13** (2.3 g, 11.6 mmol) in anhydrous dry THF (50 mL) at 0 °C under argon, PPh_3_ (6.1 g, 23.2 mmol), **14** (2 g, 11.6 mmol), and DIAD (4.6 mL, 23.2 mmol) were added sequentially. The reaction was continued further for 1 h at room temperature prior to quenching with the saturated NH_4_Cl solution (50 mL) and extracted with DCM (2 × 100 mL). The combined organic layers were washed with brine, dried over Na_2_SO_4_, and concentrated in vacuum. Purification by column chromatography (PE/EA = 15:1) afforded compound **15** (3.9 g, 94%) as a colorless oil.

**^1^H NMR** (400 MHz, CDCl_3_) δ 7.67–7.46 (m, 5H), 6.55 (dd, *J* = 10.1, 1.4 Hz, 1H), 5.94 (ddt, *J* = 17.1, 10.5, 5.6 Hz, 1H), 5.42–5.29 (m, 1H), 5.22 (dd, *J* = 10.4, 1.3 Hz, 1H), 4.62 (dt, *J* = 5.6, 1.4 Hz, 2H), 3.41–3.13 (m, 2H), 2.77–2.60 (m, 1H), 2.06–1.90 (m, 1H), 1.89–1.74 (m, 4H), 1.05 (d, *J* = 6.7 Hz, 3H). **^13^C NMR** (101 MHz, CDCl_3_) δ 167.69, 154.18, 146.10, 133.74, 132.49, 130.22, 129.89, 127.82, 123.90, 118.07, 65.40, 35.95, 32.63, 31.37, 19.89, 12.76. ${\left[\alpha \right]}_{D}^{20}$ = −58.87, (*c* 1.47, CHCl_3_). HRMS (ESI+): calcd. for C_18_H_22_N_4_O_2_S [M + H]^+^, 359.1536, found 359.1532.

### 4.8. Allyl (R,E)-2,4-Dimethyl-6-((1-Phenyl-1H-Tetrazol-5-yl)sulfonyl)Hex-2-Enoate (**2**)

To a stirred solution of **15** (3.9 g, 10.1 mmol) in ethanol (100 mL) at 0 °C, (NH_4_)_6_Mo_7_O_24_ (2.9 g, 2.2 mmol) and H_2_O_2_ (20 mL) were added sequentially. The reaction was continued further for 12 h at room temperature prior to quenching with saturated NH_4_Cl solution (50 mL) and extracted with DCM (2 × 100 mL). The combined organic layers were washed with brine, dried over Na_2_SO_4_, and concentrated in vacuum. Purification by column chromatography (PE/EA = 8:1) afforded compound **2** (3.6 g, 94%) as a white solid.

**^1^H NMR** (400 MHz, CDCl_3_) δ 7.76–7.51 (m, 5H), 6.53 (dd, *J* = 10.1, 1.4 Hz, 1H), 5.97 (ddt, *J* = 17.1, 10.5, 5.6 Hz, 1H), 5.48–5.32 (m, 1H), 5.25 (dd, *J* = 10.4, 1.3 Hz, 1H), 4.65 (dt, *J* = 5.6, 1.3 Hz, 2H), 3.77–3.58 (m, 2H), 2.95–2.62 (m, 1H), 2.24–2.04 (m, 1H), 2.01–1.92 (m, 1H), 1.88 (d, *J* = 1.4 Hz, 3H), 1.11 (d, *J* = 6.7 Hz, 3H). **^13^C NMR** (101 MHz, CDCl_3_) δ 167.47, 153.49, 144.44, 133.12, 132.42, 131.64, 129.88, 128.93, 125.17, 118.32, 65.61, 54.40, 32.31, 28.74, 19.98, 12.86. ${\left[\alpha \right]}_{D}^{20}$ = −13.87, (*c* 0.79, CHCl_3_). HRMS (ESI+): calcd. for C_18_H_22_N_4_O_2_S_2_ [M + H]^+^, 391.1435, found 391.1435.

### 4.9. 4-((4-Methoxybenzyl)Oxy)Butan-2-One (**16**)

To a stirred solution of 4-hydroxy-2-butanone (1.1 mL ,12.8 mmol) in dry THF (20 mL) at 0 °C, 4-methoxybenzyl 2,2,2-trichloroacetimidate (2 mL, 10.7 mmol) and triphenylcarbenium tetrafluoroborate (cat.) were added sequentially. The reaction was continued further for 1 h at room temperature prior to quenching with the saturated NH_4_Cl solution (50 mL) and extracted with DCM (2 × 100 mL). The combined organic layers were washed with brine, dried over Na_2_SO_4_, and concentrated in vacuum. Purification by column chromatography (PE/EA = 6:1) afforded compound **16** (1.8 g, 82%) as a colorless oil [14].

**^1^H NMR** (400 MHz, CDCl_3_) δ 7.33–7.18 (m, 2H), 6.94–6.81 (m, 2H), 4.45 (s, 2H), 3.81 (s, 3H), 3.72 (t, *J* = 6.3 Hz, 2H), 2.71 (t, *J* = 6.3 Hz, 2H), 2.18 (s, 3H). **^13^C NMR** (101 MHz, CDCl_3_) δ 207.51, 159.27, 130.16, 129.42, 113.84, 72.91, 64.96, 55.31, 43.81, 30.48. HRMS (ESI+): calcd. for C_12_H_16_O_3_ [M + H]^+^, 209.1172, found 209.1170.

### 4.10. Ethyl (E)-5-((4-Methoxybenzyl)Oxy)-3-Methylpent-2-Enoate (**17**)

To a stirred solution of triethyl phosphonoacetate (3.5 mL, 17.5 mmol) in dry THF (50 mL) at 0 °C, NaH (0.7 g, 17.5 mmol) was added. After stirring at room temperature for 1 h, **16** (1.8 g, 8.8 mmol) in THF (5 mL) was added at 0 °C. The reaction was continued further for 12 h at room temperature prior to quenching with a saturated NH_4_Cl solution (50 mL) and extracted with DCM (2 × 100 mL). The combined organic layers were washed with brine, dried over Na_2_SO_4_, and concentrated in vacuum. Purification by column chromatography (PE/EA = 20:1) afforded compound **17** in the *E/Z* mixture (1.7 g, 70%) as a colorless oil.

**^1^H NMR** (400 MHz, CDCl_3_) δ 7.27 (d, *J* = 8.6 Hz, 2H), 6.99–6.72 (m, 2H), 5.82–5.63 (m, 1H), 4.47 (d, *J* = 2.8 Hz, 2H), 4.23–4.08 (m, 2H), 3.82 (d, *J* = 2.1 Hz, 3H), 3.70–3.51 (m, 2H), 2.98 (t, *J* = 6.6 Hz, 1H), 2.45 (t, *J* = 6.4 Hz, 2H), 2.19 (d, *J* = 1.2 Hz, 2H), 1.96 (d, *J* = 1.3 Hz, 1H), 1.29 (td, *J* = 7.1, 5.4 Hz, 3H). **^13^C NMR** (101 MHz, CDCl_3_) δ 166.73, 166.34, 159.33, 159.19, 157.81, 156.72, 130.70, 130.30, 129.38, 129.27, 117.46, 117.10, 113.90, 113.81, 72.76, 72.46, 68.74, 67.59, 59.60, 55.35, 40.89, 33.73, 26.16, 19.00, 14.42. HRMS (ESI+): calcd. for C_16_H_22_O_4_ [M + H]^+^, 279.1591; found 279.1589.

### 4.11. Ethyl (E)-5-Hydroxy-3-Methylpent-2-Enoate (**18**)

To a stirring solution of **17** (1.7 g, 6.1 mmol) in DCM (20 mL) and water (4 mL) DDQ (1.67 g, 7.3 mmol) was added at room temperature. After 1 h, the reaction mixture was quenched with saturated aqueous NaHCO_3_ (10 mL) and 1 M aqueous NaHSO_3_ (10 mL) and extracted with DCM (30 mL × 3). The combined organic layers were washed with brine, dried over Na_2_SO_4_, and concentrated. The crude product was purified by column chromatography (PE/EA = 3:1) to give the desired compound **18** as a colorless oil (672 mg, 70% yield) [13].

**^1^H NMR** (400 MHz, CDCl_3_) δ 5.71 (d, *J* = 1.2 Hz, 1H), 4.12 (q, *J* = 7.1 Hz, 2H), 3.76 (t, *J* = 6.4 Hz, 2H), 2.47–2.30 (m, 2H), 2.16 (d, *J* = 1.2 Hz, 3H), 1.92 (s, 1H), 1.25 (t, *J* = 7.1 Hz, 3H). **^13^C NMR** (101 MHz, CDCl_3_) δ 166.67, 156.12, 117.73, 60.22, 59.78, 43.82, 18.82, 14.37. HRMS (ESI+): calcd. for C_8_H_14_O_3_ [M + H]^+^, 159.1016, found 159.1014.

### 4.12. Ethyl (E)-5-((Tert-Butyldimethylsilyl)Oxy)-3-Methylpent-2-Enoate (**19**)

To a stirred solution of **18** (672 mg, 4.3 mmol) in anhydrous DMF (10 mL) at 0 °C, imidazole (7.7 g, 11.4 mmol) and TBSCl (1.1 g, 6.84 mmol) were added sequentially. The reaction was continued further for 12 h at room temperature prior to quenching with saturated NH_4_Cl solution (20 mL) and extracted with EA (2 × 20 mL). The combined organic layers were washed with brine, dried over Na_2_SO_4_, and concentrated in vacuum. Purification by column chromatography (PE/EA = 40:1) created compound **19** (1.05 g, 90%) as a colorless oil [13].

**^1^H NMR** (400 MHz, CDCl_3_) δ 5.66 (d, *J* = 1.1 Hz, 1H), 4.12 (q, *J* = 7.1 Hz, 2H), 2.32 (t, *J* = 6.6 Hz, 2H), 2.15 (d, *J* = 1.2 Hz, 3H), 1.24 (t, *J* = 7.1 Hz, 3H), 0.86 (s, 9H), 0.02 (s, 6H). **^13^C NMR** (101 MHz, CDCl_3_) δ 166.74, 156.92, 117.34, 61.41, 59.53, 44.12, 25.97, 19.21, 18.36, 14.42, −5.29. HRMS (ESI+): calcd. for C_14_H_28_O_3_Si [M + H]^+^, 273.1880, found 273.1880.

### 4.13. (E)-5-((Tert-Butyldimethylsilyl)Oxy)-3-Methylpent-2-En-1-Ol (**20**)

To a stirred solution of compound **19** (1.05 g, 3.87 mmol) in dry DCM (15 mL) at −78 °C under argon, DIBAL-H (1.5 M solution in toluene, 3.9 mL, 5.8 mmol) was added dropwise. After 1 h, the reaction mixture was quenched with the aqueous sodium-potassium tartrate solution (20 mL) and warmed up to room temperature before being extracted with DCM (2 × 20 mL). The combined organic extracts were washed with water and brine, dried over Na_2_SO_4_, and concentrated in vacuum. Purification by column chromatography (PE/EA = 7:1) gave **20** (680 mg, 76%) as a colorless oil [23].

**^1^H NMR** (400 MHz, CDCl_3_) δ 5.45 (td, *J* = 6.9, 1.2 Hz, 1H), 4.16 (d, *J* = 6.9 Hz, 2H), 3.71 (t, *J* = 7.0 Hz, 2H), 2.25 (t, *J* = 7.0 Hz, 2H), 1.71 (s, 3H), 1.53 (s, 1H), 0.90 (s, 9H), 0.06 (s, 6H). **^13^C NMR** (101 MHz, CDCl_3_) δ 136.95, 125.38, 62.20, 59.42, 42.91, 26.05, 18.45, 16.80, −5.17. HRMS (ESI+): calcd. for C_12_H_26_O_2_Si ([M + H]^+^, 231.1775, found 231.1775.

### 4.14. Allyl (R,2E,6E,8E)-11-((Tert-butyldimethylsilyl)Oxy)-2,4,9-Trimethylundeca-2,6,8-Trienoate (**21a**)

To a stirred solution of **20** (680 mg, 3.0 mmol) in DMSO (10 mL) IBX (990 mg, 3.5 mmol) was added. After 1 h of stirring at 40 °C, the reaction was quenched with water (10 mL) and extracted with ether (2 × 20 mL). The combined organic layers were washed with brine, dried over Na_2_SO_4_, and removed under vacuum. The crude aldehyde **3** was used without further purification.

To a stirred solution of crude **3** and compound **2** (1.3 g, 3.3 mmol) in dry THF (30 mL) at −78 °C under argon, KHMDS (1.0 M solution in THF, 27.0 mL, 27.0 mmol) was added dropwise. After 1 h, the reaction mixture was quenched with the saturated NH_4_Cl solution (20 mL) and warmed up to room temperature before being extracted with DCM (2 × 100 mL). The combined organic extracts were washed with water and brine, dried over Na_2_SO_4_, and concentrated in vacuum. Purification by column chromatography (PE/EA = 40:1) gave **21a** (1.0 g, 86%, 2 steps) as a colorless oil.

**^1^H NMR** (400 MHz, CDCl_3_) δ 6.61 (dd, *J* = 10.0, 1.4 Hz, 1H), 6.23 (dd, *J* = 15.0, 10.8 Hz, 1H), 5.97 (ddt, *J* = 17.1, 10.5, 5.6 Hz, 1H), 5.79 (d, *J* = 10.8 Hz, 1H), 5.48 (dt, *J* = 14.8, 7.3 Hz, 1H), 5.33 (ddd, *J* = 17.2, 3.1, 1.5 Hz, 1H), 5.23 (dd, *J* = 10.4, 1.3 Hz, 1H), 4.64 (d, *J* = 5.5 Hz, 2H), 3.68 (t, *J* = 7.0 Hz, 2H), 2.64–2.47 (m, 1H), 2.24 (t, *J* = 7.0 Hz, 2H), 2.14 (t, *J* = 7.1 Hz, 2H), 1.85 (d, *J* = 1.3 Hz, 3H), 1.74 (s, 3H), 1.02 (d, *J* = 6.7 Hz, 3H), 0.88 (s, 9H), 0.04 (s, 6H). **^13^C NMR** (101 MHz, CDCl_3_) δ 168.13, 147.84, 134.13, 132.75, 129.61, 128.53, 126.49, 126.40, 117.83, 65.27, 62.39, 43.29, 40.02, 33.96, 26.09, 19.68, 18.47, 17.20, 12.73, −5.14. ${\left[\alpha \right]}_{D}^{20}$ = −32.72, (*c* 0.38, CHCl_3_). HRMS (ESI+): calcd. for C_23_H_40_O_3_Si [M + H]^+^, 293.2819, found 293.2819.

### 4.15. Allyl (R,2E,6E,8E)-11-Hydroxy-2,4,9-Trimethylundeca-2,6,8-Trienoate (**22**)

To a stirred solution of **21a** (1.0 g, 2.6 mmol) in dry THF (5 mL), HF/Py complex (1 mL) was added at 0 °C. After being stirred for 1 h, the mixture was quenched with a saturated aqueous solution of NaHCO_3_ and extracted with DCM (20 mL × 3). The combined organic layers were washed with 1 M HCl, brine, dried over Na_2_SO_4_, filtrated, and concentrated. The residue was purified by column chromatography on silica gel (PE/EA = 6:1) to give **22** (550 mg, 76%) as a colorless oil.

**^1^H NMR** (400 MHz, CDCl_3_) δ 6.60 (dd, *J* = 10.0, 1.4 Hz, 1H), 6.24 (dd, *J* = 15.0, 10.8 Hz, 1H), 5.96 (ddt, *J* = 17.1, 10.5, 5.6 Hz, 1H), 5.85 (d, *J* = 10.8 Hz, 1H), 5.52 (dt, *J* = 14.8, 7.3 Hz, 1H), 5.32 (ddd, *J* = 17.2, 3.0, 1.5 Hz, 1H), 5.22 (dd, *J* = 10.4, 1.3 Hz, 1H), 4.63 (d, *J* = 5.6 Hz, 2H), 3.69 (t, *J* = 6.3 Hz, 2H), 2.65–2.51 (m, 1H), 2.29 (t, *J* = 6.3 Hz, 2H), 2.15 (t, *J* = 7.0 Hz, 2H), 1.84 (d, *J* = 1.4 Hz, 3H), 1.75 (s, 3H), 1.54 (s, 1H), 1.01 (d, *J* = 6.7 Hz, 3H). **^13^C NMR** (101 MHz, CDCl_3_) δ 168.09, 147.69, 132.93, 132.70, 130.49, 128.14, 127.38, 126.53, 117.82, 65.26, 60.49, 42.95, 39.97, 33.84, 19.67, 16.50, 12.72. ${\left[\alpha \right]}_{D}^{20}$= −38.23, (*c* 0.96, CHCl_3_). HRMS (ESI+): calcd. for C_17_H_26_O_3_ [M + H]^+^, 279.1955, found 279.1954.

### 4.16. Allyl (2E,4R,6E,8E,11R)-11-Hydroxy-2,4,9-Trimethyl-13-Oxotetradeca-2,6,8-Trienoate (**24a**)

To the above alcohol **22** (200 mg, 0.72 mmol) in dry DCM (5 mL, 0 °C), DMP (610 mg, 1.44 mmol) and NaHCO_3_ (240 mg, 2.9 mmol) was added sequentially. After being stirred for 30 min, the mixture was carefully quenched with a solution of saturated aqueous NaHCO_3_ and Na_2_S_2_O_3_. The resulting mixture was extracted with DCM (20 mL × 3) and the combined organic layers were washed with brine, dried over Na_2_SO_4_, and concentrated. The crude aldehyde **23** was used without future purification.

To a stirred solution of (+)-IPCBCl (1.8 M in heptane, 1.08 mmol, 0.6 mL) in dry ether (3 mL) at −0 °C under argon, Et_3_N (0.2 mL, 1.44 mmol) and acetone (80 uL, 1.08 mmol) were added sequentially. After 1 h stirring at −0 °C, aldehyde **23** in ether (2 mL) was added at −78 °C. The reaction was continued further for 1 h at −78 °C and another 12 h at −20 °C prior to quenching with a mixture of PH 7 buffer (1 mL), methanol (1 mL), and H_2_O_2_ (1 mL). The mixture was warmed up to room temperature before being extracted with ether (2 × 10 mL). The combined organic extracts were washed with water and brine, dried over Na_2_SO_4_, and concentrated in vacuum. Purification by column chromatography (PE/EA = 4:1) created **24a** (132 mg, 55%) as a colorless oil.

**^1^H NMR** (400 MHz, CDCl_3_) δ 6.60 (dd, *J* = 10.0, 1.2 Hz, 1H), 6.22 (dd, *J* = 15.0, 10.8 Hz, 1H), 5.96 (ddt, *J* = 17.1, 10.5, 5.6 Hz, 1H), 5.83 (d, *J* = 10.8 Hz, 1H), 5.52 (dt, *J* = 14.8, 7.3 Hz, 1H), 5.32 (dd, *J* = 17.2, 1.4 Hz, 1H), 5.23 (dd, *J* = 10.4, 1.1 Hz, 1H), 4.64 (d, *J* = 5.5 Hz, 2H), 4.20 (tt, *J* = 11.6, 5.9 Hz, 1H), 2.79 (s, 1H), 2.66–2.47 (m, 3H), 2.24 (dd, *J* = 13.4, 7.5 Hz, 1H), 2.19–2.07 (m, 6H), 1.84 (d, *J* = 1.2 Hz, 3H), 1.75 (s, 3H), 1.02 (d, *J* = 6.7 Hz, 3H).**^13^C NMR** (101 MHz, CDCl_3_) δ 209.56, 168.10, 147.68, 132.82, 132.72, 130.79, 128.10, 127.99, 126.58, 117.85, 65.91, 65.29, 49.62, 47.13, 40.02, 33.86, 30.98, 19.72, 16.89, 12.75. ${\left[\alpha \right]}_{D}^{20}$ = −33.92, (*c* 0.34, CHCl_3_). HRMS (ESI+): calcd. for C_20_H_30_O_4_ [M + H]^+^, 335.2217, found 335.2217.

### 4.17. Allyl (2E,4R,6E,8E,11S)-11-Hydroxy-2,4,9-Trimethyl-13-Oxotetradeca-2,6,8-Trienoate (**24b**)

To a stirred solution of (−)-IPCBCl (1.7 M in heptane, 1.08 mmol, 0.64 mL) in dry ether (3 mL) at 0 °C under argon, Et_3_N (0.2 mL, 1.44 mmol) and acetone (80 uL, 1.08 mmol) were added sequentially. After 1 h stirring at 0 °C, aldehyde **23** in ether (2 mL) was added at −78 °C. The reaction was continued further for 1 h at −78 °C and another 12 h at −20 °C prior to quenching with a mixture of PH 7 buffer (1 mL), methanol (1 mL), and H_2_O_2_ (1 mL). The mixture was warmed up to room temperature before being extracted with ether (2 × 10 mL). The combined organic extracts were washed with water and brine, dried over Na_2_SO_4_, and concentrated in vacuum. Purification by column chromatography (PE/EA = 4:1) provided **24b** (142 mg, 59%) as a colorless oil.

**^1^H NMR** (400 MHz, CDCl_3_) δ 6.60 (dd, *J* = 10.0, 1.2 Hz, 1H), 6.23 (dd, *J* = 15.0, 10.8 Hz, 1H), 5.96 (ddt, *J* = 17.1, 10.5, 5.6 Hz, 1H), 5.83 (d, *J* = 10.8 Hz, 1H), 5.53 (dt, *J* = 14.8, 7.3 Hz, 1H), 5.32 (dd, *J* = 17.2, 1.4 Hz, 1H), 5.23 (dd, *J* = 10.4, 1.1 Hz, 1H), 4.64 (d, *J* = 5.5 Hz, 2H), 4.29–4.12 (m, 1H), 2.66–2.51 (m, 3H), 2.23 (dd, *J* = 13.5, 7.6 Hz, 1H), 2.19–2.08 (m, 6H), 1.84 (d, *J* = 1.2 Hz, 3H), 1.75 (s, 3H), 1.02 (d, *J* = 6.7 Hz, 3H). **^13^C NMR** (101 MHz, CDCl_3_) δ 209.53, 168.10, 147.67, 132.83, 132.72, 130.76, 128.12, 127.98, 126.57, 117.86, 65.92, 65.29, 49.66, 47.11, 39.97, 33.86, 30.98, 19.69, 16.91, 12.74. ${\left[\alpha \right]}_{D}^{20}$ = −26.04, (*c* 0.37, CHCl_3_). HRMS (ESI+): calcd. for C_20_H_30_O_4_ [M + H]^+^, 335.2217, found 335.2216.

### 4.18. Allyl (2E,4R,6E,8E,11R)-11-((Tert-Butyldimethylsilyl)oxy)-2,4,9-Trimethyl-13-Oxotetradeca-2,6,8-Trienoate (**25a**)

To a stirred solution of compound **24a** (132 mg, 0.4 mmol) in dry DCM (3 mL) at −78 °C under argon, 2,6-lutidine (0.23 mL, 2 mmol) and TBSOTf (0.35 mL, 1.5 mmol) were added sequentially. After 1 h, the reaction mixture was quenched with aqueous NaHCO_3_ (5 mL) and warmed up to room temperature before being extracted with DCM (2 × 10 mL). The combined organic extracts were washed with 1 M HCl and brine, dried over Na_2_SO_4_, and concentrated in vacuum. Purification by column chromatography (PE/EA = 40:1) created **25a** (160 mg, 89%) as a colorless oil.

**^1^H NMR** (400 MHz, CDCl_3_) δ 6.60 (dd, *J* = 10.0, 1.3 Hz, 1H), 6.19 (dd, *J* = 15.0, 10.8 Hz, 1H), 5.96 (ddt, *J* = 17.1, 10.5, 5.6 Hz, 1H), 5.75 (d, *J* = 10.8 Hz, 1H), 5.48 (dt, *J* = 14.8, 7.3 Hz, 1H), 5.33 (dd, *J* = 17.2, 1.5 Hz, 1H), 5.23 (dd, *J* = 10.4, 1.2 Hz, 1H), 4.64 (d, *J* = 5.6 Hz, 2H), 4.38–4.20 (m, 1H), 2.67–2.41 (m, 3H), 2.29–2.20 (m, 1H), 2.19–2.00 (m, 6H), 1.84 (d, *J* = 1.1 Hz, 3H), 1.73 (s, 3H), 1.01 (d, *J* = 6.7 Hz, 3H), 0.85 (s, 9H), 0.03 (d, *J* = 8.1 Hz, 6H). **^13^C NMR** (101 MHz, CDCl_3_) δ 208.04, 168.10, 147.75, 133.24, 132.74, 130.28, 128.31, 128.07, 126.53, 117.86, 68.06, 65.29, 50.86, 48.64, 40.00, 33.93, 31.85, 25.96, 19.67, 18.12, 17.30, 12.74, −4.41, −4.71. ${\left[\alpha \right]}_{D}^{20}$ = −40.90, (*c* 0.26, CHCl_3_). HRMS (ESI+): calcd. for C_26_H_44_O_4_Si [M + H]^+^, 449.3082, found 449.3078.

### 4.19. Allyl (2E,4R,6E,8E,11S)-11-((Tert-Butyldimethylsilyl)oxy)-2,4,9-Trimethyl-13-Oxotetradeca-2,6,8-Trienoate (**25b**)

The procedure was identical to **25a**. Compound **25b** (160 mg, 85%) was obtained as a colorless oil.

**^1^H NMR** (400 MHz, CDCl_3_) δ 6.60 (dd, *J* = 10.0, 1.3 Hz, 1H), 6.19 (dd, *J* = 15.0, 10.8 Hz, 1H), 5.96 (ddt, *J* = 17.1, 10.5, 5.6 Hz, 1H), 5.74 (d, *J* = 10.8 Hz, 1H), 5.48 (dt, *J* = 14.8, 7.3 Hz, 1H), 5.32 (dd, *J* = 17.2, 1.5 Hz, 1H), 5.23 (dd, *J* = 10.4, 1.3 Hz, 1H), 4.64 (d, *J* = 5.5 Hz, 2H), 4.35–4.23 (m, 1H), 2.66–2.42 (m, 3H), 2.23 (dd, *J* = 13.2, 5.7 Hz, 1H), 2.19–2.05 (m, 6H), 1.84 (d, *J* = 1.2 Hz, 3H), 1.73 (s, 3H), 1.01 (d, *J* = 6.7 Hz, 3H), 0.84 (s, 9H), 0.02 (d, *J* = 7.1 Hz, 6H). **^13^C NMR** (101 MHz, CDCl_3_) δ 208.07, 168.09, 147.76, 133.25, 132.74, 130.27, 128.31, 128.04, 126.52, 117.85, 68.09, 65.28, 50.84, 48.60, 40.04, 33.92, 31.84, 25.95, 19.71, 18.11, 17.33, 12.73, −4.42, −4.74. ${\left[\alpha \right]}_{D}^{20}$ = −17.13, (*c* 0.51, CHCl_3_). HRMS (ESI+): calcd. for C_26_H_44_O_4_Si [M + H]^+^, 449.3082, found 449.3079.

### 4.20. Allyl (2E,4R,6E,8E,11R,13S)-11-((Tert-Butyldimethylsilyl)Oxy)-13-Hydroxy-2,4,9-Trimethyltetradeca-2,6,8-Trienoate (**26a**)

To a solution of (*R*)-Me-CBS (1 M in toluene, 0.05 mL, 0.05 mmol) in dry THF (5 mL) was slowly added BH_3_·DMS (12 uL, 0.13 mmol) at −40 °C. After being stirred for 30 min at the same temperature, a solution of **25a** (57 mg, 0.13 mmol) in THF (2 mL) was slowly added. After being stirred for 2 h at −40 °C, the mixture was diluted with MeOH. The resulting mixture was concentrated and extracted with DCM (30 mL × 3). The combined organic layers were washed with brine, dried, filtrated, and concentrated. The residue was purified by column chromatography on silica gel (PE/EA = 15:1) to give a mixture of **26a** and **26b** (4:1, 48 mg, 83%) as a colorless oil (pure major isomer **26a** can be obtained by repeating the purification on silica gel).

**^1^H NMR** (400 MHz, CDCl_3_) δ 6.60 (d, *J* = 9.9 Hz, 1H), 6.18 (dd, *J* = 14.7, 11.0 Hz, 1H), 5.96 (ddt, *J* = 17.1, 10.5, 5.6 Hz, 1H), 5.77 (d, *J* = 10.8 Hz, 1H), 5.49 (dt, *J* = 14.8, 7.3 Hz, 1H), 5.32 (dd, *J* = 17.2, 1.4 Hz, 1H), 5.23 (dd, *J* = 10.5, 1.0 Hz, 1H), 4.63 (d, *J* = 5.5 Hz, 2H), 4.27–4.06 (m, 2H), 2.66–2.47 (m, 1H), 2.35 (dd, *J* = 13.4, 6.3 Hz, 1H), 2.23 (dd, *J* = 13.3, 7.6 Hz, 1H), 2.16 (dd, *J* = 20.8, 13.7 Hz, 2H), 1.84 (s, 3H), 1.71 (s, 3H), 1.61 (ddd, *J* = 14.0, 10.0, 3.7 Hz, 1H), 1.48 (ddd, *J* = 14.5, 9.4, 3.7 Hz, 1H), 1.14 (d, *J* = 6.2 Hz, 3H), 0.99 (d, *J* = 11.9 Hz, 3H), 0.88 (s, 9H), 0.07 (d, *J* = 18.8 Hz, 6H). **^13^C NMR** (101 MHz, CDCl_3_) δ 168.12, 147.74, 133.01, 132.72, 130.26, 128.29, 127.95, 126.52, 117.86, 70.36, 65.29, 64.58, 46.98, 43.26, 40.03, 33.91, 25.95, 23.98, 19.68, 18.06, 17.15, 12.76, −4.51, −4.70. ${\left[\alpha \right]}_{D}^{20}$ = −17.67, (*c* 0.1, CHCl_3_). HRMS (ESI+): calcd. for C_26_H_46_O_4_Si [M + H]^+^, 451.3238, found 451.3241.

### 4.21. Allyl (2E,4R,6E,8E,11R,13R)-11-((Tert-Butyldimethylsilyl)Oxy)-13-Hydroxy-2,4,9-Trimethyltetradeca-2,6,8-Trienoate (**26b**)

To a solution of (*S*)-Me-CBS (1 M in toluene, 0.1 mL, 0.1 mmol) in dry THF (5 mL), BH_3_·DMS (24 uL, 0.25 mmol) was slowly added at −40 °C. After being stirred for 30 min at the same temperature, a solution of **25a** (110 mg, 0.24 mmol) in THF (2 mL) was slowly added. After being stirred for 2 h at −40 °C, the mixture was diluted with MeOH. The resulting mixture was concentrated and extracted with DCM (30 mL × 3). The combined organic layers were washed with brine, dried, filtrated, and concentrated. The residue was purified by column chromatography on silica gel (PE/EA = 15:1) to create **26b** (92 mg, 85%) as a colorless oil.

**^1^H NMR** (400 MHz, CDCl_3_) δ 6.60 (dd, *J* = 10.0, 1.3 Hz, 1H), 6.19 (dd, *J* = 15.0, 10.8 Hz, 1H), 6.04–5.92 (m, 1H), 5.77 (d, *J* = 10.8 Hz, 1H), 5.51 (dd, *J* = 14.9, 7.4 Hz, 1H), 5.33 (dd, *J* = 17.2, 1.5 Hz, 1H), 5.23 (dd, *J* = 10.4, 1.3 Hz, 1H), 4.64 (d, *J* = 5.5 Hz, 2H), 4.01 (dq, *J* = 13.0, 4.3 Hz, 1H), 3.96–3.83 (m, 1H), 3.18 (s, 1H), 2.66–2.50 (m, 1H), 2.32 (dd, *J* = 13.1, 4.6 Hz, 1H), 2.12 (dt, *J* = 13.2, 7.9 Hz, 3H), 1.84 (d, *J* = 1.1 Hz, 3H), 1.72 (s, 3H), 1.59–1.52 (m, 1H), 1.47–1.37 (m, 1H), 1.14 (d, *J* = 6.2 Hz, 3H), 1.02 (d, *J* = 6.7 Hz, 3H), 0.89 (s, 9H), 0.12 (d, *J* = 8.4 Hz, 6H). **^13^C NMR** (101 MHz, CDCl_3_) δ 168.12, 147.73, 132.79, 132.72, 130.34, 128.23, 127.93, 126.54, 117.86, 72.22, 67.43, 65.29, 49.26, 44.82, 40.01, 33.91, 25.95, 23.70, 19.70, 18.03, 17.28, 12.75, −3.77, −4.55. ${\left[\alpha \right]}_{D}^{20}$= −40.33, (*c* 0.35, CHCl_3_). HRMS (ESI+): calcd. for C_26_H_46_O_4_Si [M + H]^+^, 451.3238, found 451.3241.

### 4.22. Allyl (2E,4R,6E,8E,11S,13R)-11-((Tert-Butyldimethylsilyl)Oxy)-13-Hydroxy-2,4,9-Trimethyltetradeca-2,6,8-Trienoate (**26c**)

To a solution of (*S*)-Me-CBS (1 M in toluene, 0.05 mL, 0.05 mmol) in dry THF (5 mL), BH_3_·DMS (12 uL, 0.13 mmol) was slowly added at −40 °C. After being stirred for 30 min at the same temperature, a solution of **25b** (55 mg, 0.12 mmol) in THF (2 mL) was slowly added. After being stirred for 2 h at −40 °C, the mixture was diluted with MeOH. The resulting mixture was concentrated and extracted with DCM (30 mL × 3). The combined organic layers were washed with brine, dried, filtrated, and concentrated. The residue was purified by column chromatography on silica gel (PE/EA = 15:1) to give a mixture of **26c** and **26d** (3.6:1, 44 mg, 80%) as a colorless oil (pure major isomer **26c** can be obtained by repeating the purification on silica gel).

**^1^H NMR** (400 MHz, CDCl_3_) δ 6.60 (dd, *J* = 10.0, 1.3 Hz, 1H), 6.19 (dd, *J* = 15.0, 10.9 Hz, 1H), 5.96 (ddt, *J* = 17.1, 10.5, 5.6 Hz, 1H), 5.77 (d, *J* = 10.8 Hz, 1H), 5.49 (dt, *J* = 14.8, 7.3 Hz, 1H), 5.32 (dd, *J* = 17.2, 1.5 Hz, 1H), 5.23 (dd, *J* = 10.4, 1.2 Hz, 1H), 4.64 (d, *J* = 5.5 Hz, 2H), 4.23–4.07 (m, 2H), 3.36 (s, 1H), 2.67–2.49 (m, 1H), 2.35 (dd, *J* = 13.3, 6.2 Hz, 1H), 2.29–2.21 (m, 1H), 2.14 (t, *J* = 7.0 Hz, 2H), 1.84 (d, *J* = 1.1 Hz, 3H), 1.71 (s, 3H), 1.64–1.56 (m, 1H), 1.49 (ddd, *J* = 14.5, 9.5, 3.7 Hz, 1H), 1.14 (d, *J* = 6.2 Hz, 3H), 1.01 (d, *J* = 6.6 Hz, 3H), 0.88 (s, 9H), 0.06 (d, *J* = 20.4 Hz, 6H). **^13^C NMR** (101 MHz, CDCl_3_) δ 168.09, 147.74, 133.01, 132.71, 130.27, 128.27, 127.94, 126.52, 117.86, 70.43, 65.29, 64.58, 46.92, 43.25, 40.04, 33.93, 25.95, 23.98, 19.73, 18.06, 17.21, 12.73, −4.52, −4.71. ${\left[\alpha \right]}_{D}^{20}$ = −14.83, (*c* 0.1, CHCl_3_). HRMS (ESI+): calcd. for C_26_H_46_O_4_Si [M + H]^+^, 451.3238, found 451.3240.

### 4.23. Allyl (2E,4R,6E,8E,11S,13S)-11-((Tert-Butyldimethylsilyl)Oxy)-13-Hydroxy-2,4,9-Trimethyltetradeca-2,6,8-Trienoate (**26d**)

To a solution of (*R*)-Me-CBS (1 M in toluene, 0.1 mL, 0.1 mmol) in dry THF (5 mL), BH_3_·DMS (24 uL, 0.25 mmol) was slowly added at −40 °C. After being stirred for 30 min at the same temperature, a solution of **25b** (113 mg, 0.25 mmol) in THF (2 mL) was slowly added. After being stirred for 2 h at −40 °C, the mixture was diluted with MeOH. The resulting mixture was concentrated and extracted with DCM (30 mL × 3). The combined organic layers were washed with brine, dried, filtrated, and concentrated. The residue was purified by column chromatography on silica gel (PE/EA = 15:1) to provide **26d** (100 mg, 88%) as a colorless oil.

**^1^H NMR** (400 MHz, CDCl_3_) δ 6.60 (dd, *J* = 10.0, 1.3 Hz, 1H), 6.19 (dd, *J* = 15.0, 10.8 Hz, 1H), 5.96 (ddt, *J* = 17.1, 10.5, 5.6 Hz, 1H), 5.76 (d, *J* = 10.8 Hz, 1H), 5.50 (dt, *J* = 14.8, 7.3 Hz, 1H), 5.32 (dd, *J* = 17.2, 1.5 Hz, 1H), 5.23 (dd, *J* = 10.4, 1.3 Hz, 1H), 4.64 (d, *J* = 5.5 Hz, 2H), 4.11–3.98 (m, 1H), 3.95–3.82 (m, 1H), 3.12 (s, 1H), 2.67–2.51 (m, 1H), 2.32 (dd, *J* = 13.2, 4.7 Hz, 1H), 2.19–2.06 (m, 3H), 1.84 (d, *J* = 1.2 Hz, 3H), 1.71 (s, 3H), 1.56 (dt, *J* = 14.4, 3.2 Hz, 1H), 1.46–1.38 (m, 1H), 1.14 (d, *J* = 6.2 Hz, 3H), 1.02 (d, *J* = 6.7 Hz, 3H), 0.89 (s, 9H), 0.11 (d, *J* = 8.9 Hz, 6H). **^13^C NMR** (101 MHz, CDCl_3_) δ 168.10, 147.74, 132.78, 132.71, 130.32, 128.22, 127.91, 126.53, 117.84, 72.26, 67.41, 65.28, 49.21, 44.81, 40.03, 33.90, 25.95, 23.70, 19.72, 18.02, 17.33, 12.74, −3.78, −4.56. ${\left[\alpha \right]}_{D}^{20}$ = −15.29, (*c* 0.63, CHCl_3_). HRMS (ESI+): calcd. for C_26_H_46_O_4_Si [M + H]^+^, 451.3238, found 451.3228.

### 4.24. Synthetic Procedure of **27a**–**27d**

To a stirred solution of **26a** (35 mg) in dry THF (2 mL), the HF/Py complex (0.7 mL) was added at 0 °C. After being stirred for 1 h, the mixture was quenched with a saturated aqueous solution of NaHCO_3_ and extracted with DCM (10 mL × 3). The combined organic layers were washed with 1 M HCl, brine, dried over Na_2_SO_4_, filtrated, and concentrated. The residue was purified by a column chromatography on silica gel (PE/EA = 2:1) to create **27a** (22 mg, 84%) as a colorless oil.

^1^H NMR (400 MHz, CDCl_3_) δ 6.64–6.56 (m, 1H), 6.23 (dd, *J* = 15.0, 10.8 Hz, 1H), 5.96 (ddt, *J* = 17.1, 10.5, 5.6 Hz, 1H), 5.84 (d, *J* = 10.8 Hz, 1H), 5.54 (dt, *J* = 14.8, 7.3 Hz, 1H), 5.32 (dd, *J* = 17.2, 1.4 Hz, 1H), 5.23 (dd, *J* = 10.5, 0.9 Hz, 1H), 4.64 (d, *J* = 5.5 Hz, 2H), 4.13–4.02 (m, 1H), 4.02–3.92 (m, 1H), 2.65–2.51 (m, 1H), 2.24–2.10 (m, 4H), 1.84 (d, *J* = 1.3 Hz, 3H), 1.75 (s, 3H), 1.61–1.55 (m, 1H), 1.55–1.47 (m, 1H), 1.20 (d, *J* = 6.2 Hz, 3H), 1.02 (d, *J* = 6.7 Hz, 3H). ^13^C NMR (101 MHz, CDCl_3_) δ 168.11, 147.65, 132.71, 132.58, 130.99, 128.31, 128.00, 126.60, 117.87, 70.45, 68.91, 65.30, 48.84, 44.80, 40.00, 33.83, 24.07, 19.72, 16.88, 12.75. ${\left[\alpha \right]}_{D}^{20}$ = −29.76, (*c* 0.34, CHCl_3_). HRMS (ESI+): calcd. for C_20_H_32_O_4_ [M + Na]^+^,337.2373, found 337.2375.

Compound **27b** (20.9 mg, 80%) was obtained as a colorless oil. ^1^H NMR (400 MHz, CDCl_3_) δ 6.60 (dd, *J* = 10.0, 1.3 Hz, 1H), 6.23 (dd, *J* = 15.0, 10.8 Hz, 1H), 5.96 (ddt, *J* = 17.1, 10.5, 5.6 Hz, 1H), 5.85 (d, *J* = 10.7 Hz, 1H), 5.54 (dt, *J* = 14.8, 7.3 Hz, 1H), 5.32 (dd, *J* = 17.2, 1.5 Hz, 1H), 5.23 (dd, *J* = 10.4, 1.2 Hz, 1H), 4.64 (d, *J* = 5.5 Hz, 2H), 4.21–4.12 (m, 1H), 4.08 (ddd, *J* = 12.4, 7.8, 5.3 Hz, 1H), 2.65–2.51 (m, 1H), 2.22–2.18 (m, 2H), 2.16 (d, *J* = 7.2 Hz, 2H), 1.84 (d, *J* = 1.3 Hz, 3H), 1.76 (s, 3H), 1.67–1.54 (m, 2H), 1.23 (d, *J* = 6.3 Hz, 3H), 1.02 (d, *J* = 6.7 Hz, 3H). ^13^C NMR (101 MHz, CDCl_3_) δ 168.11, 147.65, 133.00, 132.71, 130.92, 128.15, 128.03, 126.59, 117.86, 66.73, 65.47, 65.30, 48.14, 44.09, 40.01, 33.84, 23.69, 19.72, 16.81, 12.75. ${\left[\alpha \right]}_{D}^{20}$ = −14.00, (*c* 0.05, CHCl_3_). HRMS (ESI+): calcd. for C_20_H_32_O_4_ [M + Na]^+^,337.2373, found 337.2377.

Compound **27c** (22 mg, 84%) was obtained as a colorless oil. ^1^H NMR (400 MHz, CDCl_3_) δ 6.60 (dd, *J* = 10.0, 1.3 Hz, 1H), 6.23 (dd, *J* = 14.9, 10.8 Hz, 1H), 5.96 (ddt, *J* = 17.1, 10.5, 5.6 Hz, 1H), 5.84 (d, *J* = 10.8 Hz, 1H), 5.54 (dt, *J* = 14.9, 7.3 Hz, 1H), 5.33 (dd, *J* = 17.2, 1.5 Hz, 1H), 5.23 (dd, *J* = 10.4, 1.2 Hz, 1H), 4.64 (d, *J* = 5.5 Hz, 2H), 4.10–4.02 (m, 1H), 4.01–3.94 (m, 1H), 2.62–2.52 (m, 1H), 2.20–2.11 (m, 4H), 1.84 (d, *J* = 1.3 Hz, 3H), 1.75 (s, 3H), 1.61–1.55 (m, 1H), 1.54–1.46 (m, 1H), 1.20 (d, *J* = 6.2 Hz, 3H), 1.02 (d, *J* = 6.7 Hz, 3H). ^13^C NMR (101 MHz, CDCl_3_) δ 168.12, 147.64, 132.71, 132.57, 130.99, 128.32, 128.01, 126.60, 117.87, 70.45, 68.91, 65.31, 48.84, 44.81, 39.99, 33.85, 24.07, 19.70, 16.88, 12.75. ${\left[\alpha \right]}_{D}^{20}$ = −29.37, (*c* 0.32, CHCl_3_). HRMS (ESI+): calcd. for C_20_H_32_O_4_ [M + Na]^+^,337.2373, found 337.2374.

Compound **27d** (21.4 mg, 82%) was obtained as a colorless oil. ^1^H NMR (400 MHz, CDCl_3_) δ 6.23 (dd, *J* = 15.0, 10.8 Hz, 1H), 5.96 (ddt, *J* = 17.1, 10.5, 5.6 Hz, 1H), 5.86 (d, *J* = 10.8 Hz, 1H), 5.54 (dt, *J* = 14.8, 7.3 Hz, 1H), 5.33 (dd, *J* = 17.2, 1.5 Hz, 1H), 5.23 (dd, *J* = 10.4, 1.3 Hz, 1H), 4.64 (d, *J* = 5.5 Hz, 2H), 4.21–4.11 (m, 1H), 4.12–4.02 (m, 1H), 2.65–2.51 (m, 1H), 2.23–2.18 (m, 2H), 2.15 (t, *J* = 7.2 Hz, 2H), 1.84 (d, *J* = 1.3 Hz, 3H), 1.76 (s, 3H), 1.65–1.56 (m, 2H), 1.23 (d, *J* = 6.3 Hz, 3H), 1.02 (d, *J* = 6.6 Hz, 3H). ^13^C NMR (101 MHz, CDCl_3_) δ 168.12, 147.64, 132.99, 132.72, 130.95, 128.18, 128.04, 126.60, 117.87, 66.72, 65.48, 65.31, 48.14, 44.10, 39.99, 33.86, 23.69, 19.70, 16.82, 12.76. ${\left[\alpha \right]}_{D}^{20}$ = −17.62, (*c* 0.32, CHCl_3_). HRMS (ESI+): calcd. for C_20_H_32_O_4_ [M + Na]^+^, 337.2373, found 337.2374.

### 4.25. Synthetic Procedure of **28a**–**28d**

To a stirred solution of compound **27a** (22 mg, 0.065 mmol) in dry acetone (2 mL) under argon, PTSA· H_2_O (1.14 mg, 0.006 mmol) and 2,2-dimethylpropane (0.08 mL, 0.65 mmol) were added sequentially. After 1 h, the reaction mixture was quenched with a saturated aqueous solution of NaHCO_3_ and extracted with DCM (2 × 10 mL). The combined organic extracts were washed with water and brine, dried over Na_2_SO_4_, and concentrated in vacuum. Purification by column chromatography (PE/EA = 20:1) provided **28a** (23 mg, 93%) as a colorless oil.

^1^H NMR (400 MHz, CDCl_3_) δ 6.61 (dd, *J* = 9.9, 1.3 Hz, 1H), 6.23 (dd, *J* = 15.0, 10.8 Hz, 1H), 5.96 (ddt, *J* = 17.1, 10.5, 5.6 Hz, 1H), 5.80 (d, *J* = 10.8 Hz, 1H), 5.50 (dt, *J* = 14.8, 7.3 Hz, 1H), 5.32 (dd, *J* = 17.2, 1.5 Hz, 1H), 5.23 (dd, *J* = 10.4, 1.3 Hz, 1H), 4.64 (d, *J* = 5.5 Hz, 2H), 4.04–3.88 (m, 2H), 2.65–2.50 (m, 1H), 2.30 (dd, *J* = 13.6, 5.9 Hz, 1H), 2.15 (t, *J* = 7.1 Hz, 2H), 2.06 (dd, *J* = 13.6, 7.0 Hz, 1H), 1.84 (d, *J* = 1.1 Hz, 3H), 1.74 (s, 3H), 1.49–1.42 (m, 4H), 1.41–1.34 (m, 4H), 1.15 (d, *J* = 6.1 Hz, 3H), 1.02 (d, *J* = 6.7 Hz, 3H). ^13^C NMR (101 MHz, CDCl_3_) δ 168.11, 147.77, 133.12, 132.72, 129.98, 128.39, 127.15, 126.52, 117.83, 98.61, 67.97, 65.34, 65.27, 46.85, 40.00, 38.60, 33.90, 30.46, 22.38, 19.94, 19.69, 17.34, 12.74. ${\left[\alpha \right]}_{D}^{20}$ = −30.22, (*c* 0.26, CHCl_3_). HRMS (ESI+): calcd. for C_23_H_36_O_4_ [M + Na]^+^,377.2686, found 377.2689.

Compound **28b** (20.8 mg, 89%) was obtained as a colorless oil. ^1^H NMR (400 MHz, CDCl_3_) δ 6.61 (dd, *J* = 10.0, 1.3 Hz, 1H), 6.23 (dd, *J* = 15.0, 10.8 Hz, 1H), 5.96 (ddt, *J* = 17.1, 10.5, 5.6 Hz, 1H), 5.81 (d, *J* = 10.7 Hz, 1H), 5.49 (dt, *J* = 14.8, 7.3 Hz, 1H), 5.33 (dd, *J* = 17.2, 1.5 Hz, 1H), 5.23 (dd, *J* = 10.4, 1.3 Hz, 1H), 4.64 (d, *J* = 5.5 Hz, 2H), 4.04–3.88 (m, 2H), 2.64–2.51 (m, 1H), 2.31 (dd, *J* = 14.1, 6.9 Hz, 1H), 2.22–2.07 (m, 3H), 1.84 (d, *J* = 1.3 Hz, 3H), 1.74 (s, 3H), 1.63–1.57 (m, 2H), 1.35 (s, 6H), 1.18 (d, *J* = 6.3 Hz, 3H), 1.02 (d, *J* = 6.7 Hz, 3H). ^13^C NMR (101 MHz, CDCl_3_) δ 168.13, 147.79, 133.39, 132.74, 129.93, 128.43, 126.83, 126.52, 117.84, 100.32, 65.40, 65.28, 62.94, 46.16, 40.00, 39.83, 33.93, 25.24, 25.13, 21.86, 19.70, 17.07, 12.75. ${\left[\alpha \right]}_{D}^{20}$ = −47.32, (*c* 0.11, CHCl_3_). HRMS (ESI+): calcd. for C_23_H_36_O_4_ [M + Na]^+^, 377.2686, found 377.2684.

Compound **28c** (22 mg, 90%) was obtained as a colorless oil. ^1^H NMR (400 MHz, CDCl_3_) δ 6.61 (dd, *J* = 9.9, 1.3 Hz, 1H), 6.23 (dd, *J* = 15.0, 10.8 Hz, 1H), 5.96 (ddt, *J* = 17.1, 10.5, 5.6 Hz, 1H), 5.80 (d, *J* = 10.8 Hz, 1H), 5.50 (dt, *J* = 14.8, 7.3 Hz, 1H), 5.32 (dd, *J* = 17.2, 1.5 Hz, 1H), 5.23 (dd, *J* = 10.4, 1.2 Hz, 1H), 4.64 (d, *J* = 5.5 Hz, 2H), 4.02–3.88 (m, 2H), 2.66–2.50 (m, 1H), 2.30 (dd, *J* = 13.6, 5.9 Hz, 1H), 2.15 (t, *J* = 7.1 Hz, 2H), 2.06 (dd, *J* = 13.7, 7.0 Hz, 1H), 1.84 (d, *J* = 1.2 Hz, 3H), 1.74 (s, 3H), 1.49–1.42 (m, 4H), 1.41–1.34 (m, 4H), 1.15 (d, *J* = 6.1 Hz, 3H), 1.02 (d, *J* = 6.7 Hz, 3H). ^13^C NMR (101 MHz, CDCl_3_) δ 168.10, 147.76, 133.12, 132.72, 129.96, 128.39, 127.11, 126.51, 117.83, 98.60, 67.97, 65.34, 65.27, 46.81, 40.00, 38.60, 33.89, 30.45, 22.38, 19.94, 19.70, 17.35, 12.73. ${\left[\alpha \right]}_{D}^{20}$ = −25.76, (*c* 0.69, CHCl_3_). HRMS (ESI+): calcd. for C_23_H_36_O_4_ [M + Na]^+^, 377.2686, found 377.2685.

Compound **28d** (21.5 mg, 90%) was obtained as a colorless oil. ^1^H NMR (400 MHz, CDCl_3_) δ 6.61 (dd, *J* = 10.0, 1.3 Hz, 1H), 6.23 (dd, *J* = 15.0, 10.8 Hz, 1H), 5.96 (ddt, *J* = 17.1, 10.5, 5.6 Hz, 1H), 5.49 (dt, *J* = 14.8, 7.3 Hz, 1H), 5.32 (dd, *J* = 17.2, 1.5 Hz, 1H), 5.23 (dd, *J* = 10.4, 1.3 Hz, 1H), 4.64 (d, *J* = 5.5 Hz, 2H), 4.04–3.86 (m, 2H), 2.65–2.48 (m, 1H), 2.31 (dd, *J* = 14.1, 7.0 Hz, 1H), 2.20–2.06 (m, 3H), 1.84 (d, *J* = 1.3 Hz, 3H), 1.74 (s, 3H), 1.68–1.57 (m, 2H), 1.35 (s, 6H), 1.18 (d, *J* = 6.3 Hz, 3H), 1.02 (d, *J* = 6.7 Hz, 3H). ^13^C NMR (101 MHz, CDCl_3_) δ 168.11, 147.78, 133.38, 132.73, 129.90, 128.42, 126.79, 126.51, 117.83, 100.31, 65.38, 65.27, 62.93, 46.13, 40.00, 39.84, 33.92, 25.23, 25.11, 21.85, 19.70, 17.07, 12.74. ${\left[\alpha \right]}_{D}^{20}$= −17.71, (*c* 0.23, CHCl_3_). HRMS (ESI+): calcd. for C_23_H_36_O_4_ [M + Na]^+^, 377.2686, found 377.2692.

### 4.26. Synthetic Procedure of **29a**–**29d**

To a stirred solution of compound **26a** (48 mg, 0.11 mmol) in dry DCM (3 mL) and 4A molecular sieve under argon, the Proton Sponge (107 mg, 0.5 mmol) and trimethyloxonium tetrafluoroborate (60 mg, 0.4 mmol) were added sequentially. After 1 h, the reaction mixture was quenched with 1 M HCl (10 mL) and extracted with DCM (2 × 10 mL). The combined organic extracts were washed with water and brine, dried over Na_2_SO_4_, and concentrated in vacuum. Purification by column chromatography (PE/EA = 40:1) created **29a** (38 mg, 77%) as a colorless oil.

**^1^H NMR** (400 MHz, CDCl_3_) δ 6.60 (d, *J* = 9.9 Hz, 1H), 6.20 (dd, *J* = 15.0, 10.8 Hz, 1H), 5.96 (ddt, *J* = 17.1, 10.5, 5.6 Hz, 1H), 5.75 (d, *J* = 10.8 Hz, 1H), 5.47 (dt, *J* = 14.8, 7.3 Hz, 1H), 5.28 (ddd, *J* = 13.8, 11.6, 1.3 Hz, 2H), 4.64 (d, *J* = 5.6 Hz, 2H), 4.00 (tdd, *J* = 8.7, 5.4, 3.2 Hz, 1H), 3.54–3.37 (m, 1H), 3.28 (d, *J* = 2.2 Hz, 3H), 2.71–2.44 (m, 1H), 2.36–2.21 (m, 1H), 2.21–2.01 (m, 3H), 1.84 (d, *J* = 1.2 Hz, 3H), 1.72 (s, 3H), 1.62–1.54 (m, 1H), 1.37–1.28 (m, 1H), 1.11 (d, *J* = 6.1 Hz, 3H), 1.01 (d, *J* = 6.6 Hz, 3H), 0.88 (s, 9H), 0.05 (d, *J* = 6.1 Hz, 6H). **^13^C NMR** (101 MHz, CDCl_3_) δ 168.12, 147.85, 133.80, 132.75, 129.65, 128.52, 127.56, 126.47, 117.85, 73.13, 67.86, 65.27, 55.62, 49.18, 44.92, 39.99, 33.96, 26.08, 19.64, 19.38, 18.20, 17.36, 12.72, −3.96, −4.53. ${\left[\alpha \right]}_{D}^{20}$ = −38.67, (*c* 0.05, CHCl_3_). HRMS (ESI+): calcd. for C_27_H_48_O_4_Si [M + H]^+^, 465.3395, found 465.3388.

Compound **29b** (72 mg, 76%) was obtained as a colorless oil. **^1^H NMR** (500 MHz, CDCl_3_) δ 6.61 (dd, *J* = 9.9, 1.1 Hz, 1H), 6.20 (dd, *J* = 14.9, 10.9 Hz, 1H), 5.96 (ddt, *J* = 17.1, 10.5, 5.6 Hz, 1H), 5.77 (d, *J* = 10.8 Hz, 1H), 5.46 (dt, *J* = 14.8, 7.3 Hz, 1H), 5.33 (dd, *J* = 17.2, 1.5 Hz, 1H), 5.23 (dd, *J* = 10.4, 1.2 Hz, 1H), 4.64 (dd, *J* = 5.5, 1.1 Hz, 2H), 3.93–3.83 (m, 1H), 3.41 (dd, *J* = 12.5, 6.3 Hz, 1H), 3.29 (s, 3H), 2.64–2.47 (m, 1H), 2.28–2.05 (m, 4H), 1.84 (d, *J* = 1.3 Hz, 3H), 1.72 (s, 4H), 1.48–1.39 (m, 1H), 1.11 (d, *J* = 6.1 Hz, 3H), 1.01 (d, *J* = 6.7 Hz, 3H), 0.86 (s, 9H), 0.02 (d, *J* = 9.8 Hz, 6H). **^13^C NMR** (126 MHz, CDCl_3_) δ 168.14, 147.87, 133.66, 132.73, 129.73, 128.47, 127.73, 126.45, 117.85, 74.24, 68.48, 65.28, 55.97, 48.38, 44.21, 40.01, 33.97, 26.01, 19.64, 19.43, 18.16, 17.33, 12.74, −4.15, −4.47. ${\left[\alpha \right]}_{D}^{20}$ = −34.83, (*c* 0.38, CHCl_3_). HRMS (ESI+): calcd. for C_27_H_48_O_4_Si [M + H]^+^, 465.3395, found 465.3391.

Compound **29c** (32 mg, 71%) was obtained as a colorless oil. **^1^H NMR** (400 MHz, CDCl_3_) δ 6.59 (d, *J* = 9.7 Hz, 1H), 6.18 (dd, *J* = 15.0, 10.9 Hz, 1H), 5.94 (ddt, *J* = 17.1, 10.5, 5.6 Hz, 1H), 5.73 (d, *J* = 10.7 Hz, 1H), 5.45 (dt, *J* = 14.7, 7.2 Hz, 1H), 5.31 (dd, *J* = 17.2, 1.1 Hz, 1H), 5.21 (d, *J* = 10.5 Hz, 1H), 4.62 (d, *J* = 5.5 Hz, 2H), 3.98 (dt, *J* = 16.1, 6.0 Hz, 1H), 3.51–3.35 (m, 1H), 3.26 (s, 3H), 2.62–2.44 (m, 1H), 2.27 (ddd, *J* = 19.7, 13.8, 9.4 Hz, 1H), 2.18–2.01 (m, 3H), 1.82 (s, 3H), 1.70 (s, 3H), 1.60–1.52 (m, 1H), 1.37–1.25 (m, 1H), 1.09 (d, *J* = 6.1 Hz, 3H), 0.99 (d, *J* = 6.6 Hz, 3H), 0.86 (s, 9H), 0.03 (d, *J* = 6.8 Hz, 6H). **^13^C NMR** (101 MHz, CDCl_3_) δ 168.11, 147.86, 133.79, 132.74, 129.66, 128.51, 127.55, 126.46, 117.84, 73.13, 67.86, 65.27, 55.63, 49.18, 44.91, 40.05, 33.97, 26.08, 19.71, 19.38, 18.20, 17.39, 12.72, −3.96, −4.54. ${\left[\alpha \right]}_{D}^{20}$ = −26.17, (*c* 0.1, CHCl_3_). HRMS (ESI+): calcd. for C_27_H_48_O_4_Si [M + H]^+^, 465.3395, found 465.3389.

Compound **29d** (72 mg, 70%) was obtained as a colorless oil. **^1^H NMR** (400 MHz, CDCl_3_) δ 6.61 (dd, *J* = 10.0, 1.2 Hz, 1H), 6.20 (dd, *J* = 15.0, 10.9 Hz, 1H), 5.96 (ddt, *J* = 17.1, 10.5, 5.6 Hz, 1H), 5.77 (d, *J* = 10.8 Hz, 1H), 5.46 (dt, *J* = 14.7, 7.2 Hz, 1H), 5.33 (dd, *J* = 17.2, 1.5 Hz, 1H), 5.23 (dd, *J* = 10.4, 1.2 Hz, 1H), 4.64 (d, *J* = 5.5 Hz, 2H), 3.94–3.79 (m, 1H), 3.51–3.36 (m, 1H), 3.29 (s, 3H), 2.70–2.50 (m, 1H), 2.25–2.07 (m, 4H), 1.85 (d, *J* = 1.3 Hz, 3H), 1.79–1.67 (m, 4H), 1.45 (m, 1H), 1.12 (d, *J* = 6.1 Hz, 3H), 1.01 (d, *J* = 6.6 Hz, 3H), 0.86 (s, 9H), 0.01 (d, *J* = 9.0 Hz, 6H). **^13^C NMR** (101 MHz, CDCl_3_) δ 168.12, 147.87, 133.67, 132.75, 129.73, 128.47, 127.72, 126.47, 117.84, 74.25, 68.54, 65.28, 55.97, 48.39, 44.20, 40.07, 33.96, 26.02, 19.70, 19.44, 18.17, 17.37, 12.74, −4.15, −4.47. ${\left[\alpha \right]}_{D}^{20}$ = −16.46, (*c* 0.41, CHCl_3_). HRMS (ESI+): calcd. for C_27_H_48_O_4_Si [M + H]^+^, 465.3395, found 465.3389.

### 4.27. Synthetic Procedure of **1a**–**1d**

To a stirred solution of **29a** in dry THF (2 mL), the HF/Py complex (0.4 mL) was added at 0 °C. After being stirred for 1 h, the mixture was quenched with a saturated aqueous solution of NaHCO_3_ and extracted with DCM (10 mL × 3). The combined organic layers were washed with 1 M HCl, brine, dried over Na_2_SO_4_, filtrated, and concentrated. The residue was purified by column chromatography on silica gel (PE/EA = 4:1) to create **1a** (20 mg, 70%) as a colorless oil.

**^1^H NMR** (400 MHz, CDCl_3_) δ 6.60 (dd, *J* = 9.9, 1.3 Hz, 1H), 6.23 (dd, *J* = 15.0, 10.8 Hz, 1H), 5.96 (ddt, *J* = 17.1, 10.5, 5.6 Hz, 1H), 5.83 (d, *J* = 10.8 Hz, 1H), 5.59–5.42 (m, 1H), 5.32 (dd, *J* = 17.2, 1.5 Hz, 1H), 5.22 (dd, *J* = 10.4, 1.2 Hz, 1H), 4.63 (d, *J* = 5.5 Hz, 2H), 4.10–3.98 (m, 1H), 3.69–3.61 (m, 1H), 3.34 (s, 3H), 2.57 (m, 1H), 2.17 (m, 4H), 1.84 (d, *J* = 1.3 Hz, 3H), 1.75 (s, 3H), 1.59–1.55 (m, 2H), 1.17 (d, *J* = 6.2 Hz, 3H), 1.01 (d, *J* = 6.6 Hz, 3H). **^13^C NMR** (101 MHz, CDCl_3_) δ 168.11, 147.75, 133.68, 132.71, 130.36, 128.23, 127.58, 126.52, 117.85, 74.75, 66.38, 65.28, 56.34, 48.36, 42.82, 40.00, 33.89, 19.70, 18.94, 16.91, 12.73. ${\left[\alpha \right]}_{D}^{20}$ = −25.83, (*c* 0.46, CHCl_3_). HRMS (ESI+): calcd. for C_21_H_34_O_4_ [M + Na]^+^, 373.2349, found 373.2346.

Compound **1b** (40 mg, 74%) was obtained as a colorless oil. **^1^H NMR** (400 MHz, CDCl_3_) δ 6.60 (dd, *J* = 9.9, 1.1 Hz, 1H), 6.23 (dd, *J* = 15.0, 10.8 Hz, 1H), 5.95 (ddt, *J* = 17.1, 10.5, 5.6 Hz, 1H), 5.82 (d, *J* = 10.8 Hz, 1H), 5.50 (dt, *J* = 14.8, 7.3 Hz, 1H), 5.32 (dd, *J* = 17.2, 1.5 Hz, 1H), 5.22 (dd, *J* = 10.4, 1.2 Hz, 1H), 4.63 (d, *J* = 5.5 Hz, 2H), 3.98–3.87 (m, 1H), 3.61–3.49 (m, 1H), 3.33 (s, 3H), 2.65–2.47 (m, 1H), 2.26–2.05 (m, 4H), 1.83 (s, 3H), 1.75 (s, 3H), 1.59–1.50 (m, 2H), 1.17 (d, *J* = 6.0 Hz, 3H), 1.01 (d, *J* = 6.7 Hz, 3H). **^13^C NMR** (101 MHz, CDCl_3_) δ 168.10, 147.78, 133.68, 132.70, 130.13, 128.30, 127.42, 126.48, 117.82, 77.98, 69.74, 65.25, 55.89, 48.24, 43.29, 40.00, 33.88, 19.68, 19.20, 17.00, 12.71. ${\left[\alpha \right]}_{D}^{20}$ = −29.17, (*c* 0.80, CHCl_3_). HRMS (ESI+): calcd. for C_21_H_34_O_4_ [M + Na]^+^, 373.2349, found 373.2348.

Compound **1c** (18 mg, 75%) was obtained as a colorless oil. **^1^H NMR** (400 MHz, CDCl_3_) δ 6.60 (dd, *J* = 9.9, 1.3 Hz, 1H), 6.23 (dd, *J* = 15.0, 10.8 Hz, 1H), 5.96 (ddt, *J* = 17.1, 10.5, 5.6 Hz, 1H), 5.83 (d, *J* = 10.8 Hz, 1H), 5.59–5.42 (m, 1H), 5.32 (dd, *J* = 17.2, 1.5 Hz, 1H), 5.22 (dd, *J* = 10.4, 1.2 Hz, 1H), 4.63 (d, *J* = 5.5 Hz, 2H), 4.10–3.98 (m, 1H), 3.69–3.61 (m, 1H), 3.34 (s, 3H), 2.57 (m, 1H), 2.17 (m, 4H), 1.84 (d, *J* = 1.3 Hz, 3H), 1.75 (s, 3H), 1.59–1.55 (m, 2H), 1.17 (d, *J* = 6.2 Hz, 3H), 1.01 (d, *J* = 6.6 Hz, 3H). **^13^C NMR** (101 MHz, CDCl_3_) δ 168.11, 147.74, 133.68, 132.71, 130.34, 128.24, 127.58, 126.52, 117.85, 74.74, 66.38, 65.28, 56.34, 48.36, 42.87, 39.98, 33.89, 19.68, 18.96, 16.91, 12.73. ${\left[\alpha \right]}_{D}^{20}$ = −21.89, (*c* 0.52, CHCl_3_). HRMS (ESI+): calcd. for C_21_H_34_O_4_ [M + Na]^+^, 373.2349, found 373.2349.

Compound **1d** (40 mg, 74%) was obtained as a colorless oil. **^1^H NMR** (400 MHz, CDCl_3_) δ 6.60 (dd, *J* = 9.9, 1.1 Hz, 1H), 6.23 (dd, *J* = 15.0, 10.8 Hz, 1H), 5.95 (ddt, *J* = 17.1, 10.5, 5.6 Hz, 1H), 5.82 (d, *J* = 10.8 Hz, 1H), 5.50 (dt, *J* = 14.8, 7.3 Hz, 1H), 5.32 (dd, *J* = 17.2, 1.5 Hz, 1H), 5.22 (dd, *J* = 10.4, 1.2 Hz, 1H), 4.63 (d, *J* = 5.5 Hz, 2H), 3.98–3.87 (m, 1H), 3.61–3.49 (m, 1H), 3.33 (s, 3H), 2.65–2.47 (m, 1H), 2.26–2.05 (m, 4H), 1.83 (s, 3H), 1.75 (s, 3H), 1.59–1.50 (m, 2H), 1.17 (d, *J* = 6.0 Hz, 3H), 1.01 (d, *J* = 6.7 Hz, 3H). **^13^C NMR** (101 MHz, CDCl_3_) δ 168.10, 147.78, 133.69, 132.70, 130.11, 128.31, 127.41, 126.48, 117.83, 77.96, 69.75, 65.26, 55.89, 48.22, 43.30, 39.96, 33.88, 19.66, 19.20, 17.04, 12.71. ${\left[\alpha \right]}_{D}^{20}$ = −38.68, (*c* 0.76, CHCl_3_). HRMS (ESI+): calcd. for C_21_H_34_O_4_ [M + Na]^+^, 373.2349, found 373.2350. Detailed NMR data tables of **1a**–**1d** are in the Supplementary Material.

## 5. Conclusions

Asymmetric synthesis of the alotamdie fragment C15–C32 was established and four diastereomers were achieved concisely. Boron-mediated enantioselective aldol reaction led to a good diastereoselectivity and Julia-Kocienski olefination constructed the diene part in excellent *E/Z* selectivity and yield. A careful NMR comparison between four isomers and natural alotamide suggested the relative structure.

## Figures and Tables

**Table 1:**
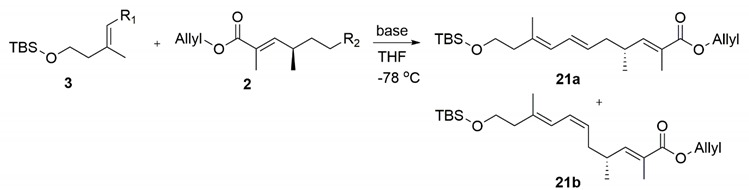
Optimization of the Julia–Kocienski olefination.

Entry	R 1	R 2	Base (equiv)	Yield ^1^	21a:21b ^2^
1	CHO	SO_2_PT	KHMDS (1eq)	50%	15:1
2	CHO	SO_2_PT	KHMDS (1.5eq)	72%	15:1
3	CHO	SO_2_PT	KHMDS (2eq)	86%	15:1
4	CHO	SO_2_PT	LiHMDS (2eq)	83%	3.5:1
5	SO_2_PT	CHO	KHMDS (2eq)	94%	1.5:1

^1^ Combined yield of **21a** and **21b**. ^2^ Determined by LC-MS.

**Table 2:**
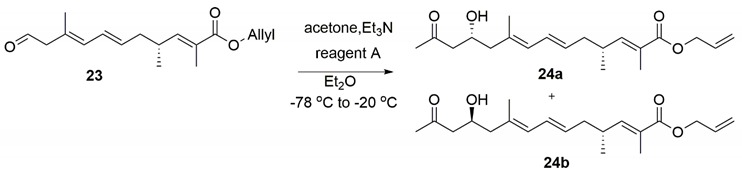
Boron-mediated aldol reaction.

Entry	Reagent A	Yield ^1^	24a:24b ^2^
1	(−)-IPCBCl	59%	1:99
2	(+)-IPCBCl	55%	98:2
3	LiHMDS	47%	1:1

^1^ Combined yield of **24a** and **24b**. ^2^ Determined by LC-MS.

**Table 3:** Δδ ^13^C (ppm) between alotamide and **1a** and **1c**.

Isomer	C20	C21	C23	C26	C29	C31
**1a**	0.30	1.90	−1.67	1.40	−0.18	−0.06
**1c**	0.28	1.88	−1.65	1.41	−0.13	−0.04

## Supplementary Materials

A supplementary file is available online at http://www.mdpi.com/1660-3397/16/11/414/s1. Supplementary Information shows the NMR spectra of the synthetic compounds and the NMR data tables of **1a**–**1d**.
