# Supplementary material for: Asymmetric Synthesis of the C15–C32 Fragment of Alotamide and Determination of the Relative Stereochemistry
Source: Mar Drugs. 2018 Oct 30;16(11):414. doi: 10.3390/md16110414 (PMC6266257; doi:10.3390/md16110414)
Supplement: Supplementary file 1 [file marinedrugs-16-00414-s001.pdf]

Supporting Information

Asymmetric Synthesis of the C15–C32 Fragment of Alotamide and Determination of the Relative Stereochemistry

Hao-yun Shi,[a,b] Yang Xie,[a] Pei Hu,[a] Zi-Qiong Guo,[a,b] Yi-hong Lu,[a,b] Yu Gao*[a] and Cheng-gang Huang*[a,b]

[a]Shanghai Institute of Materia Medica, Chinese Academy of Sciences, 501 Haike Road, Shanghai 201203, China.

[b]University of Chinese Academy of Sciences, Beijing 100049, China

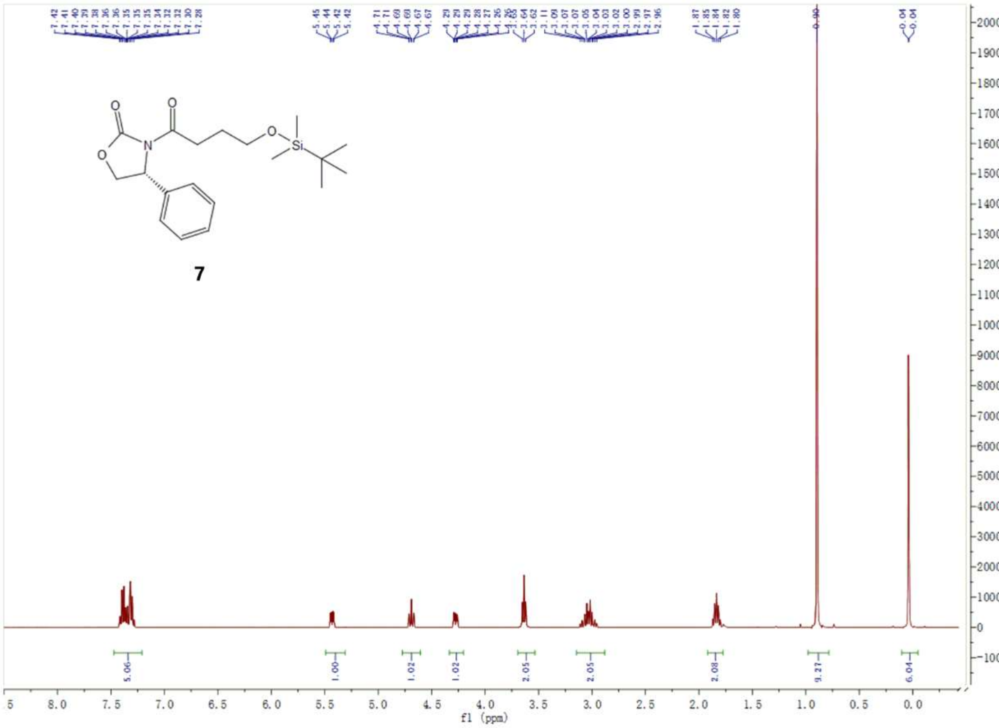
7
f1 (ppm)

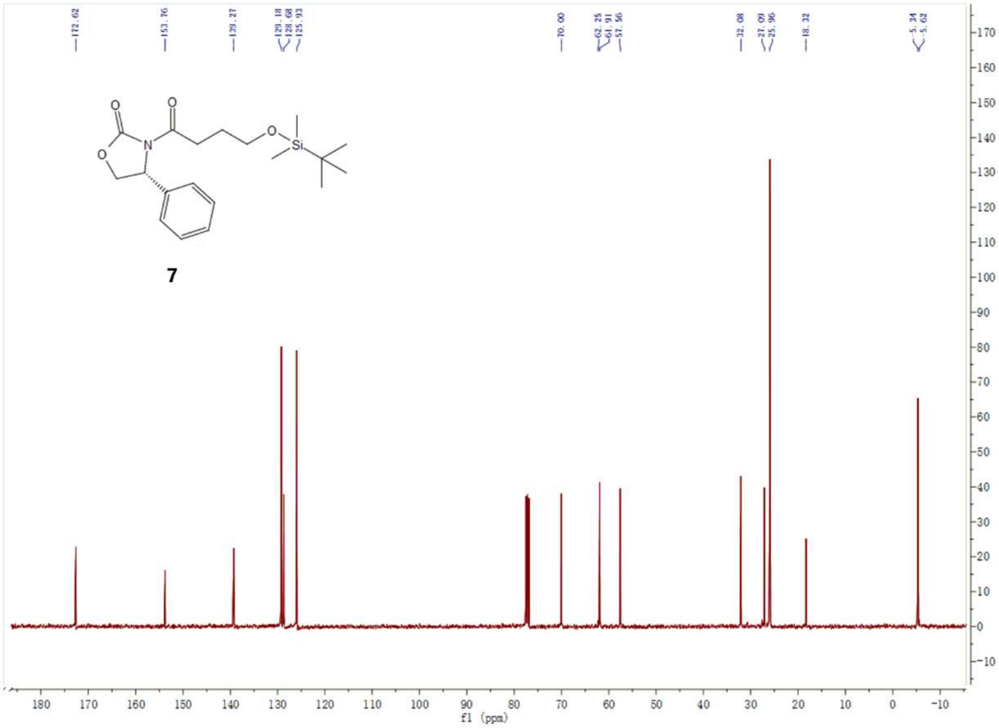
7
f1 (ppm)

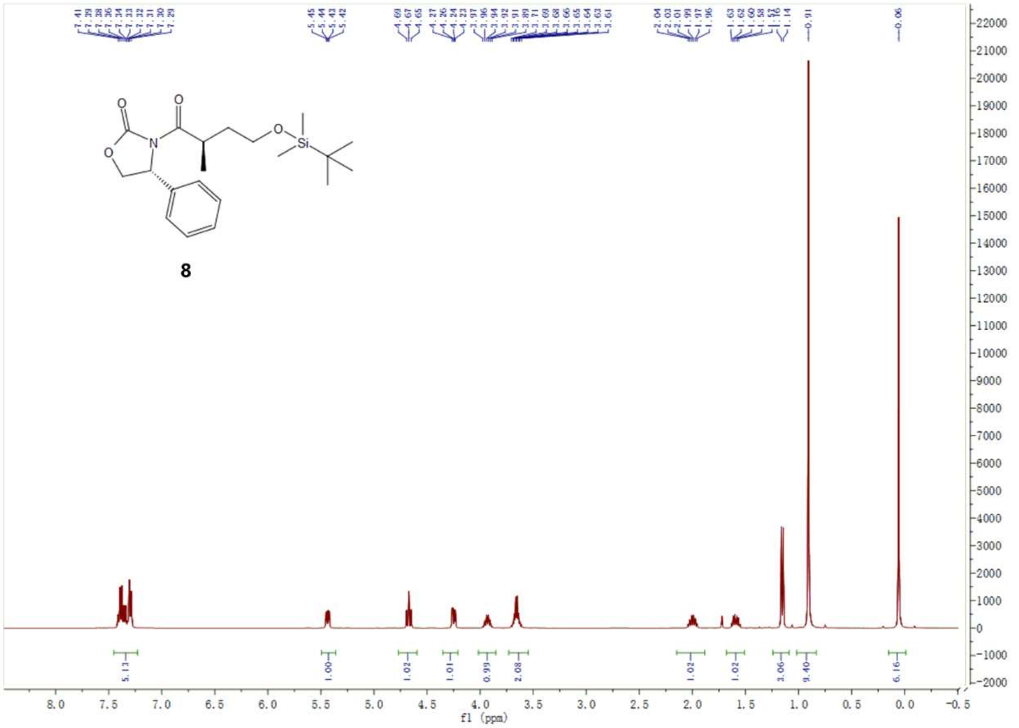
7.41 7.39 7.38 7.36 7.34 7.33 7.32 7.31 7.30 7.29
5.45 5.44 5.43 5.42
4.69 4.67 4.65 4.27 4.26 4.24 4.23 3.97 3.96 3.94 3.92 3.91 3.89 3.71 3.69 3.68 3.66 3.65 3.64 3.63 3.61
2.04 2.03 2.01 1.99 1.97 1.96
1.63 1.62 1.60 1.58 1.57 1.16 1.14
0.91
0.06
O
N
O
Si
8
5.13 1.00 1.02 1.01 0.99 2.08 1.02 1.02 3.06 9.40 6.16
f1 (ppm)

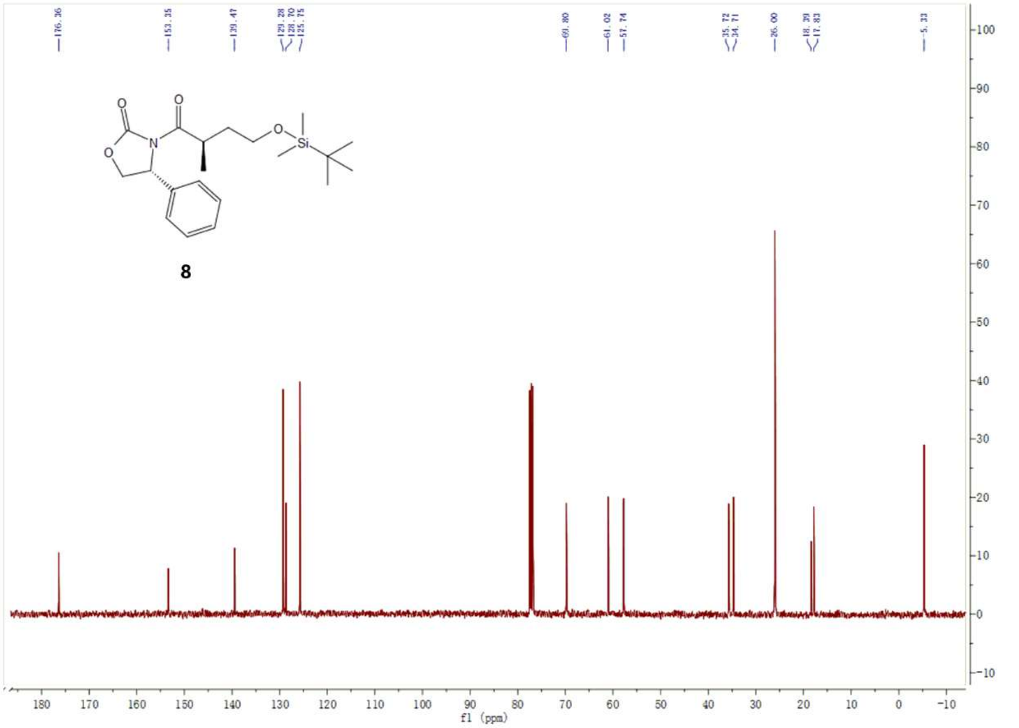
176.36
153.35
139.47
129.28 128.70 125.75
69.80
61.02
57.74
35.72 34.71
26.00
18.39 17.83
-5.33
O
N
O
Si
8
f1 (ppm)

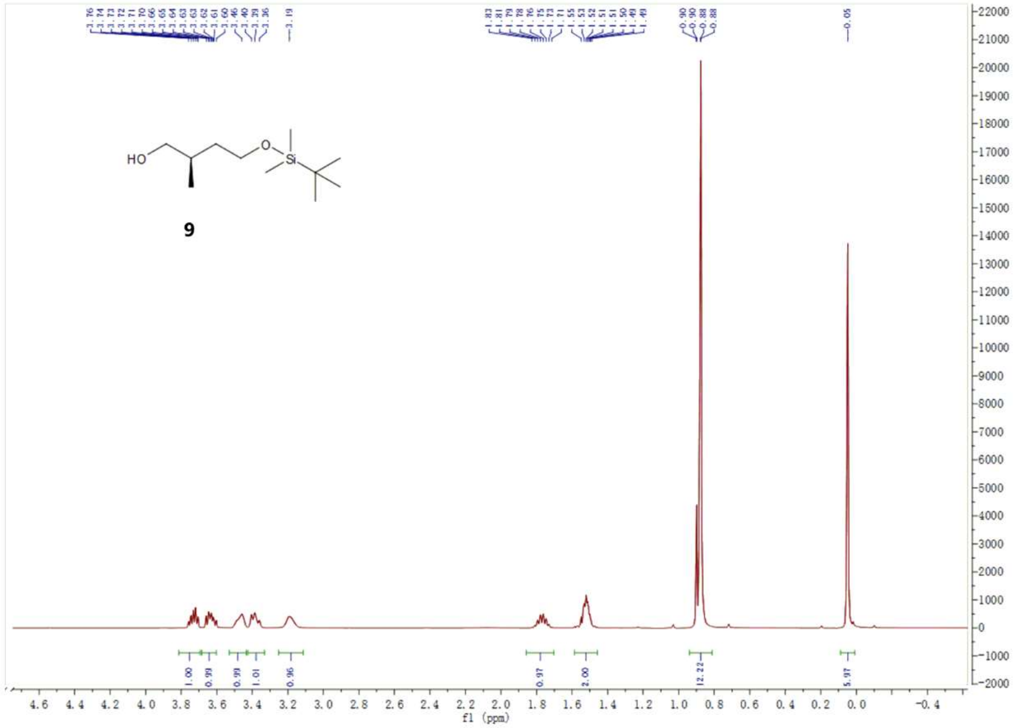

9

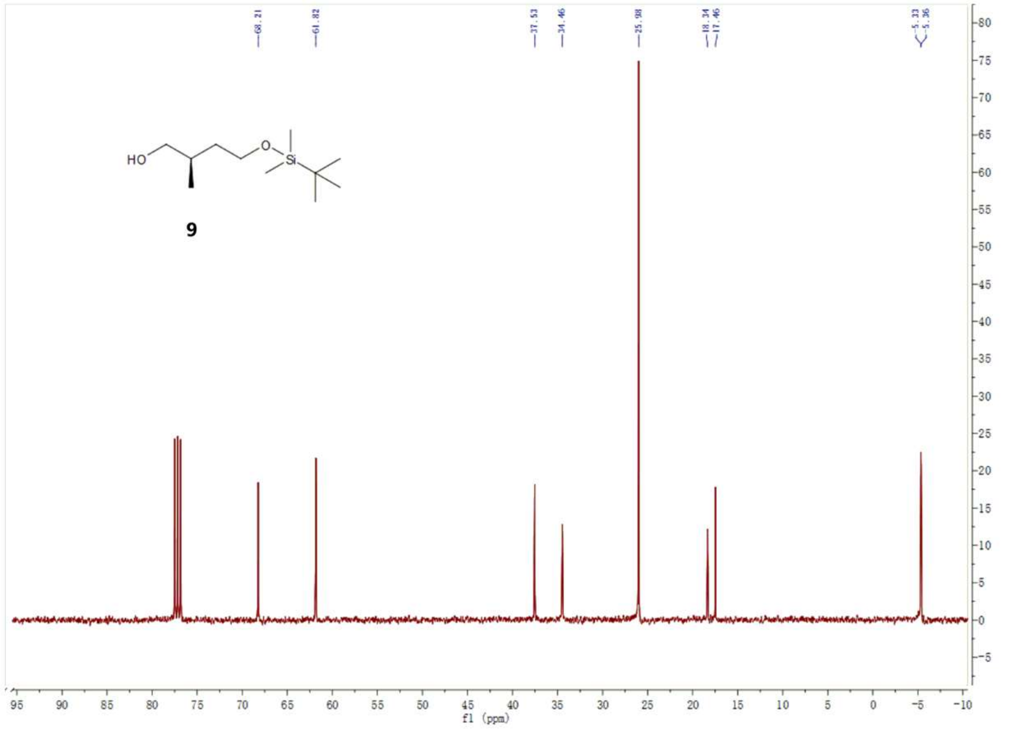

9

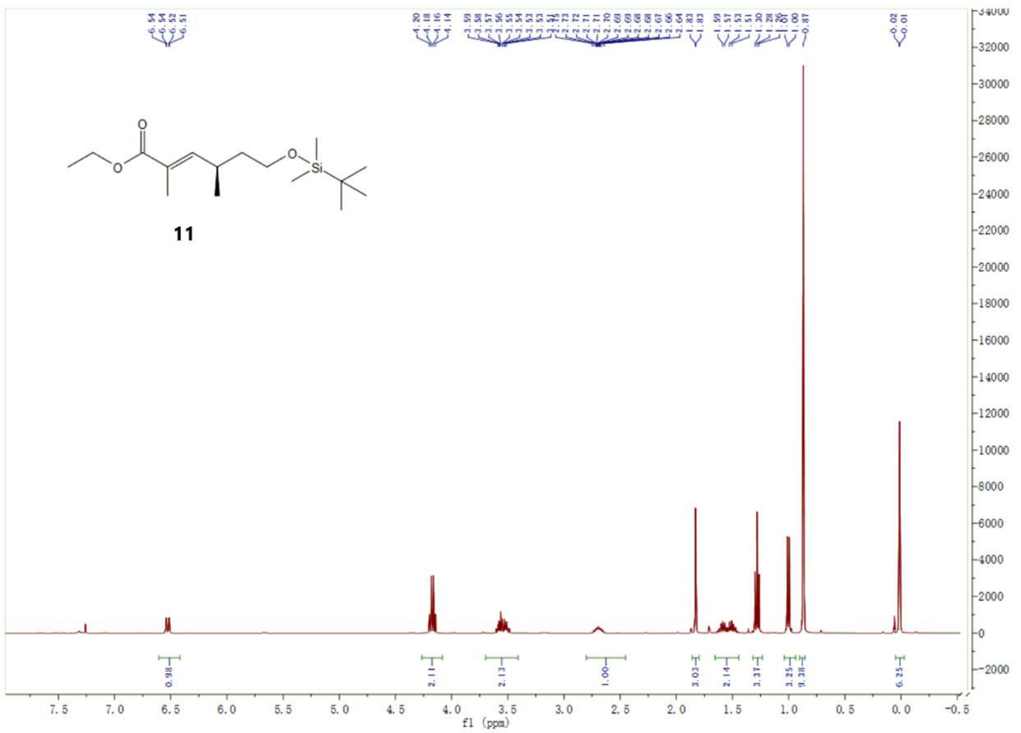
11
f1 (ppm)

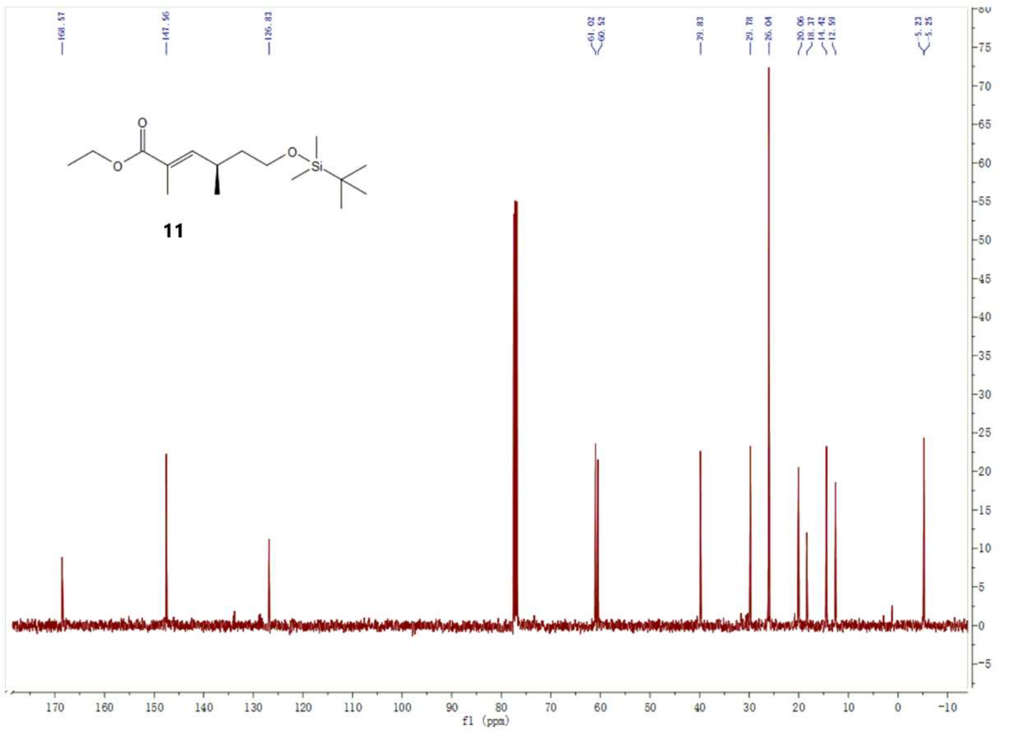
11
f1 (ppm)

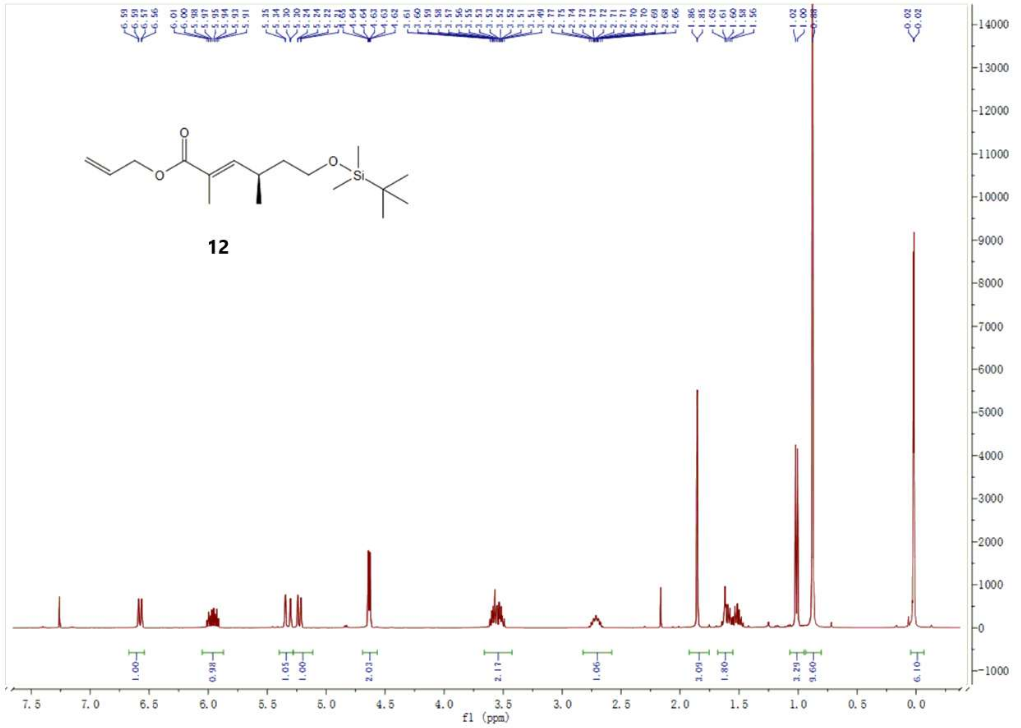

12

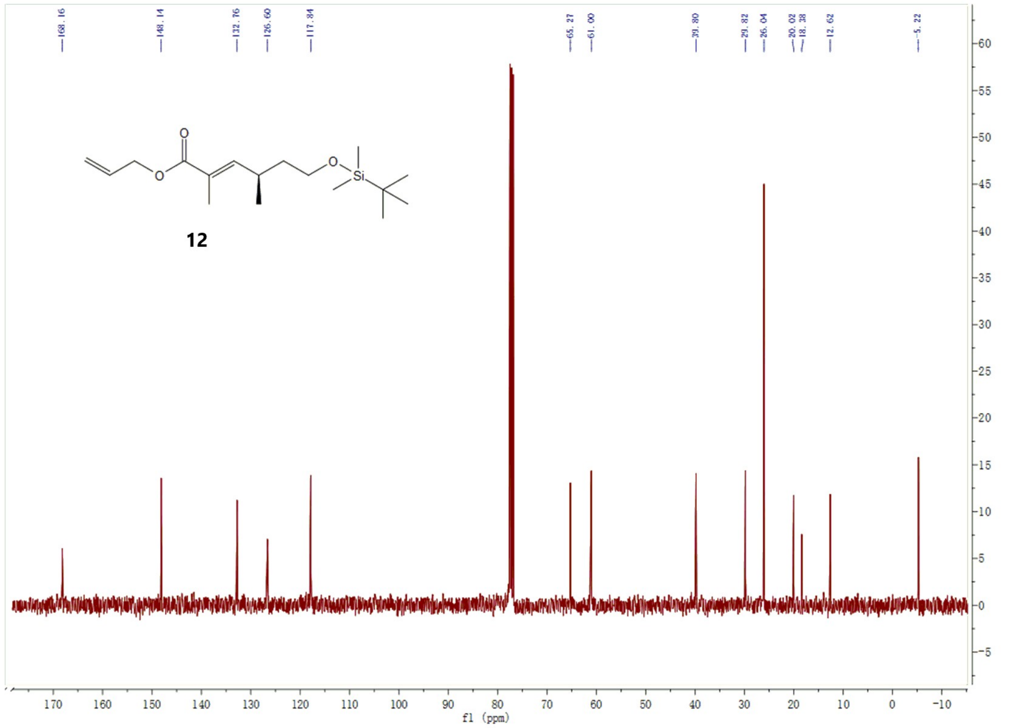

12

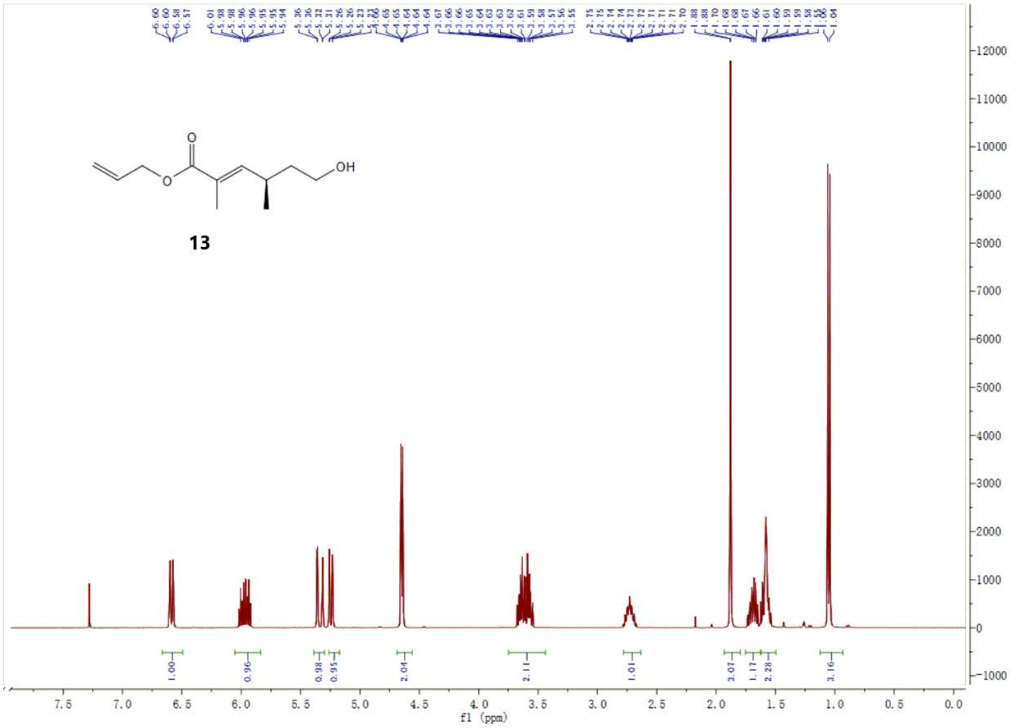
OH
O
O
13
fl (ppm)

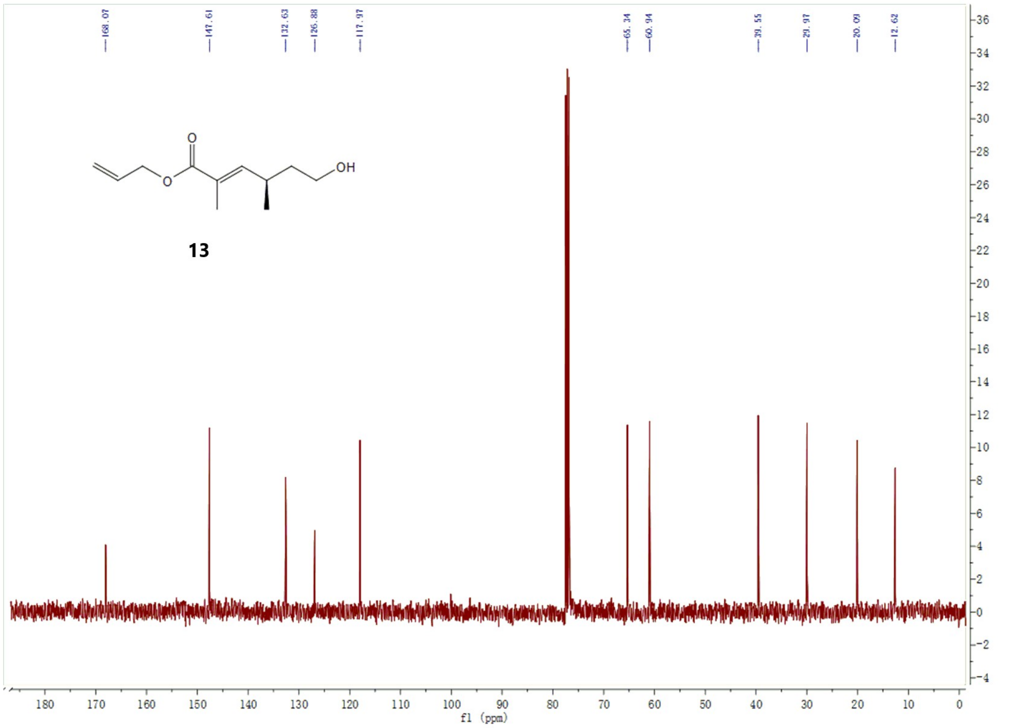
168.07
147.61
132.63
126.88
117.97
65.34
60.94
39.55
29.97
20.09
12.62
OH
O
O
13
fl (ppm)

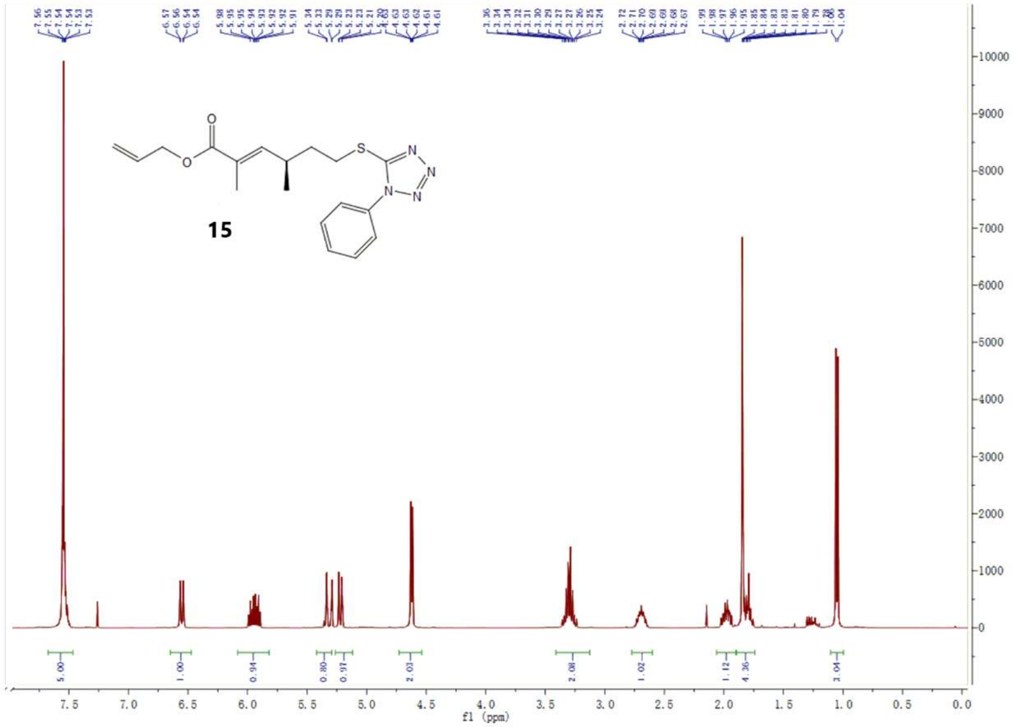

15
f1 (ppm)

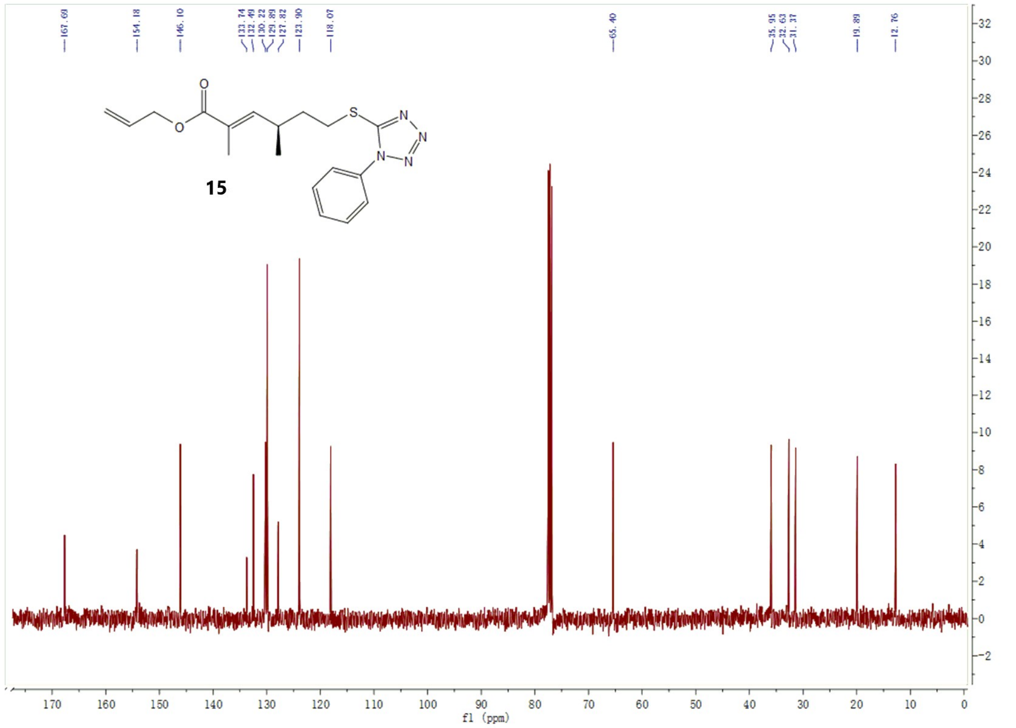

15
f1 (ppm)

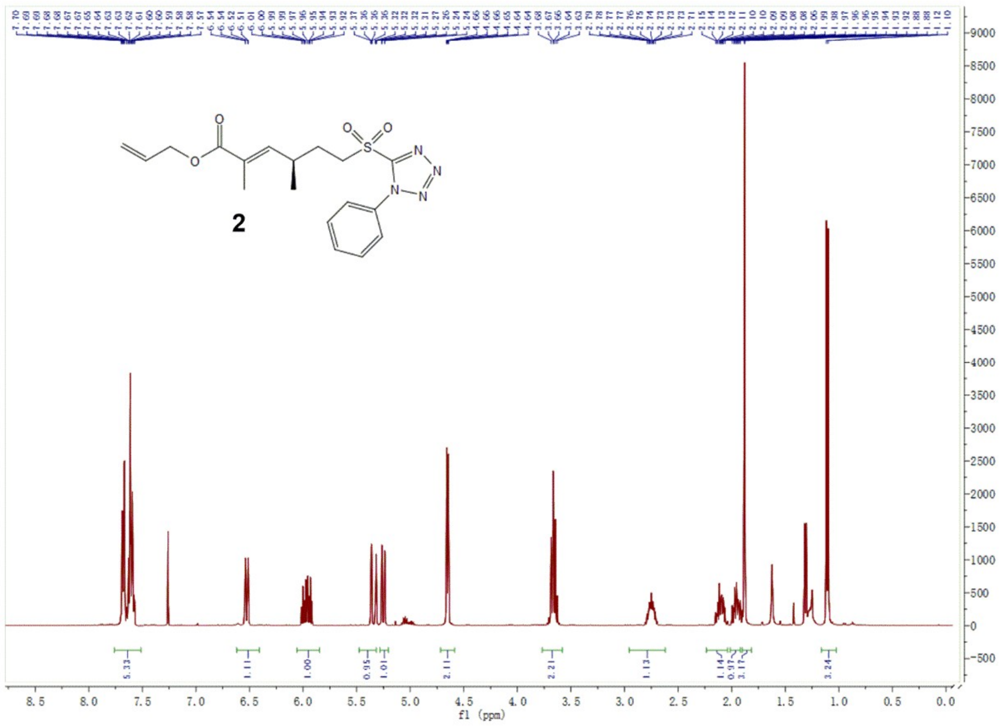
2
f1 (ppm)

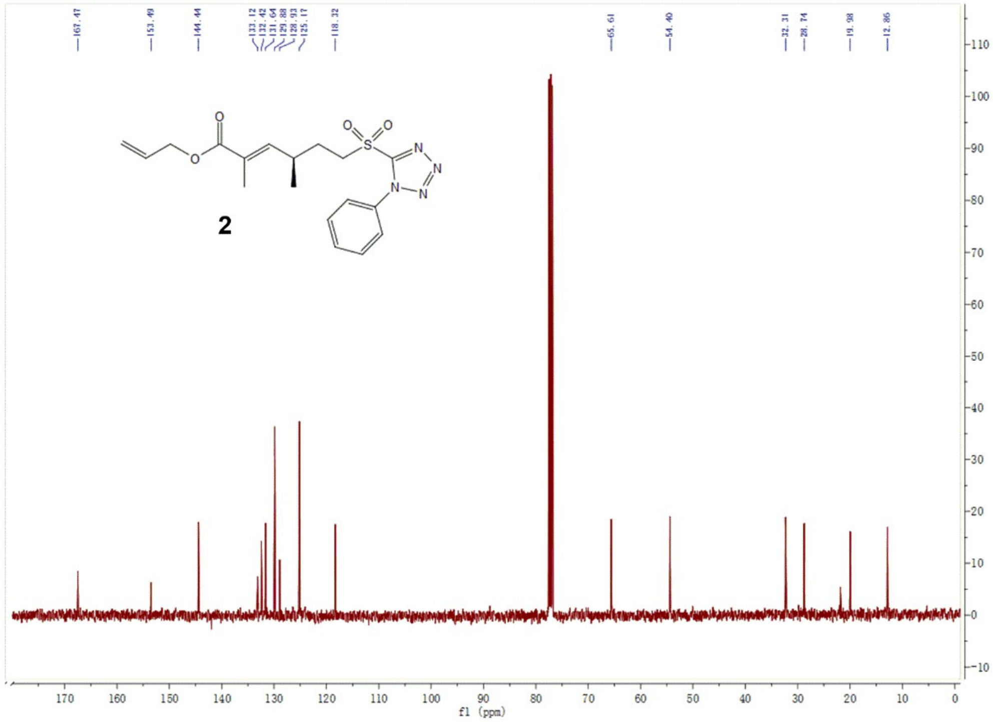
2
f1 (ppm)

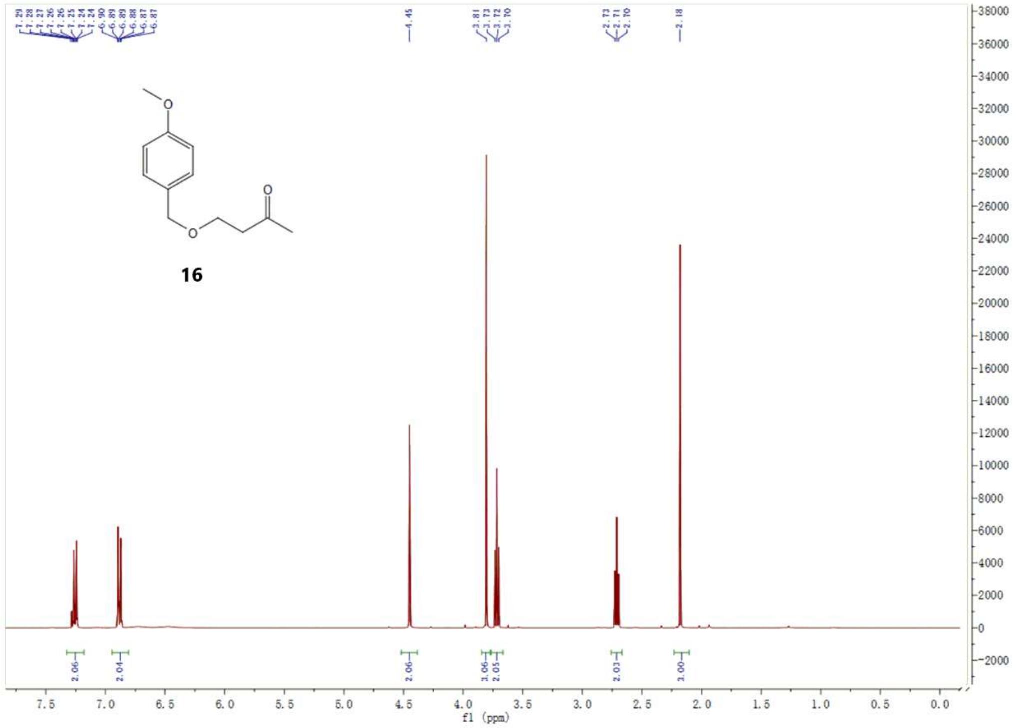

7.29
7.28
7.27
7.26
7.26
7.25
7.24
7.24
6.90
6.89
6.89
6.88
6.87
6.87
4.45
3.81
3.73
3.72
3.70
2.73
2.71
2.70
2.18
O
16
2.06
2.04
2.06
3.06
2.05
2.03
3.00
f1 (ppm)

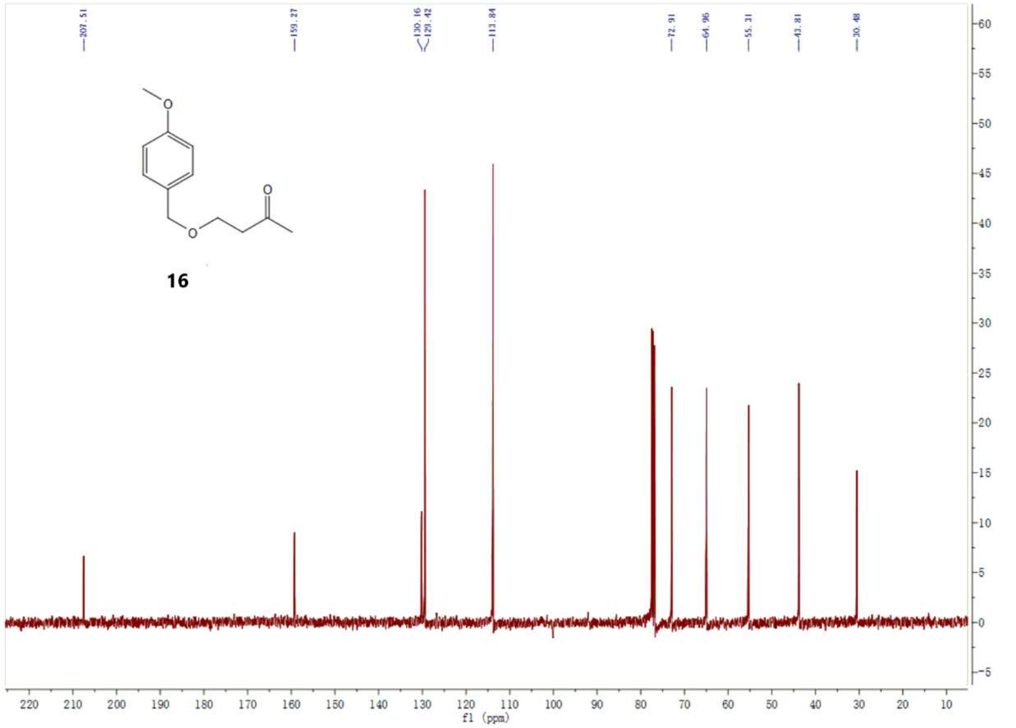

207.51
159.27
130.16
129.42
113.84
72.91
64.96
55.31
43.81
30.48
O
16
f1 (ppm)

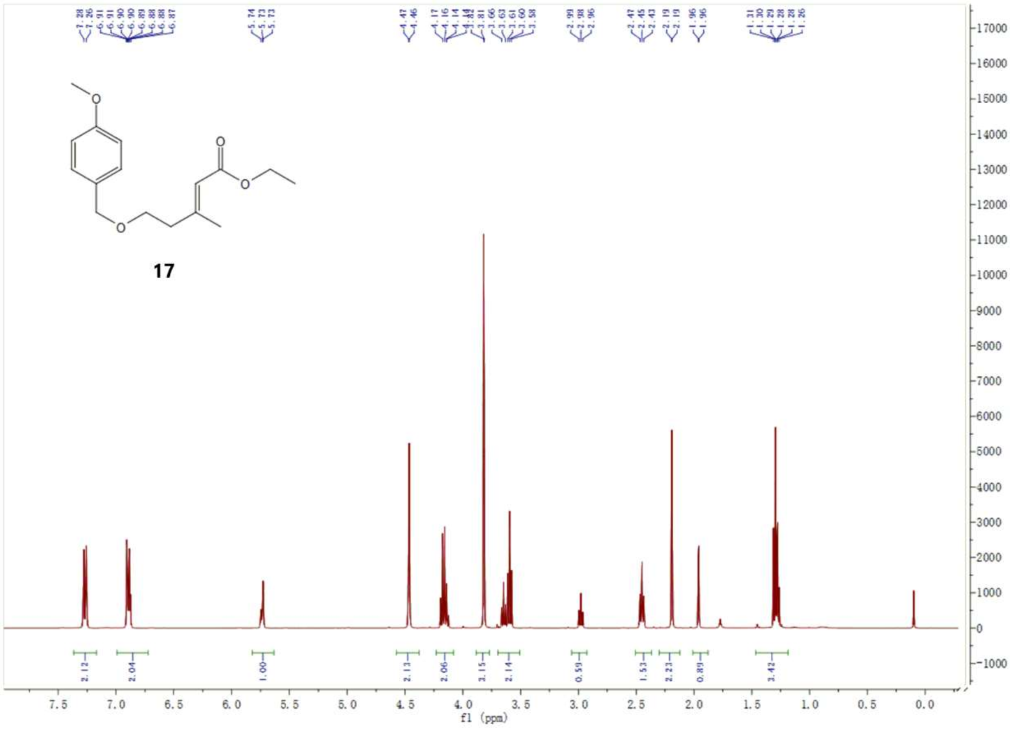

17
f1 (ppm)

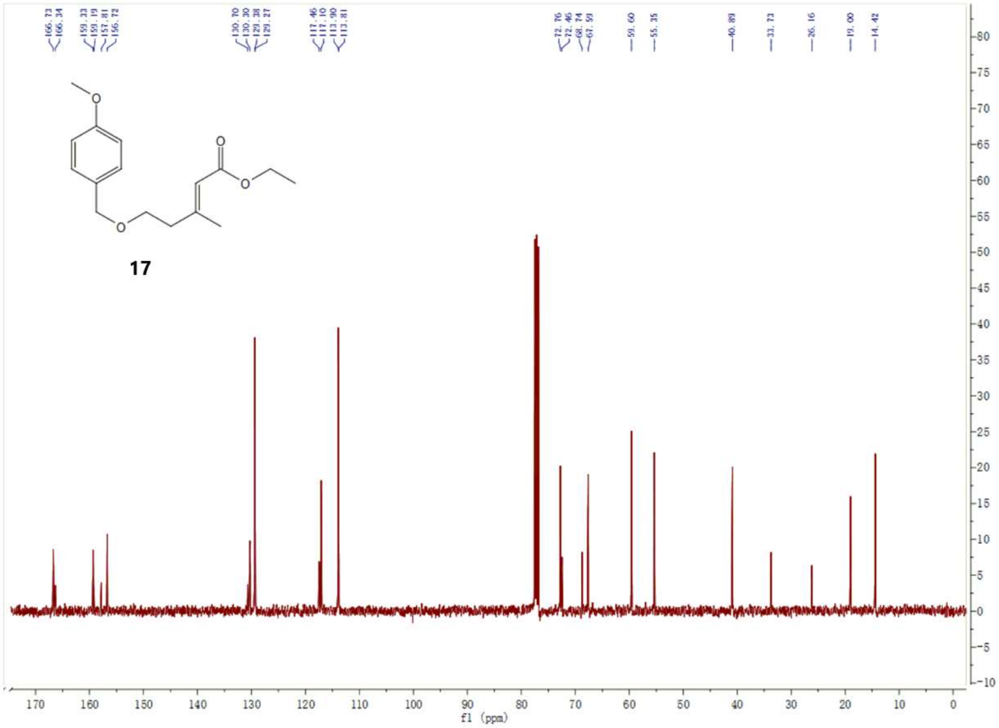

166.73
166.34
159.33
159.19
157.81
156.72
130.70
130.20
129.38
129.27
117.46
117.10
113.90
113.81
72.76
72.46
68.74
67.59
59.60
55.35
40.89
33.73
26.16
19.00
14.42
17
f1 (ppm)

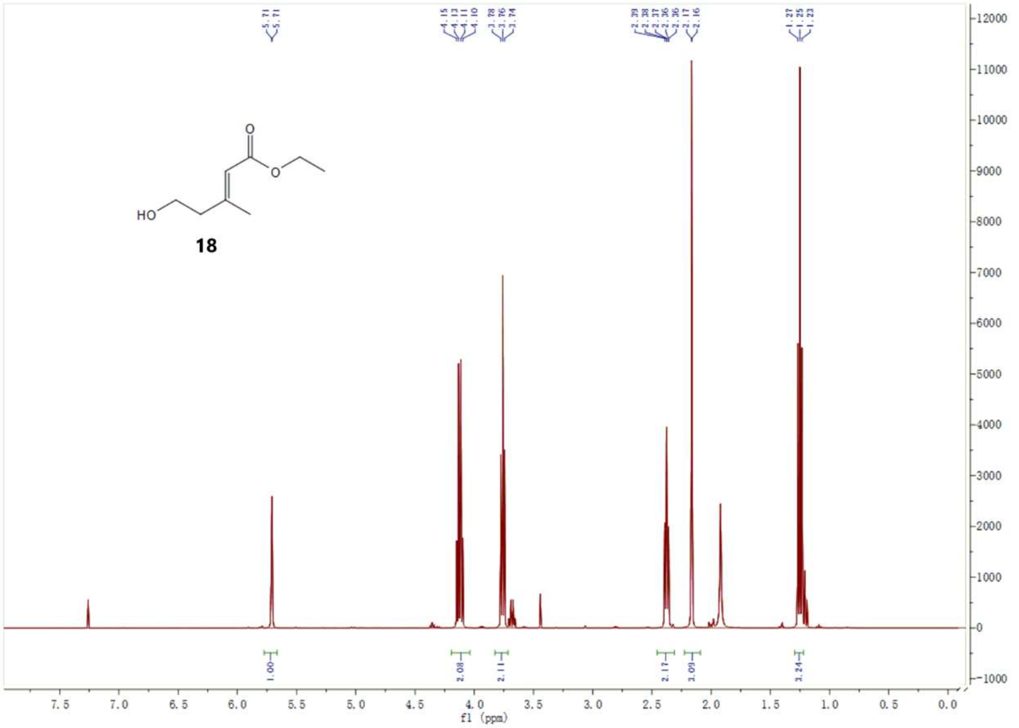

5.71
5.71
4.15
4.13
4.11
4.10
3.78
3.76
3.74
2.39
2.38
2.37
2.36
2.36
2.17
2.16
1.27
1.25
1.23
O
O
HO
18
1.00
2.08
2.11
2.11
3.09
3.24
f1 (ppm)

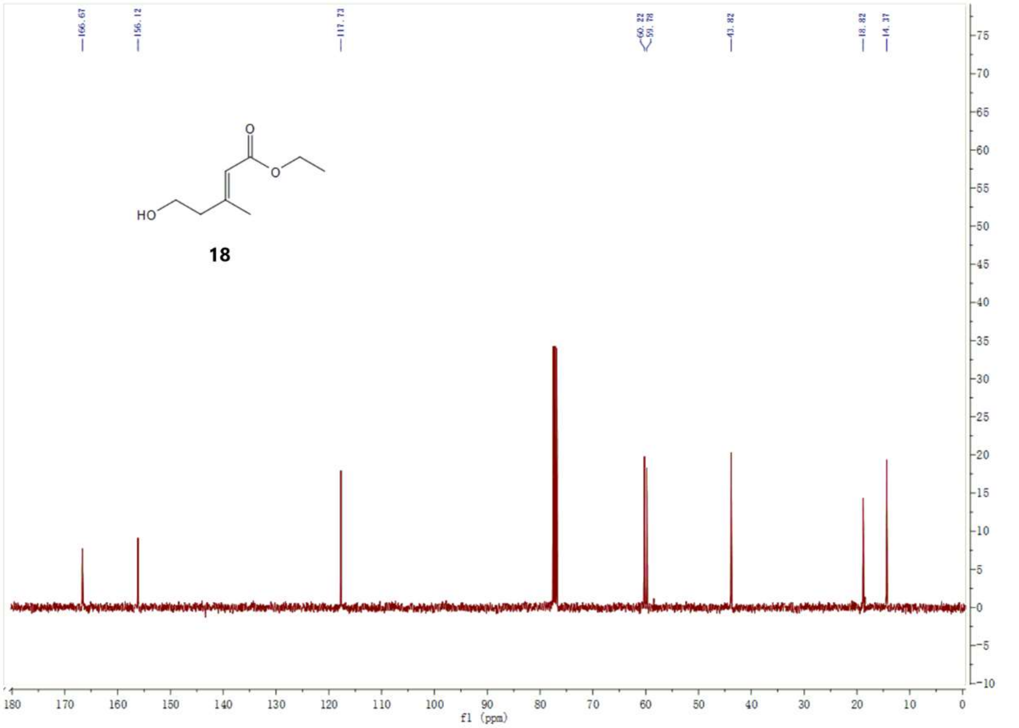

166.67
156.12
117.73
60.22
59.78
43.82
18.82
14.37
O
O
HO
18
f1 (ppm)

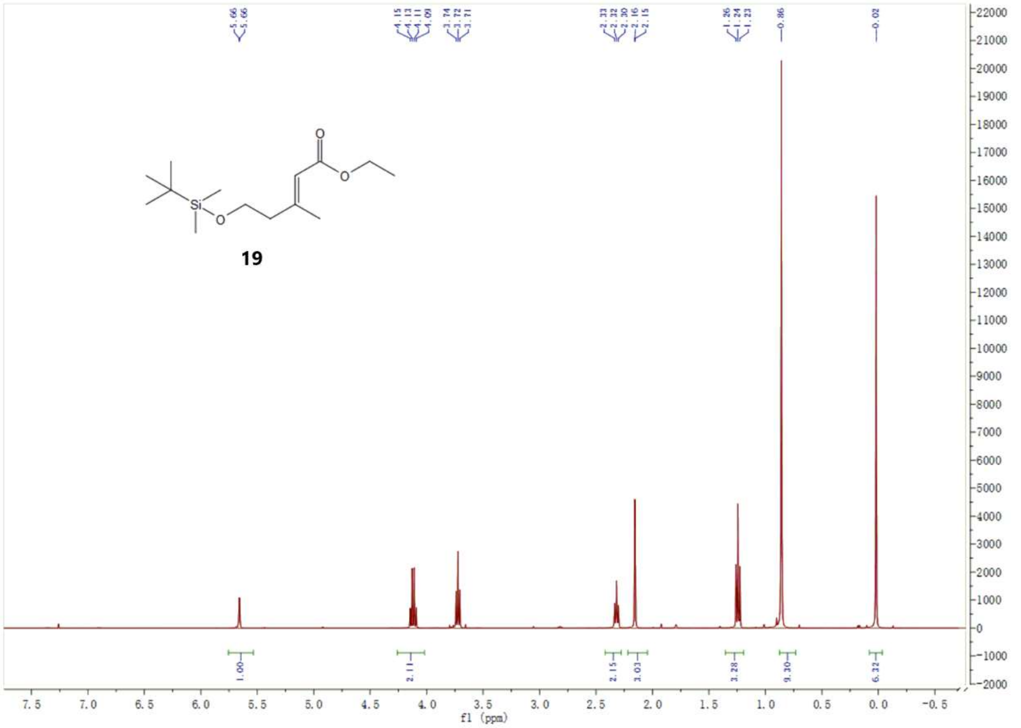

5.66
5.66
4.15
4.13
4.11
4.09
3.74
3.72
3.71
2.33
2.32
2.30
2.16
2.15
1.26
1.24
1.23
0.86
0.02
Si
O
O
O
19
1.00
2.11
2.15
3.03
3.28
9.30
6.32
f1 (ppm)

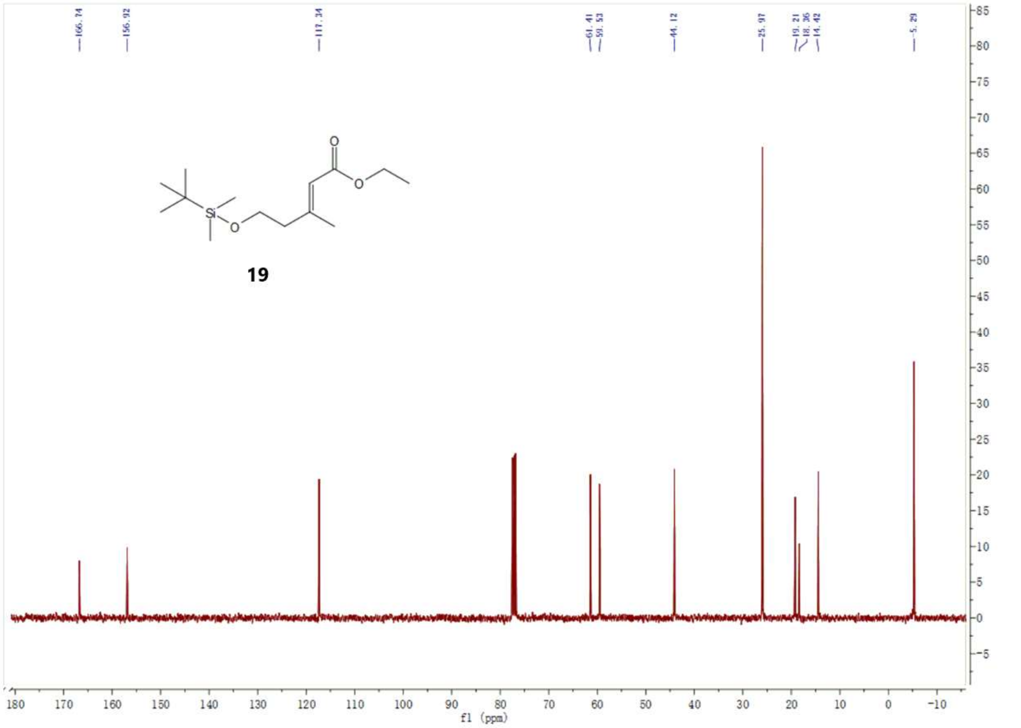

166.74
156.92
117.34
61.41
59.53
44.12
25.97
19.21
18.36
14.42
-5.29
Si
O
O
O
19
f1 (ppm)

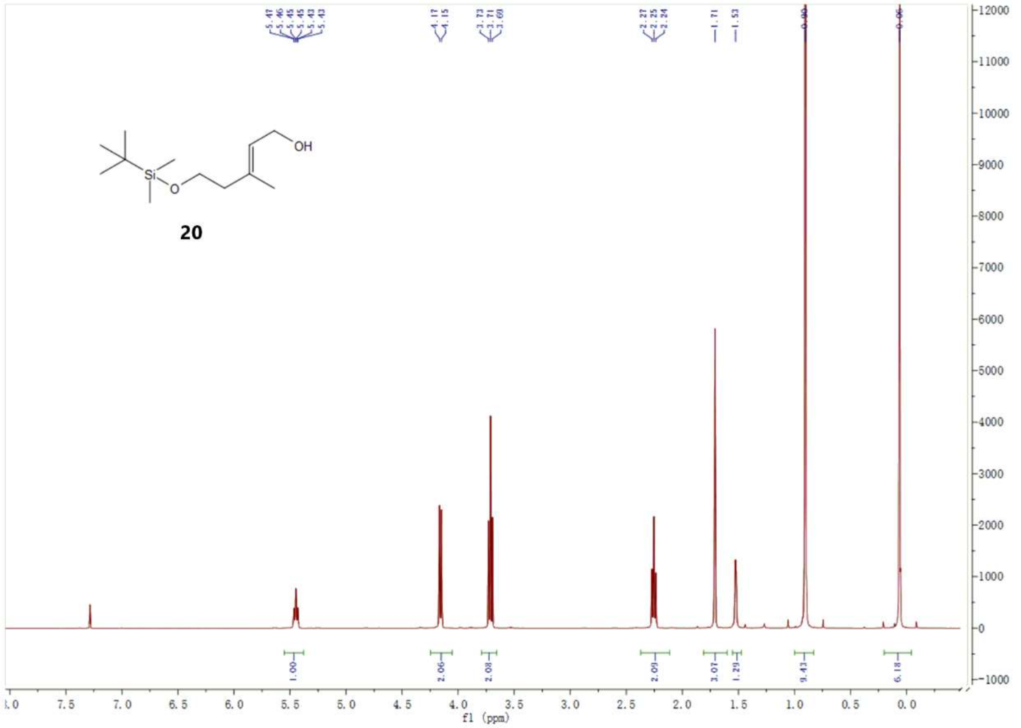
20
f1 (ppm)

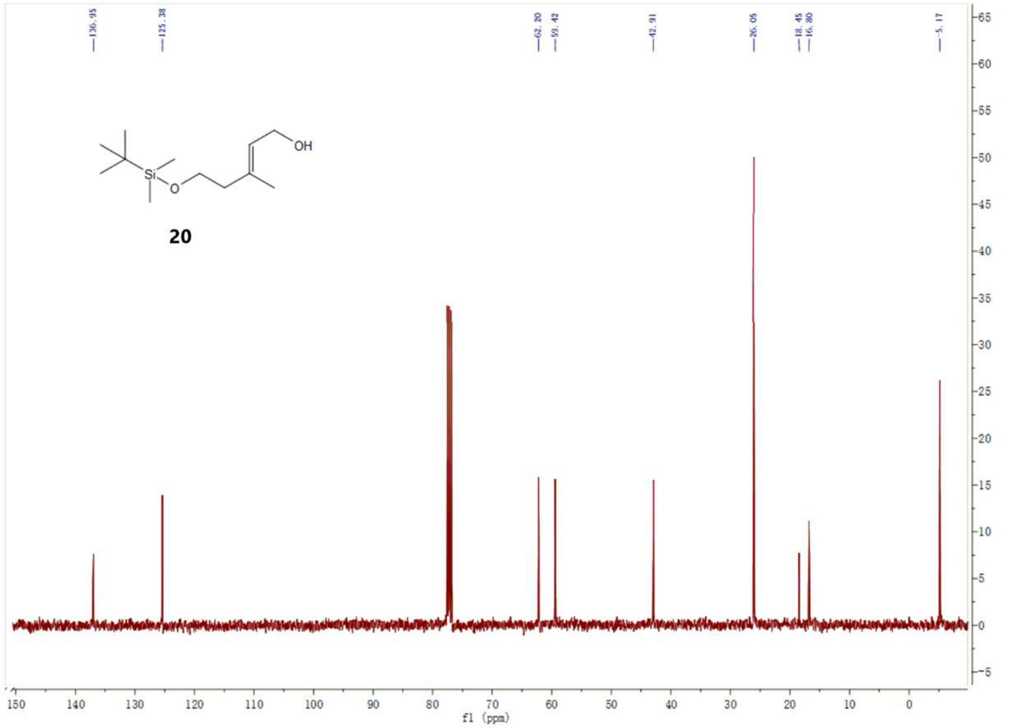
20
f1 (ppm)

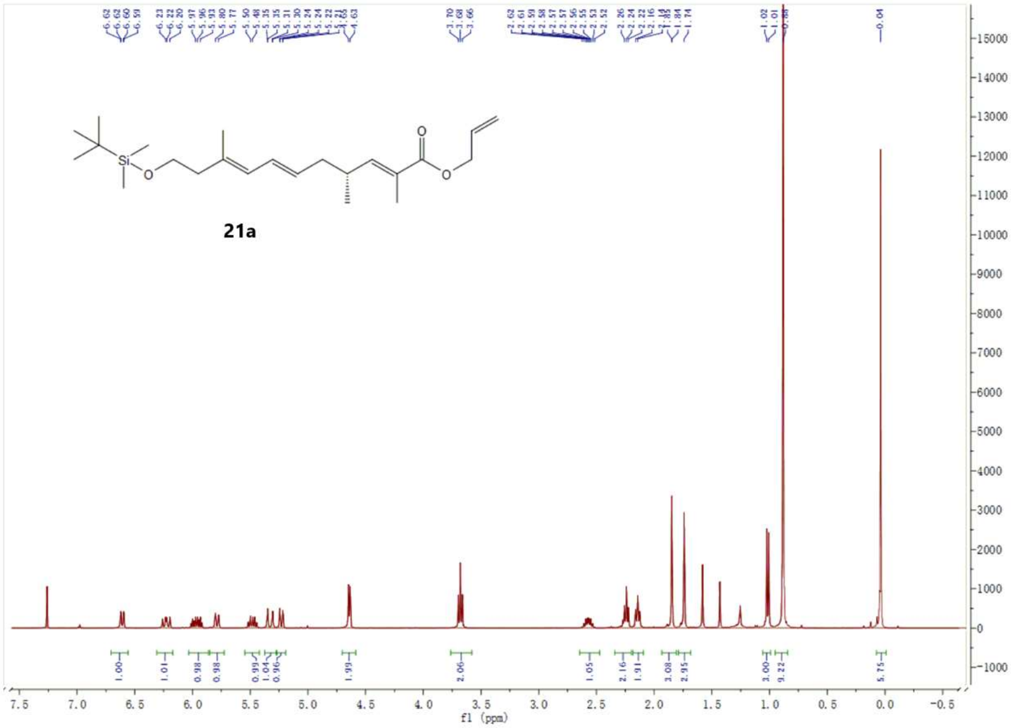
21a
f1 (ppm)

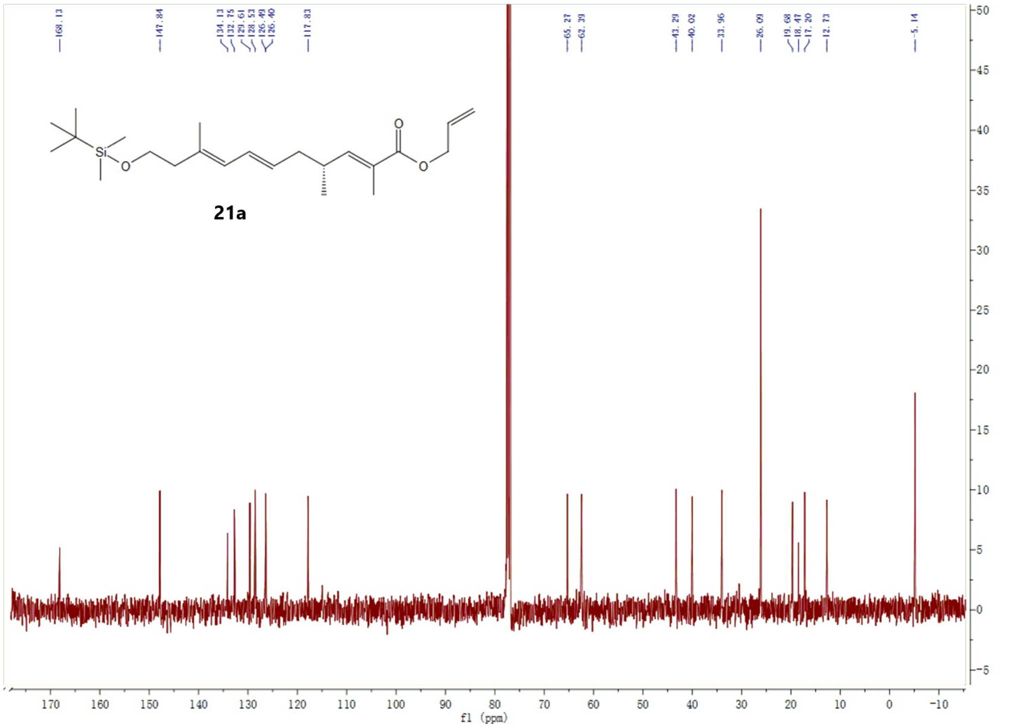
21a
f1 (ppm)

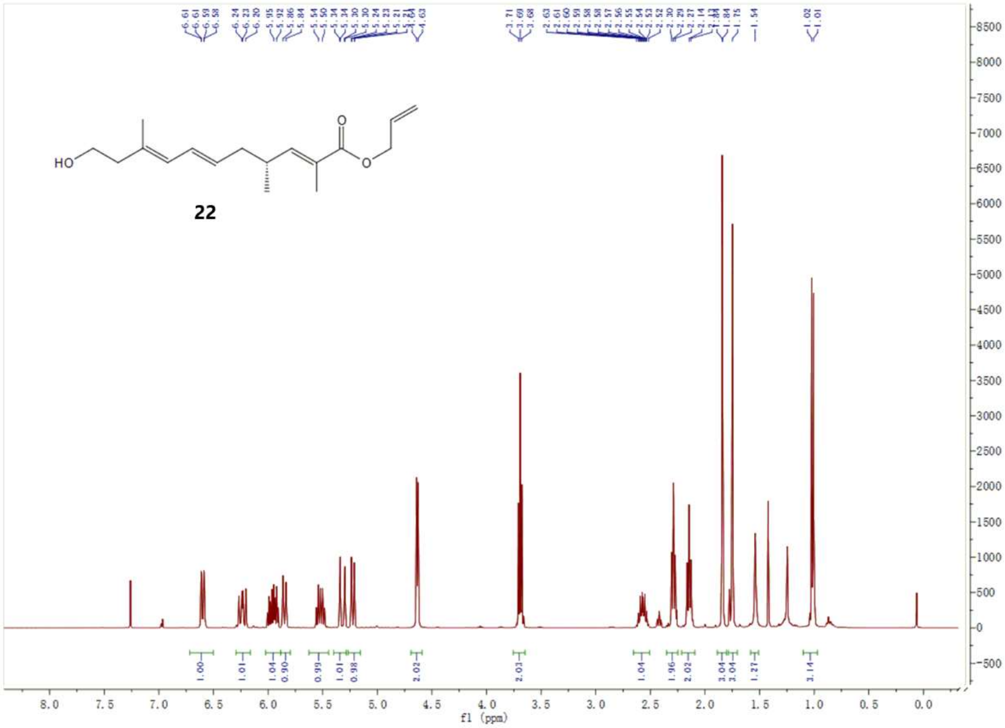
HO
O
O
22
f1 (ppm)

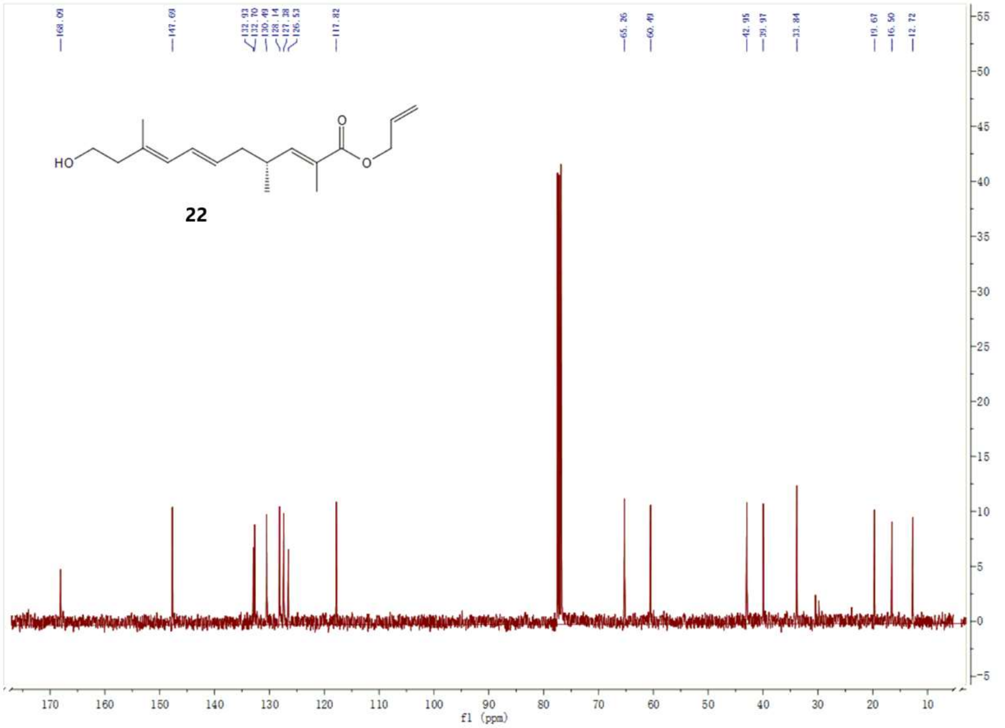
168.09
147.69
117.82
65.26
60.49
42.95
39.97
33.84
19.67
16.50
12.72
HO
O
O
22
f1 (ppm)

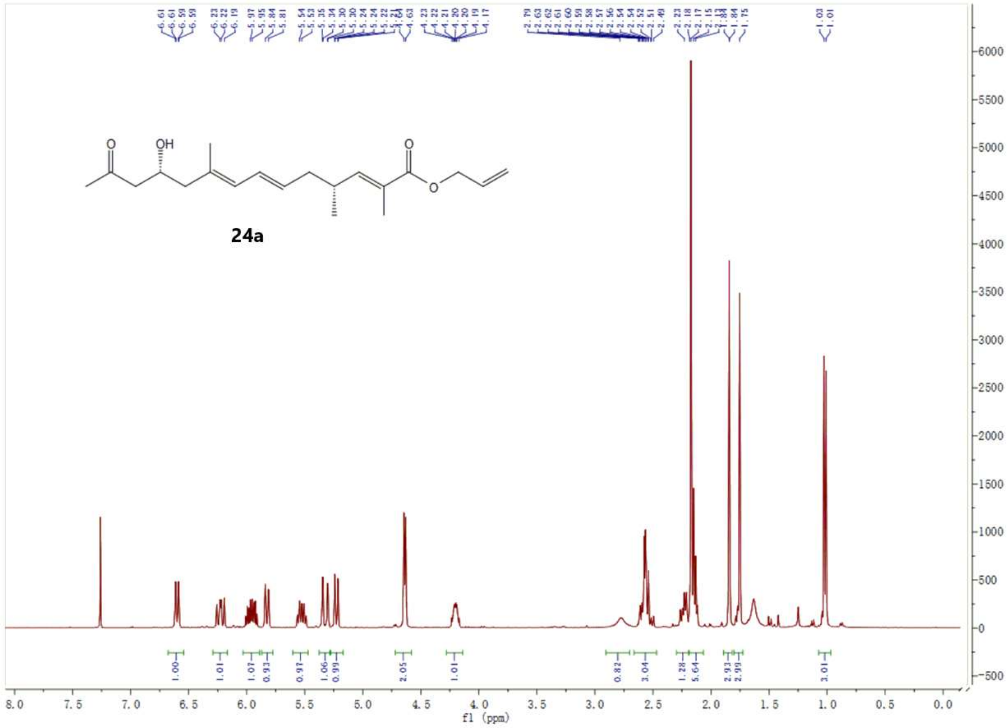
O
OH
O
O
24a
f1 (ppm)

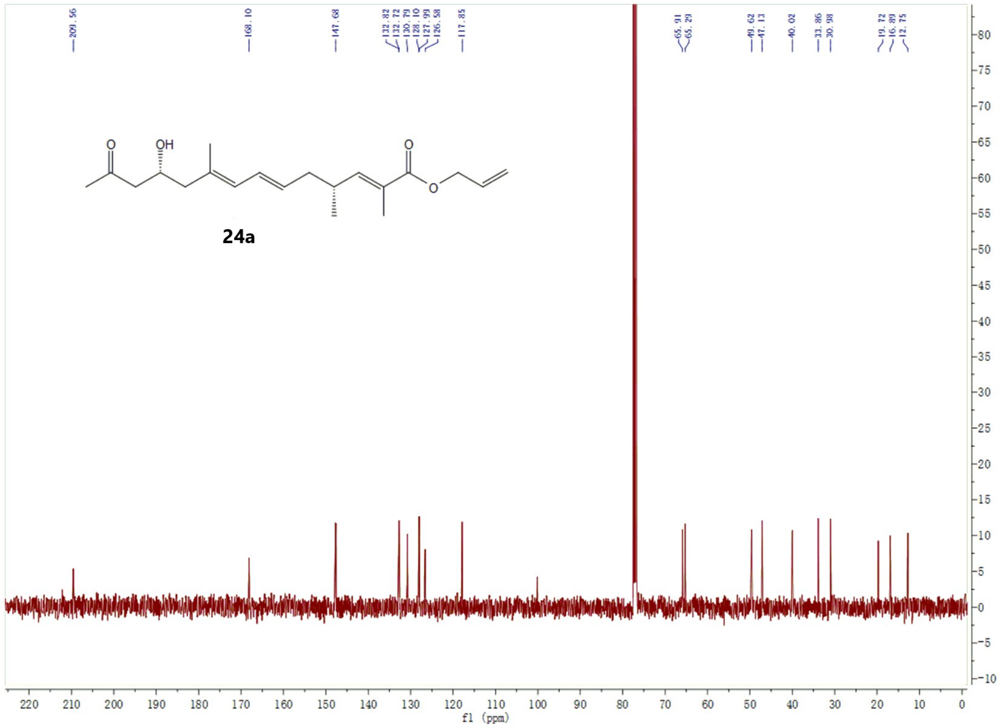
O
OH
O
O
24a
f1 (ppm)

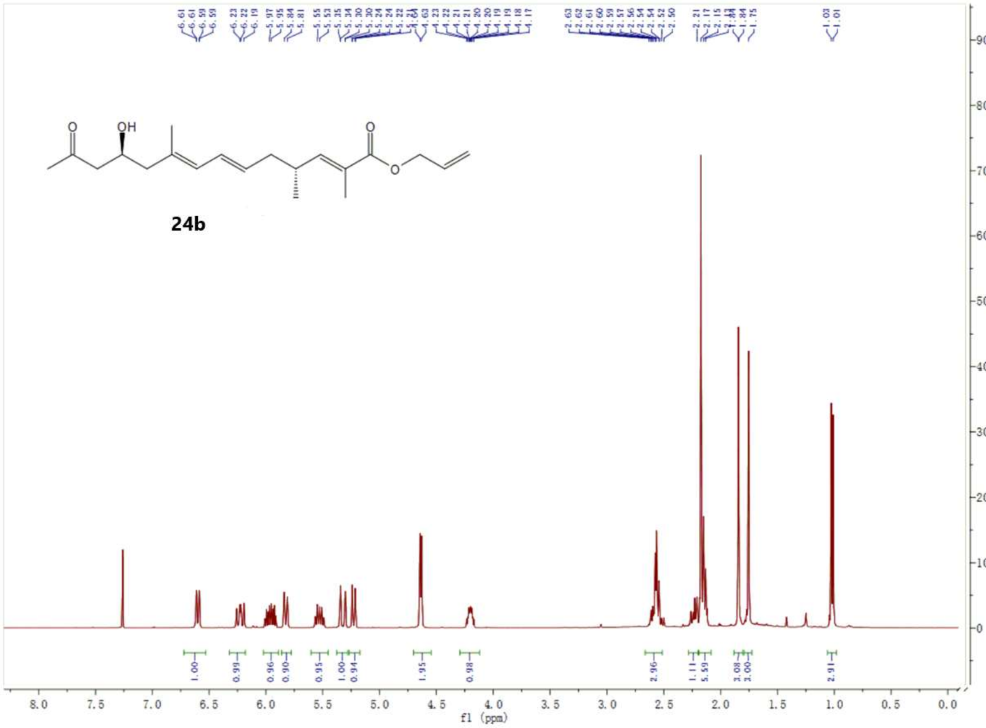

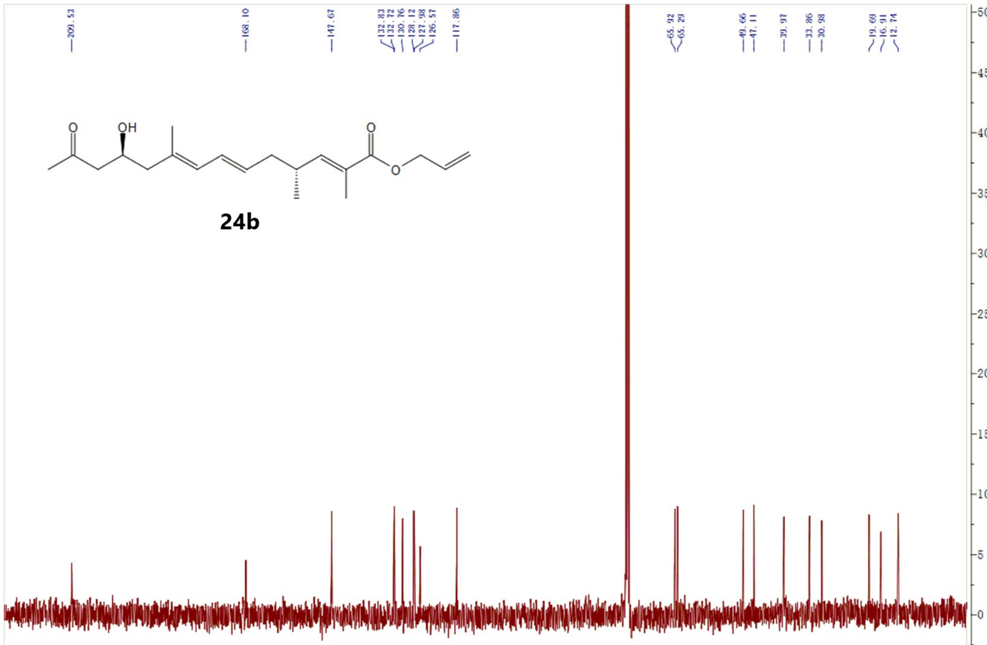

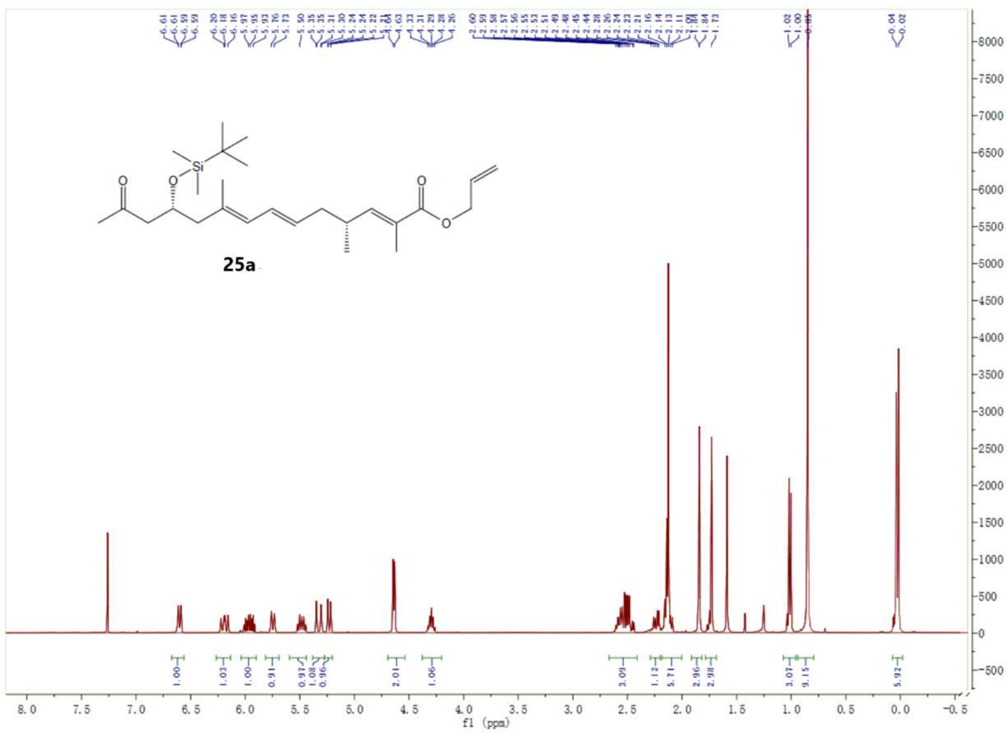
25a
f1 (ppm)

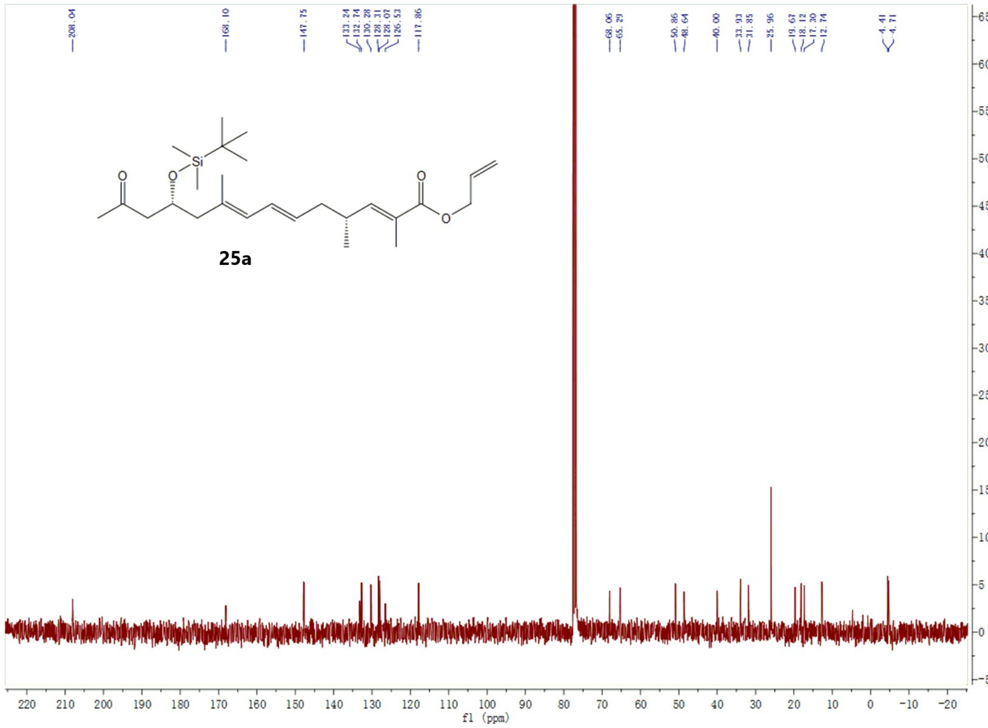
25a
f1 (ppm)

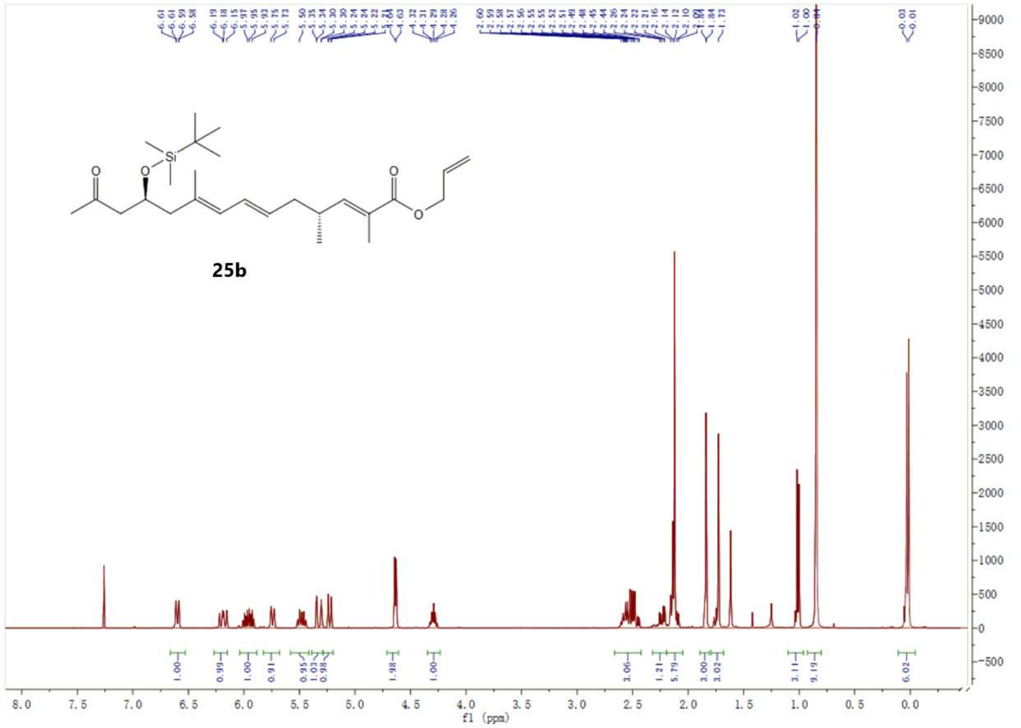
25b
f1 (ppm)

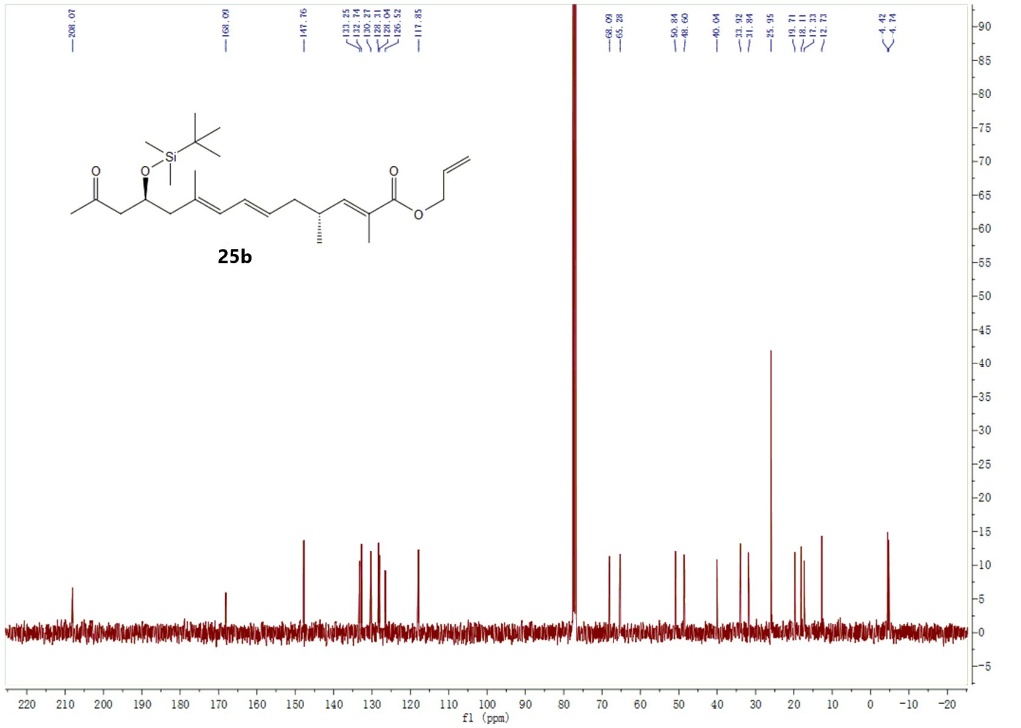
25b
f1 (ppm)

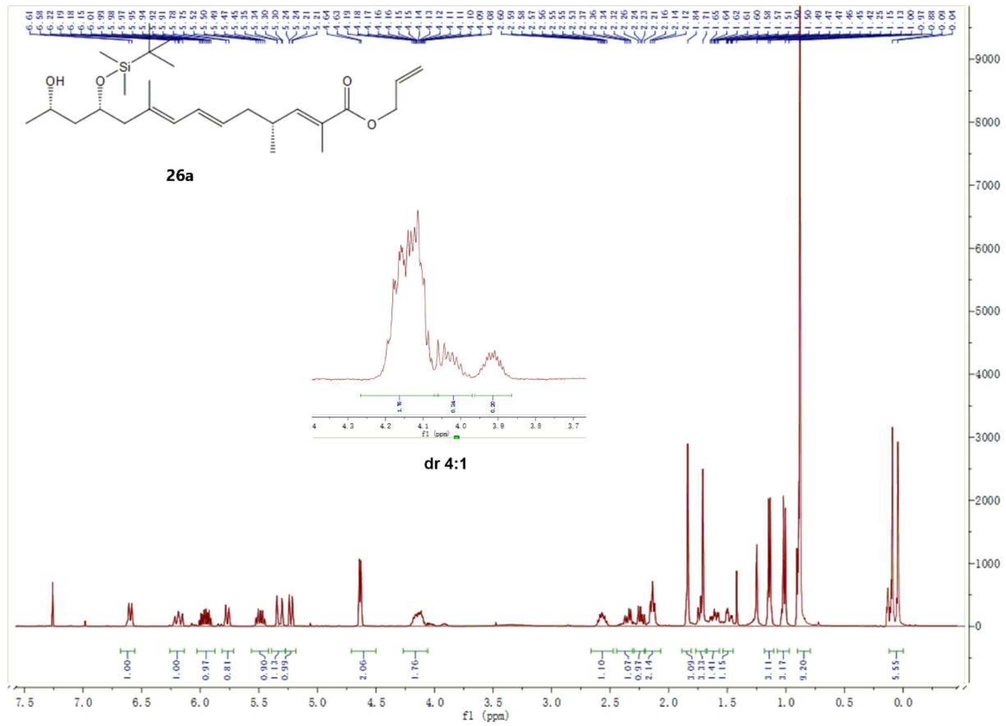
26a
dr 4:1
f1 (ppm)

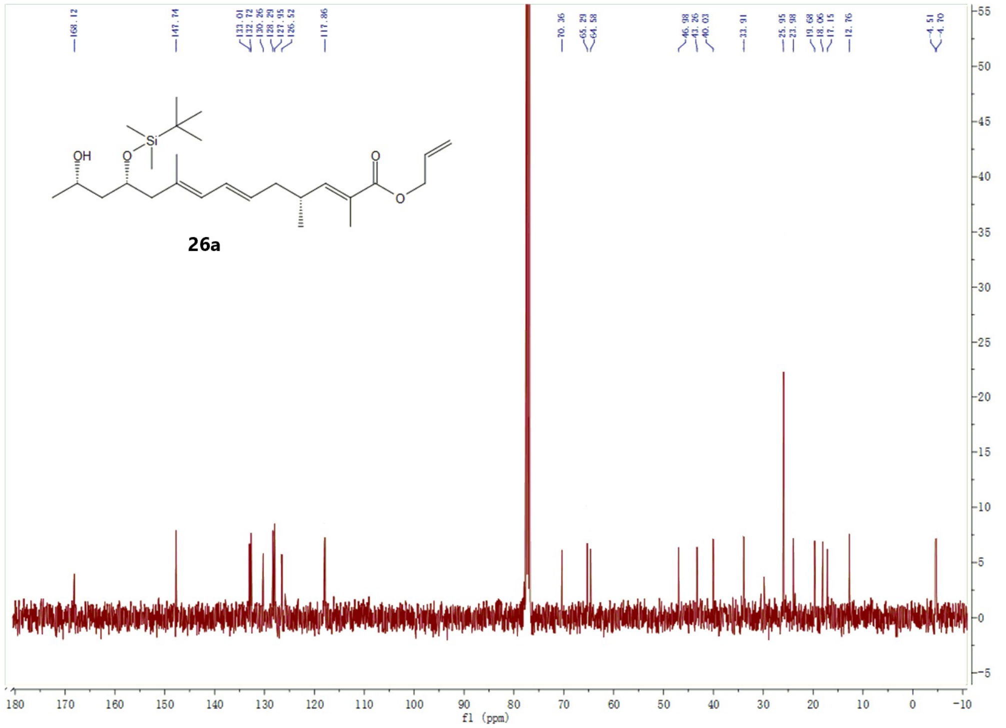
26a
f1 (ppm)

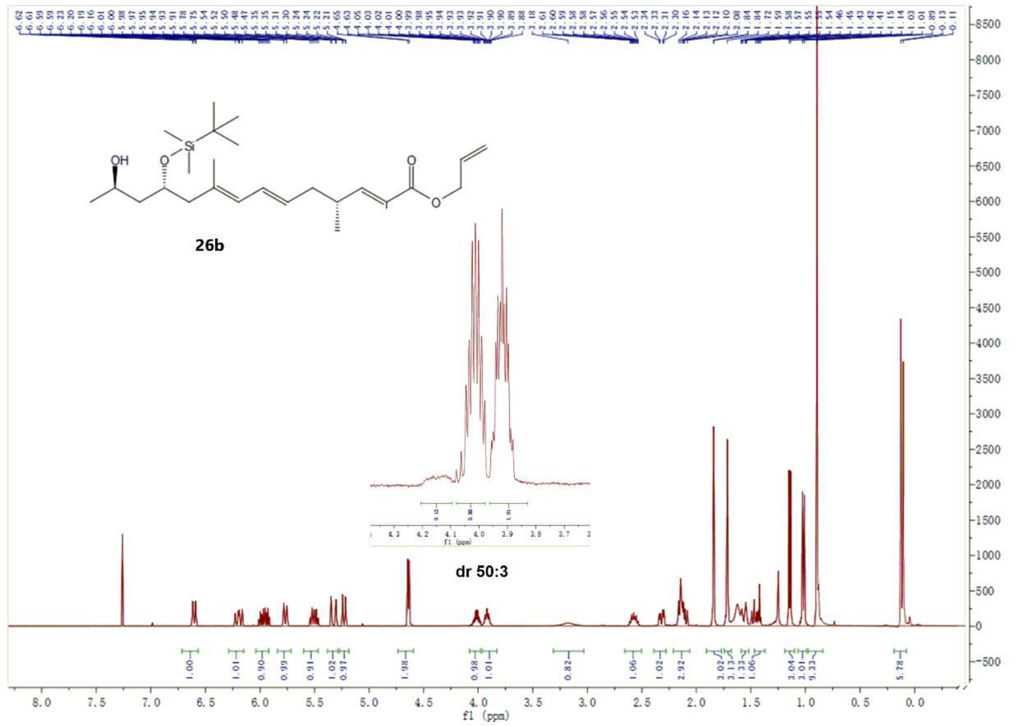

26b
dr 50:3
f1 (ppm)

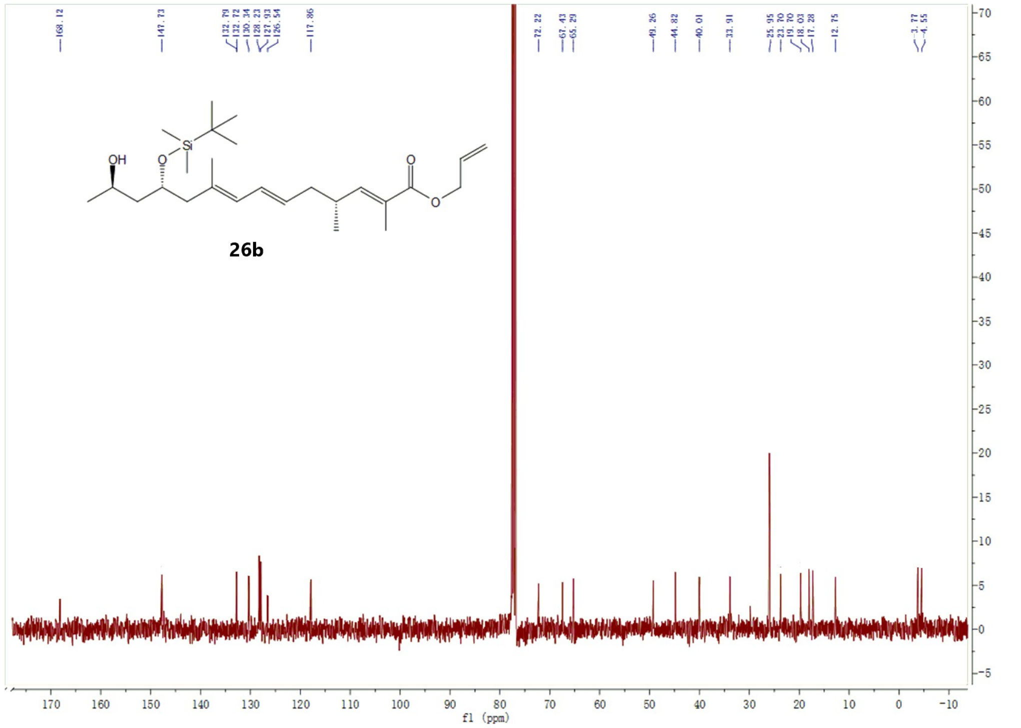

26b
f1 (ppm)

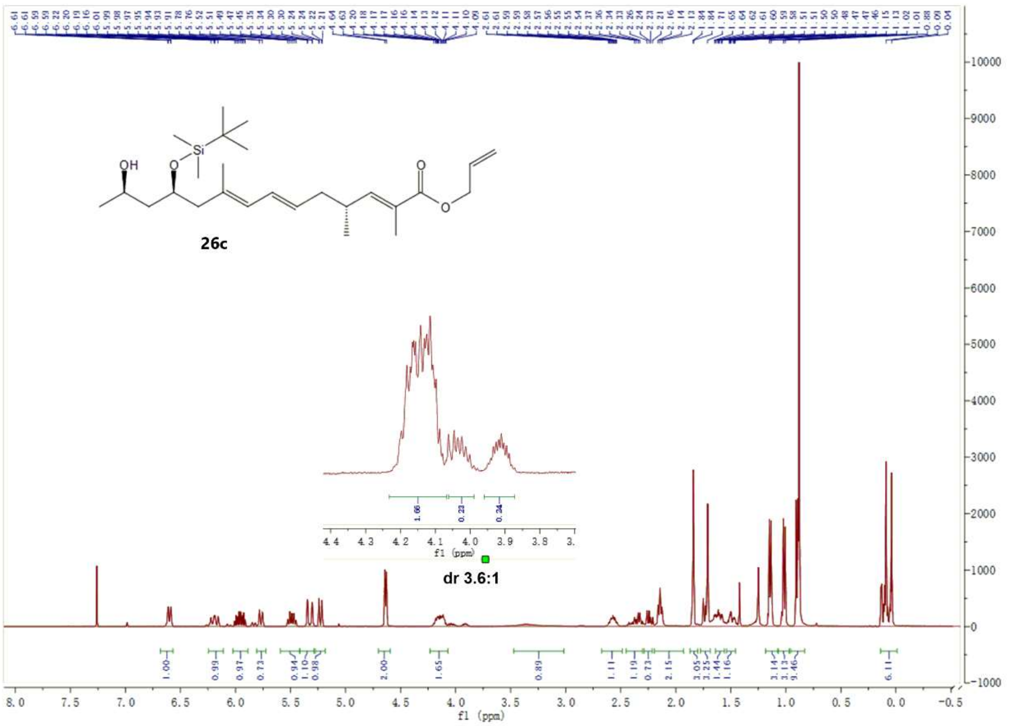
26c
dr 3.6:1
f1 (ppm)

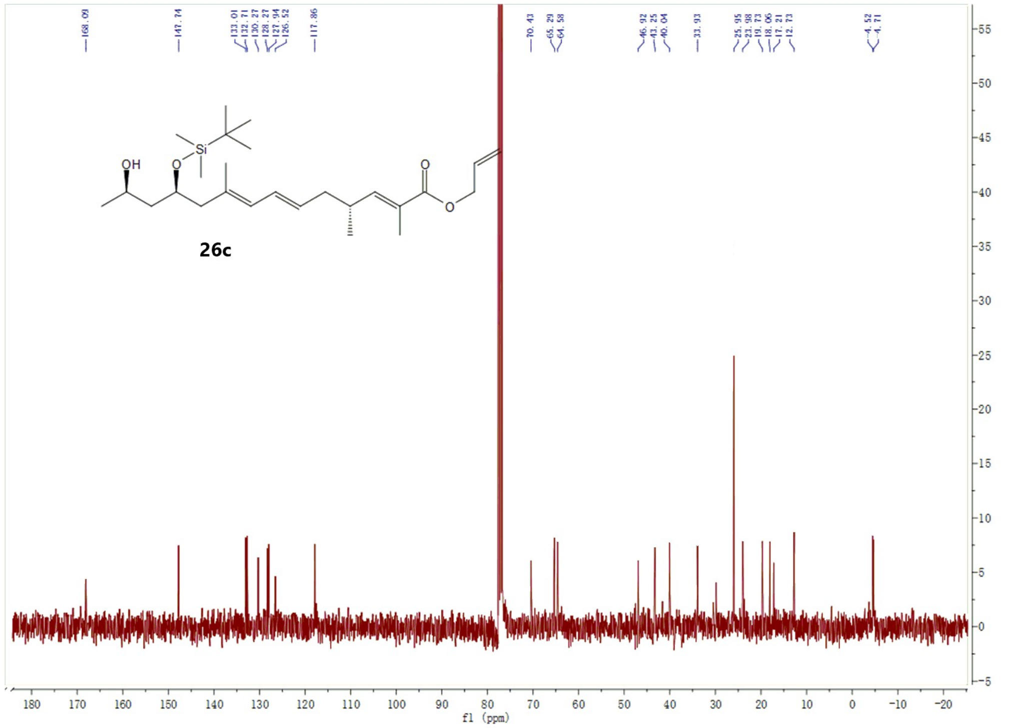
26c
f1 (ppm)

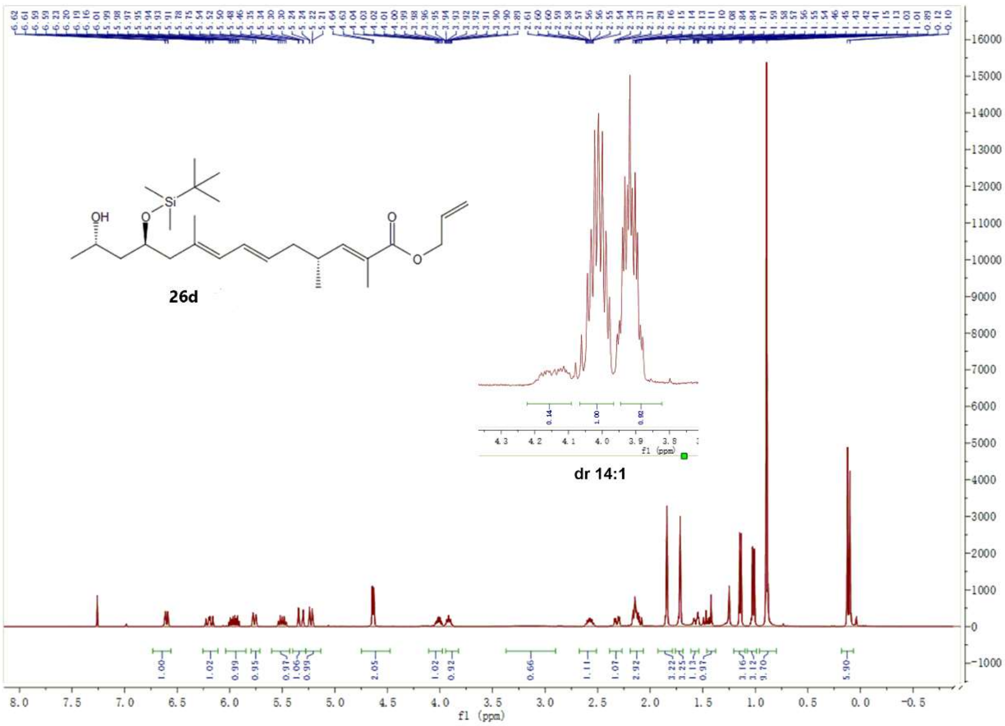
OH
O
Si
26d
dr 14:1
f1 (ppm)

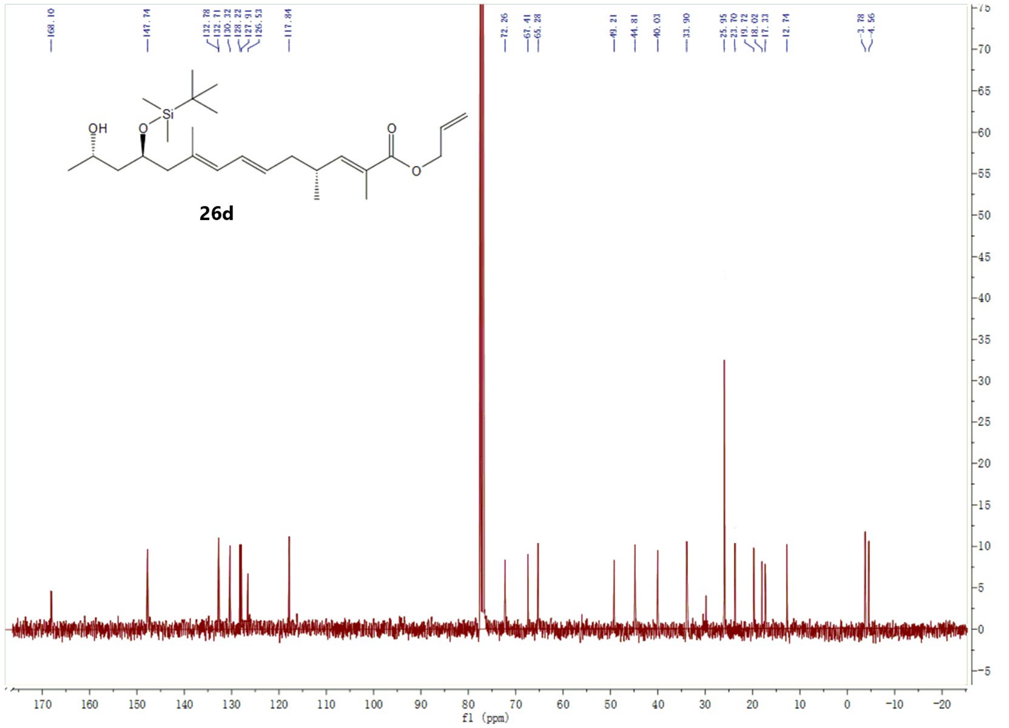
OH
O
Si
26d
f1 (ppm)

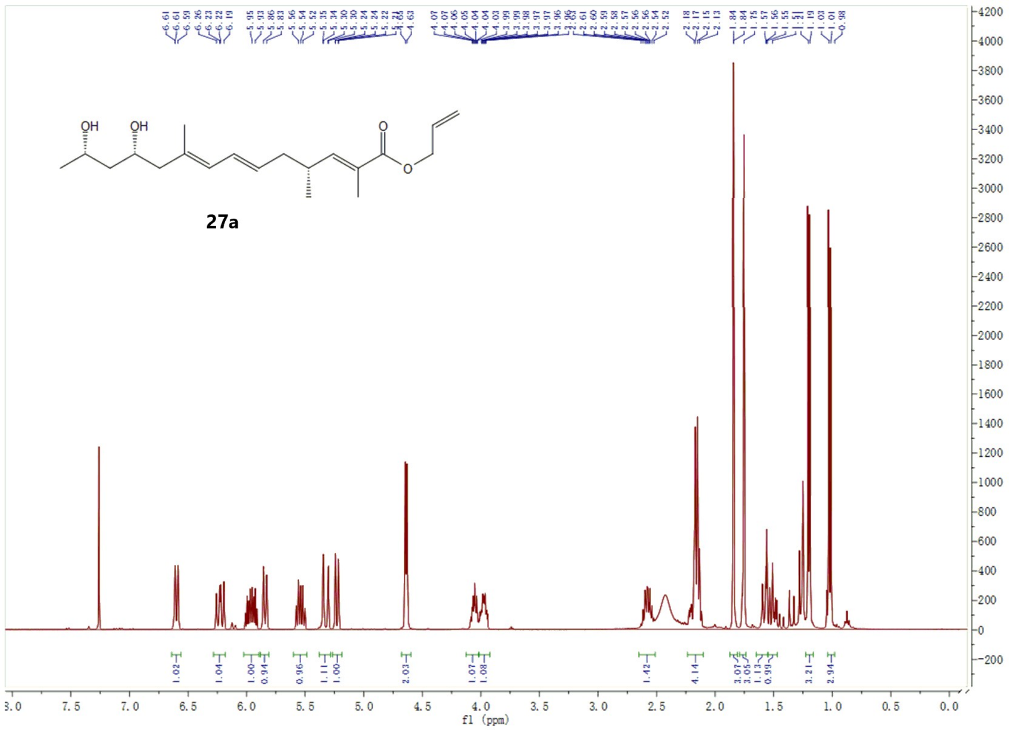
OH
OH
O
O
27a
f1 (ppm)

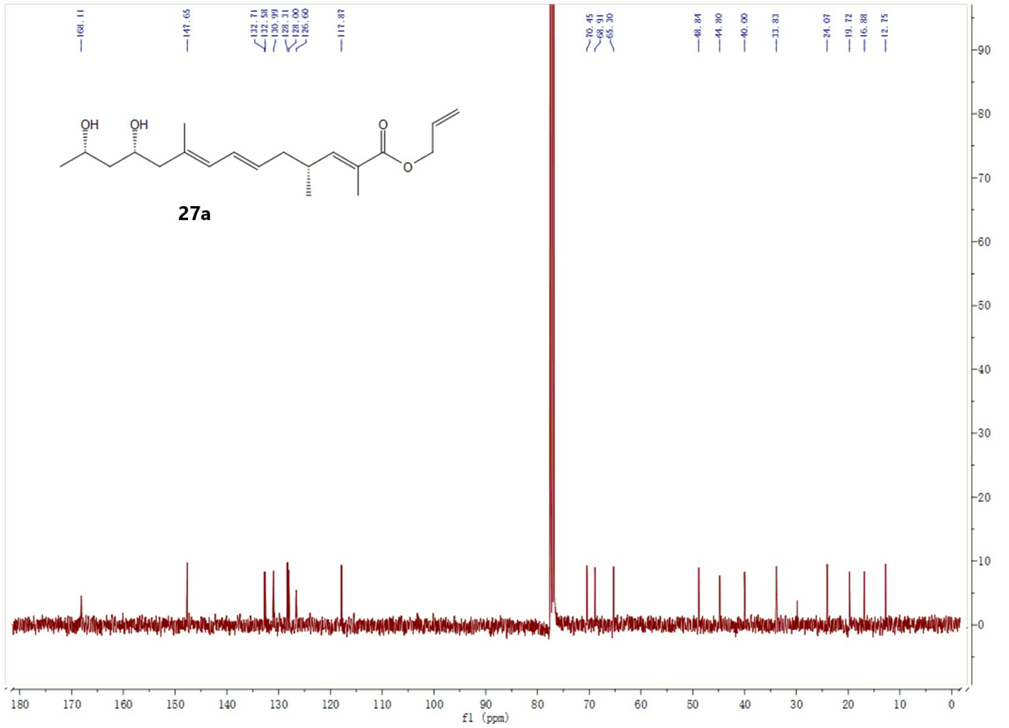
168.11
147.65
132.71
132.58
130.99
128.31
128.00
126.60
117.87
70.45
68.91
65.30
48.84
44.80
40.00
33.83
24.07
19.72
16.88
12.75
OH
OH
O
O
27a
f1 (ppm)

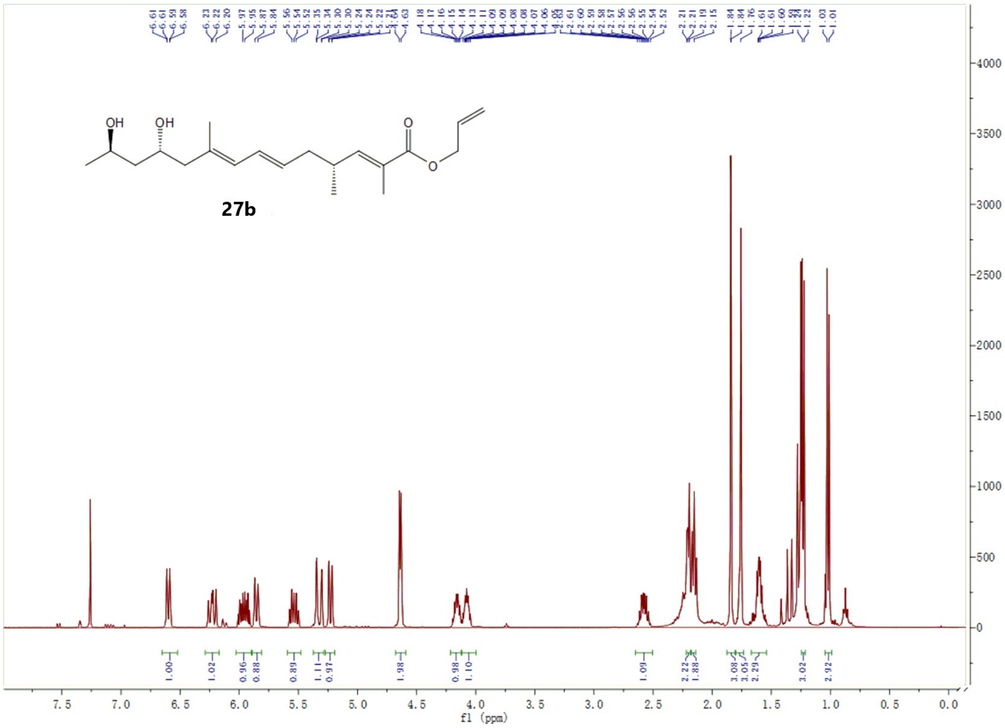

OH
OH
O
O
27b
f1 (ppm)

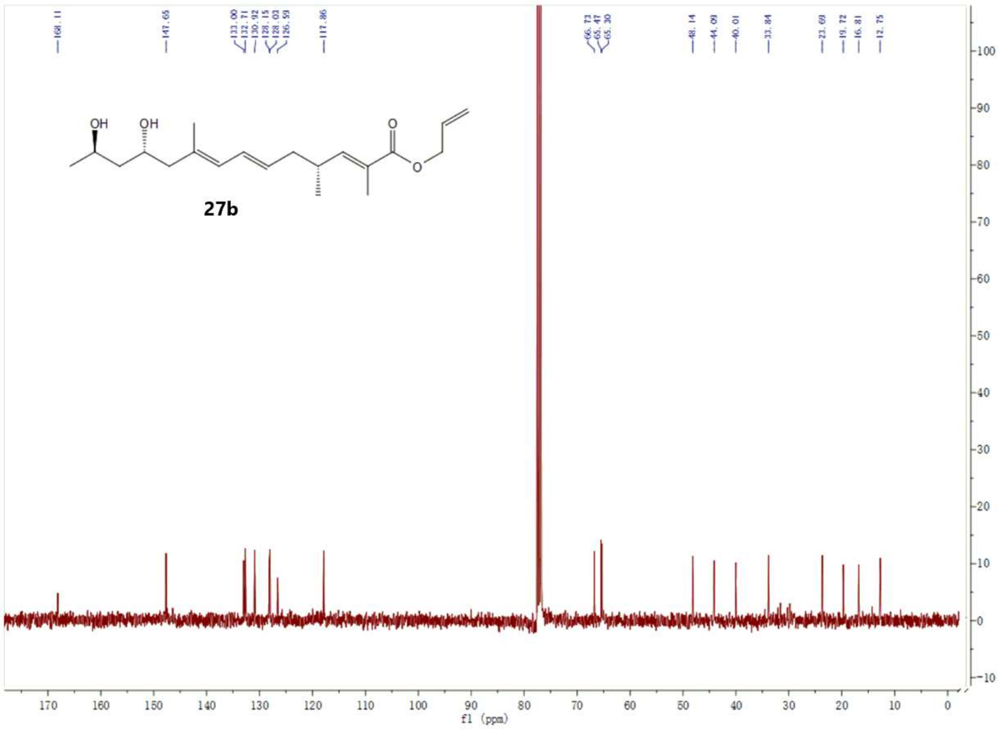

168.11
147.65
133.00
132.71
130.92
128.15
128.03
126.59
117.86
66.72
65.47
65.30
48.14
44.09
40.01
33.84
23.69
19.72
16.81
12.75
OH
OH
O
O
27b
f1 (ppm)

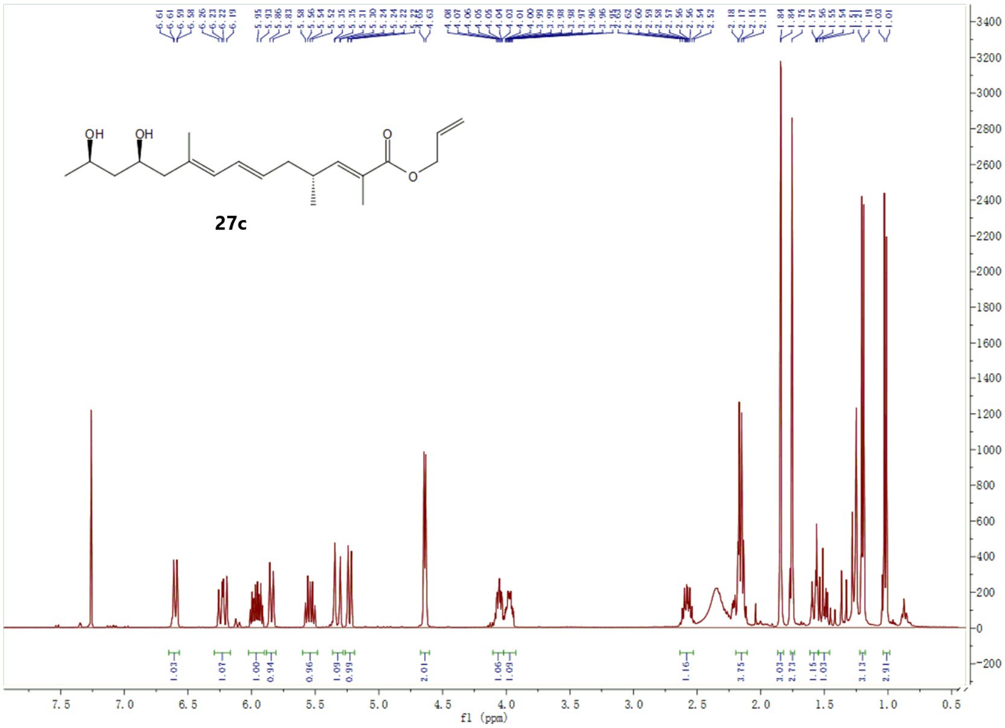
OH
OH
O
O
27c
f1 (ppm)

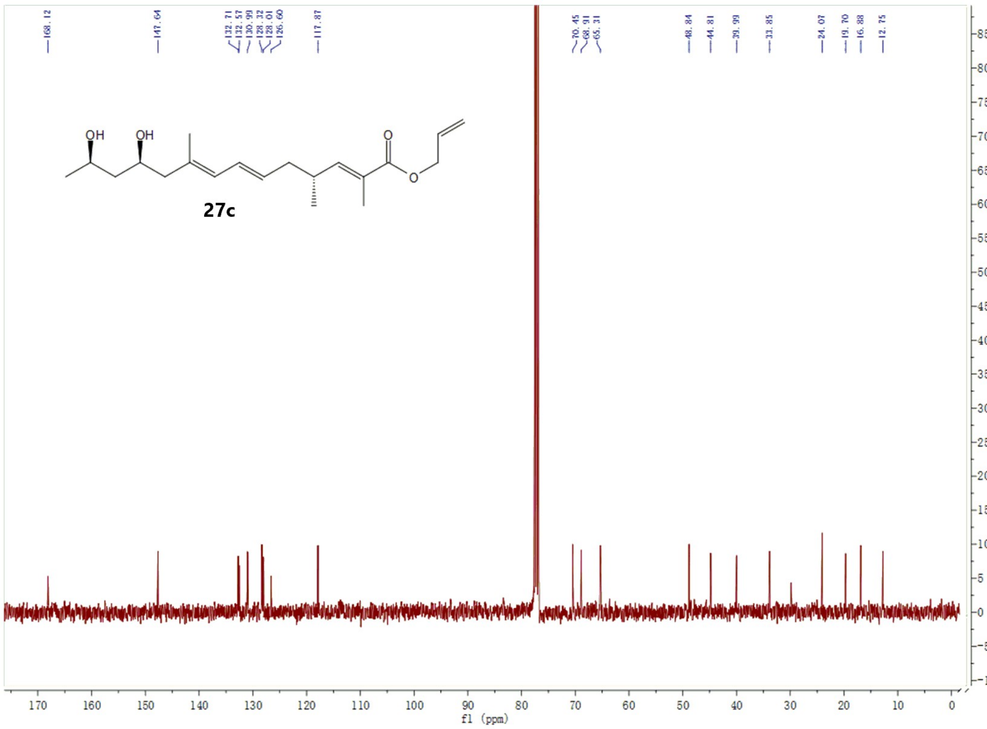
168.12
147.64
132.71
132.57
130.99
128.32
128.01
126.60
117.87
70.45
68.91
65.31
48.84
44.81
39.99
33.85
24.07
19.70
16.88
12.75
OH
OH
O
O
27c
f1 (ppm)

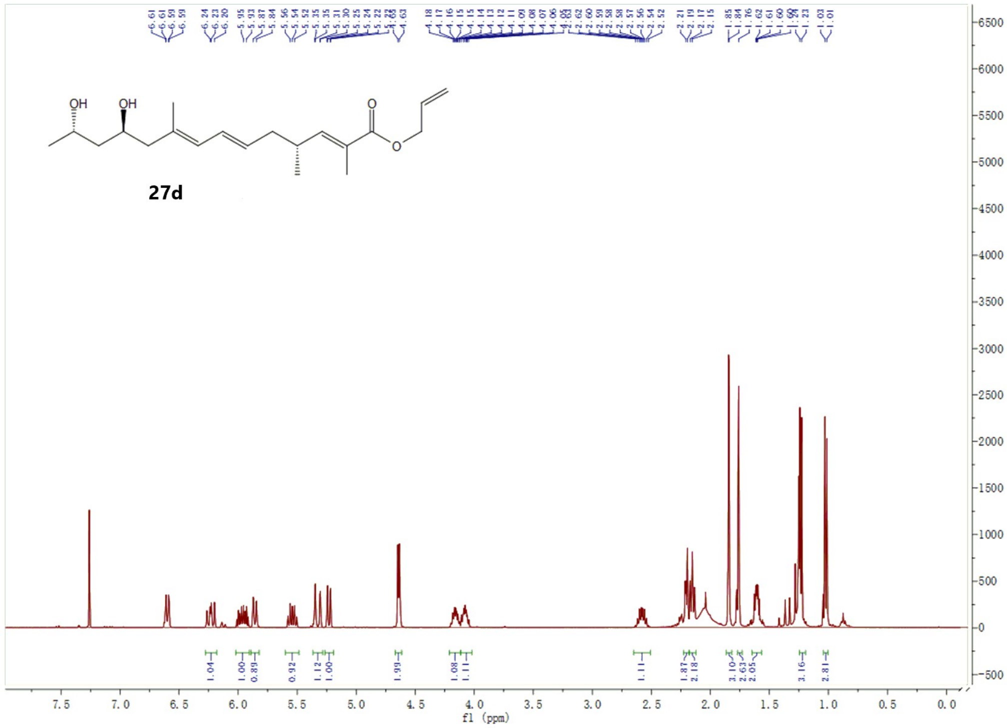

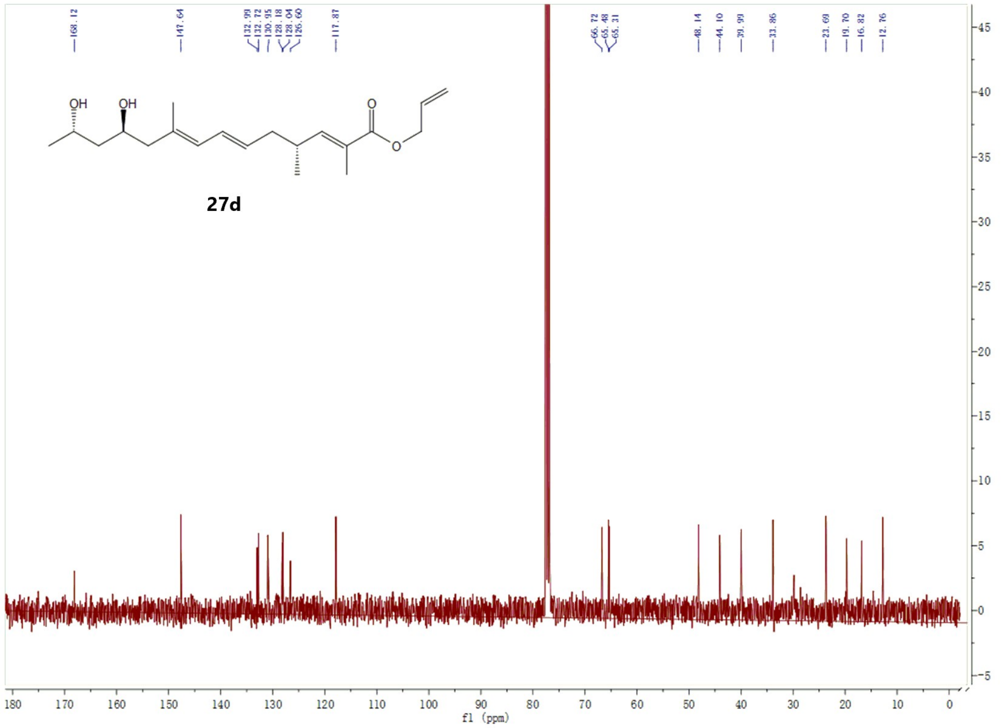

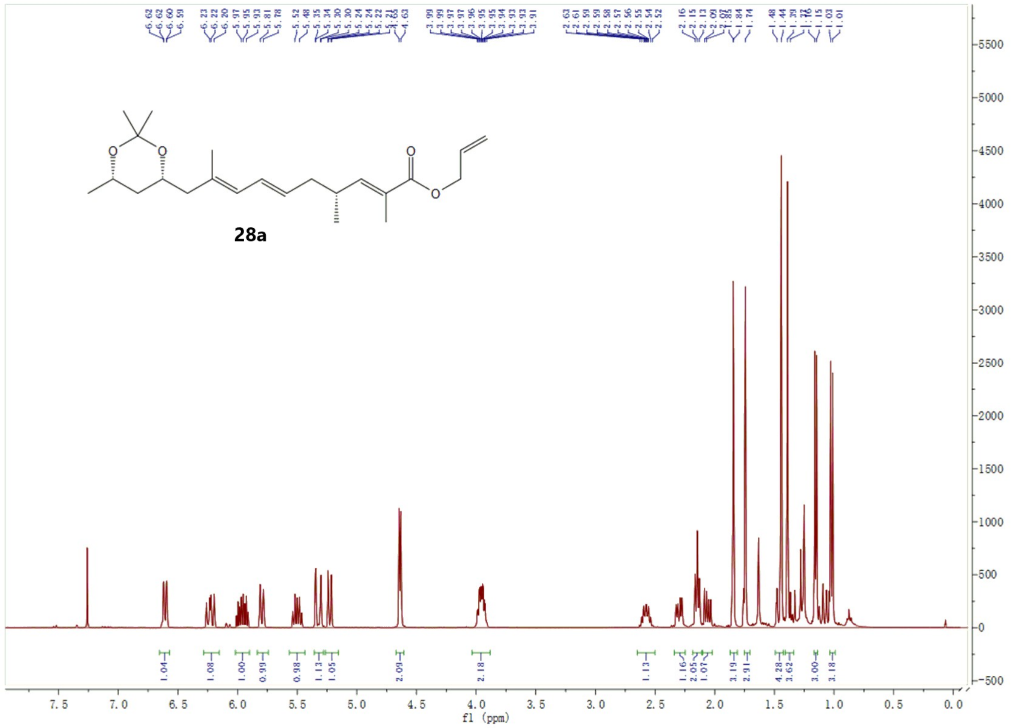
28a
f1 (ppm)

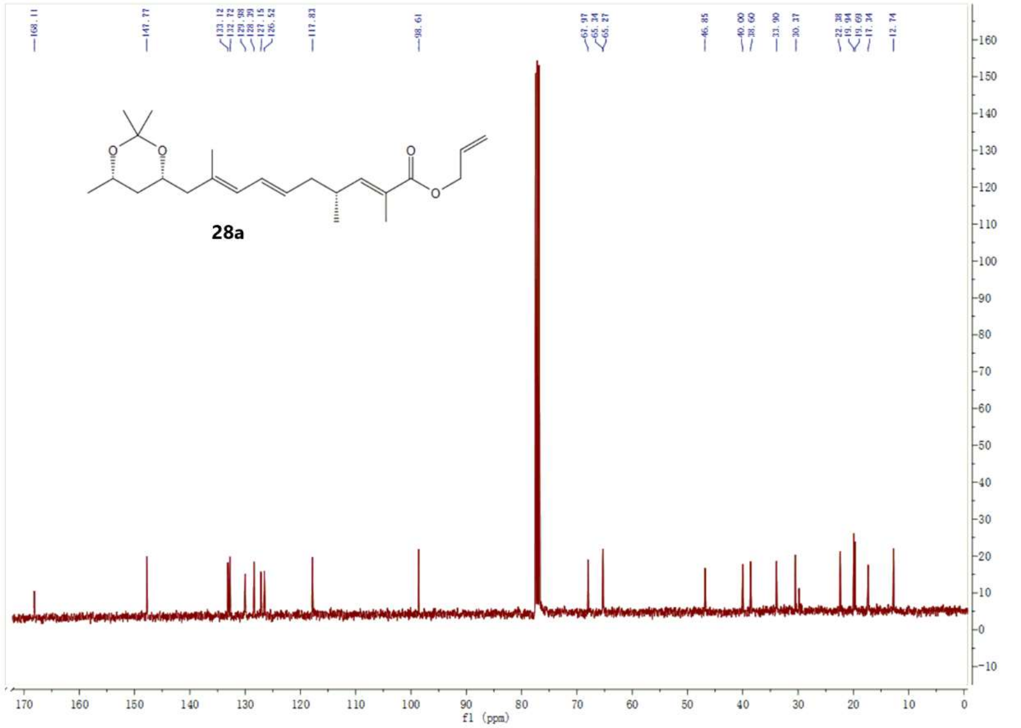
28a
f1 (ppm)

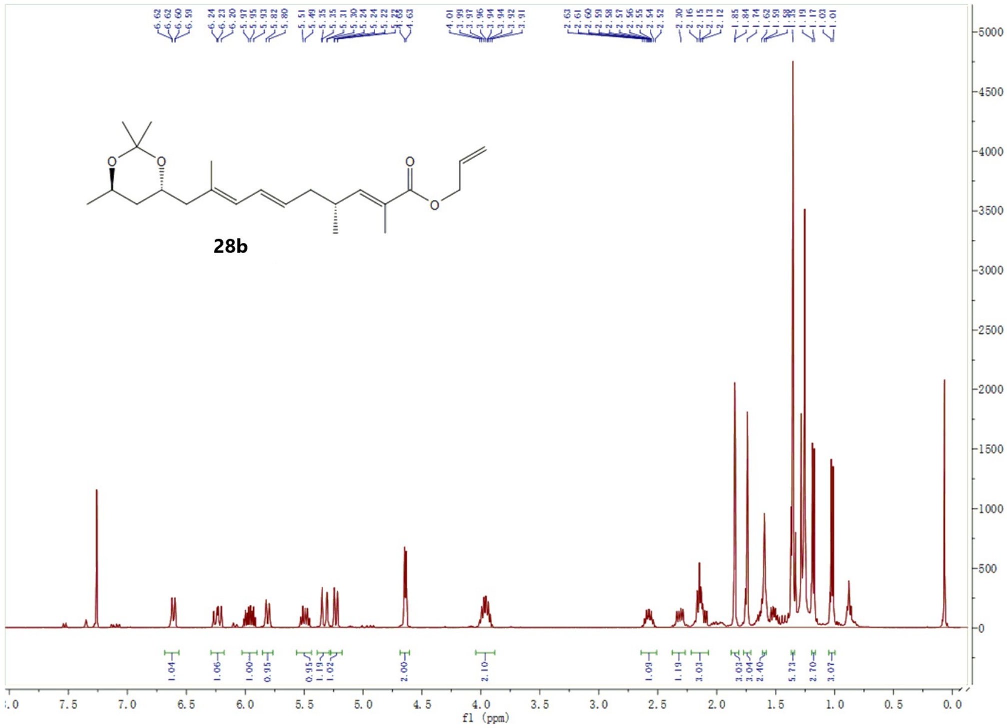

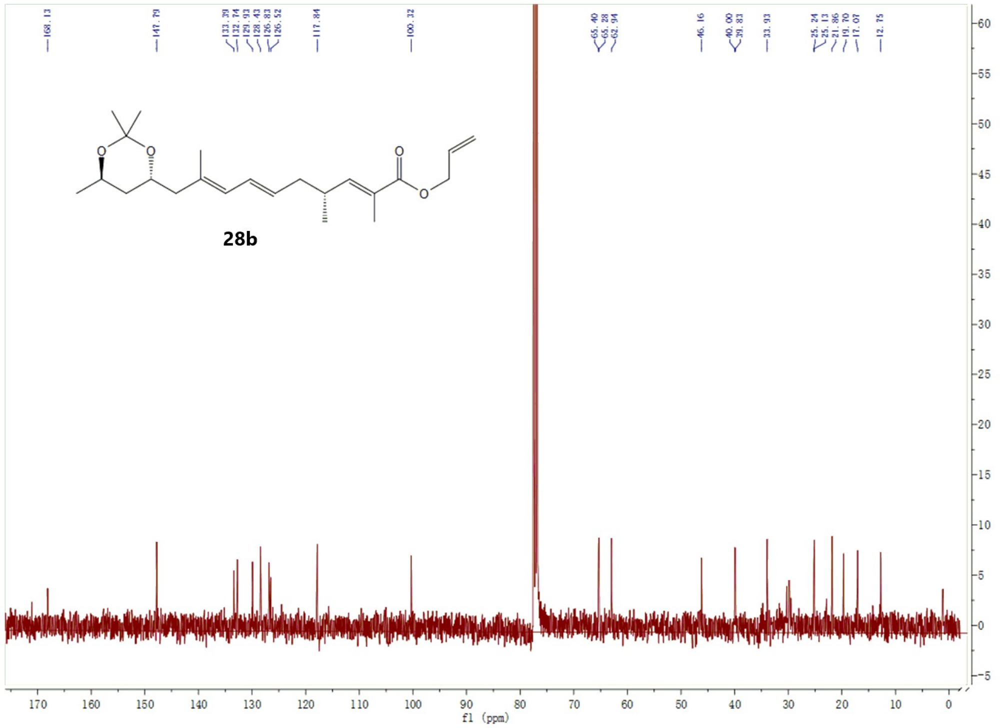

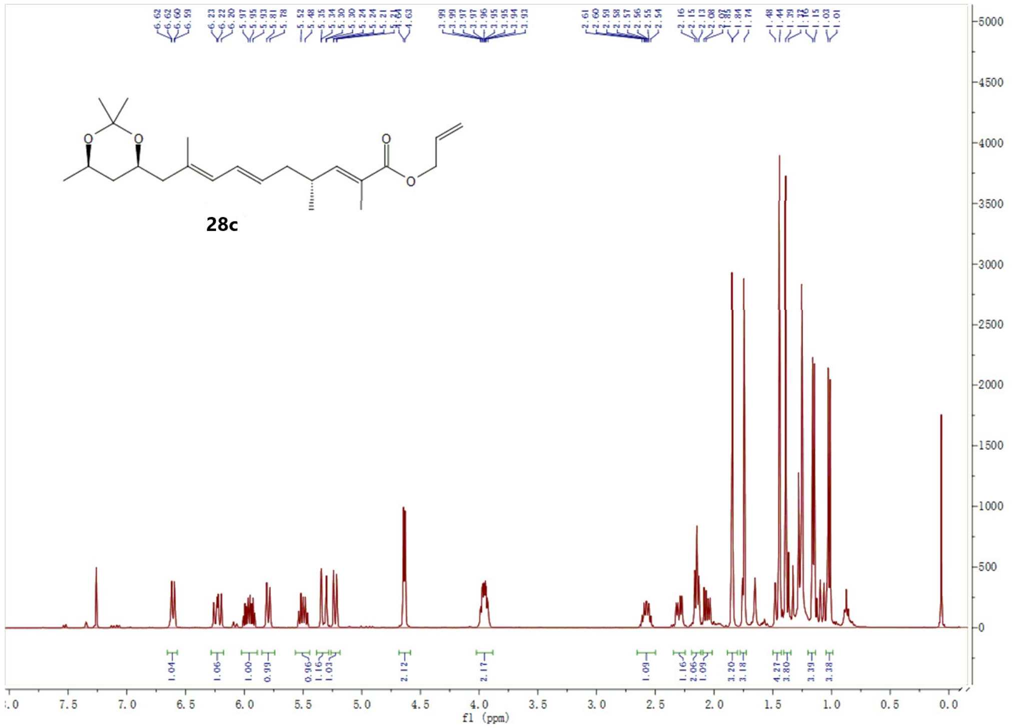
28c
f1 (ppm)

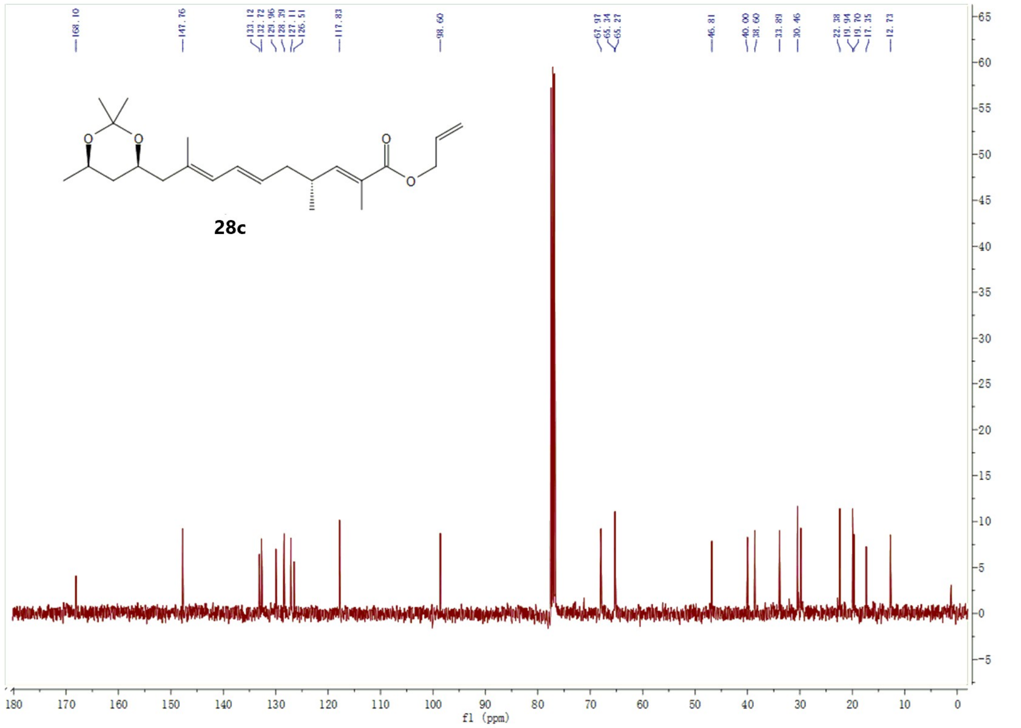
28c
f1 (ppm)

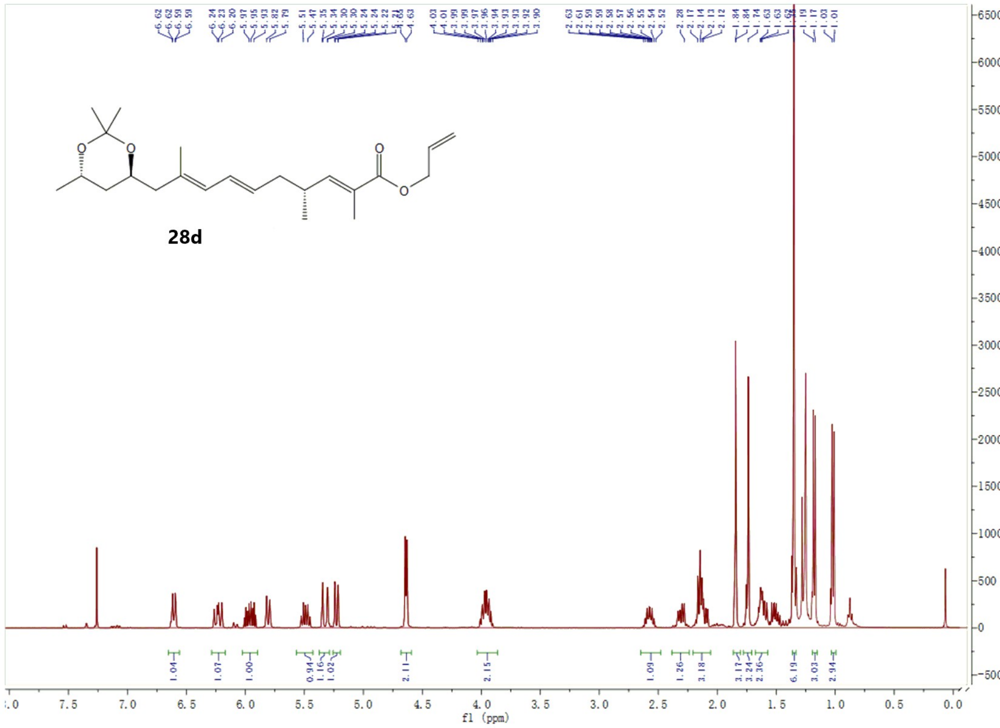
28d
f1 (ppm)

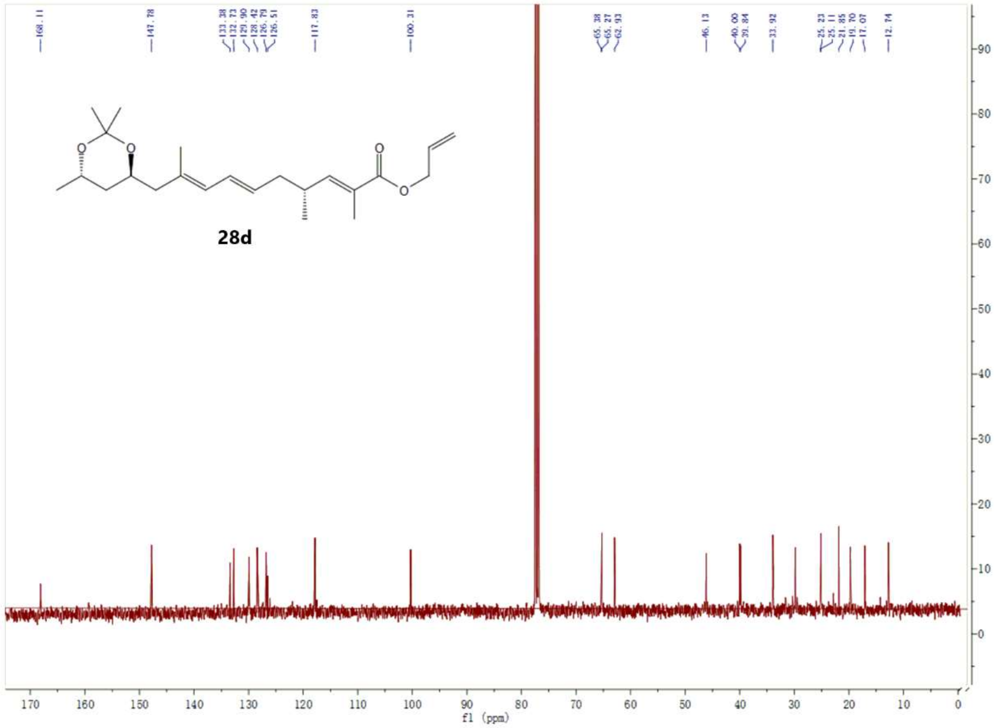
168.11
147.78
133.38
132.73
129.90
128.42
126.79
126.51
117.83
100.31
65.38
65.27
62.93
46.13
40.00
39.84
33.92
25.23
25.11
21.85
19.70
17.07
12.74
28d
f1 (ppm)

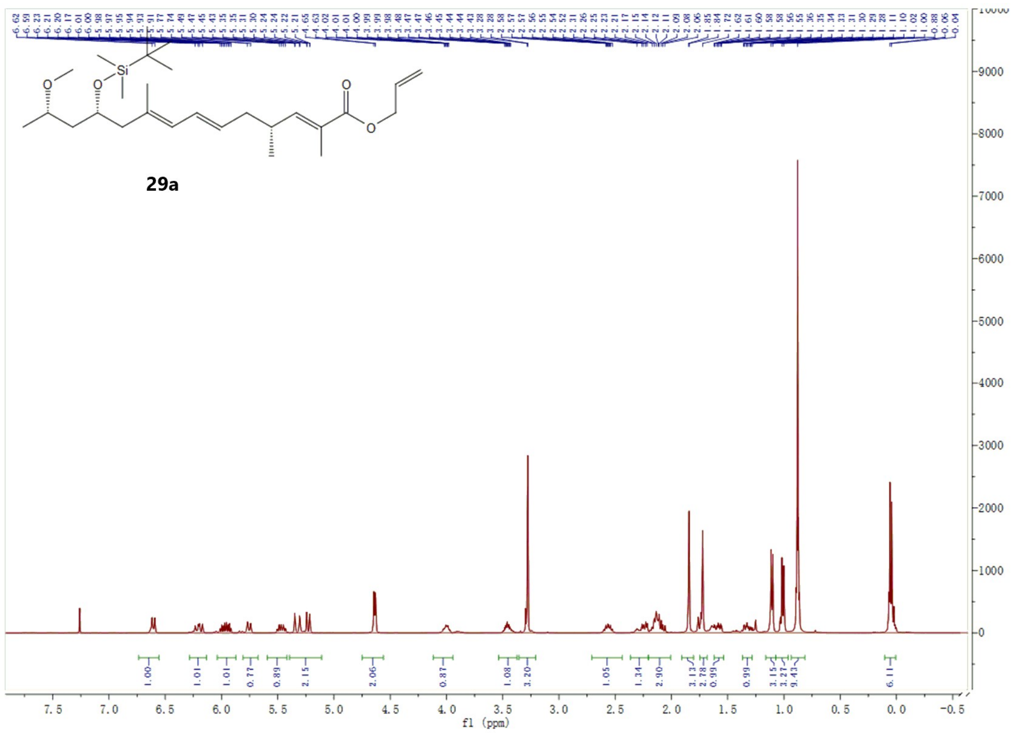

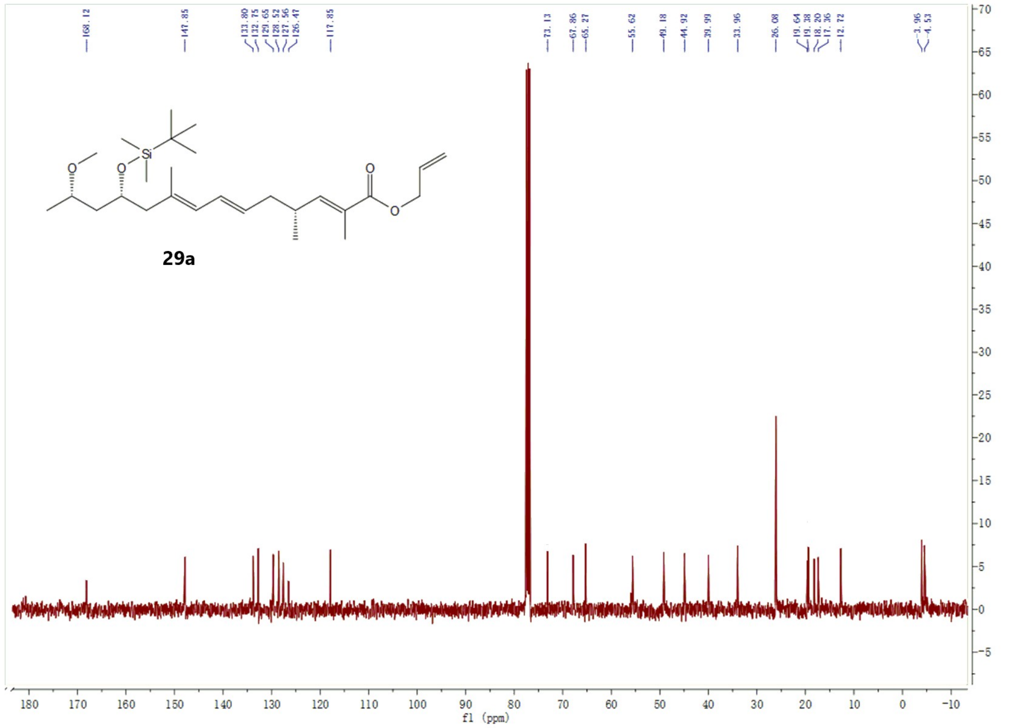

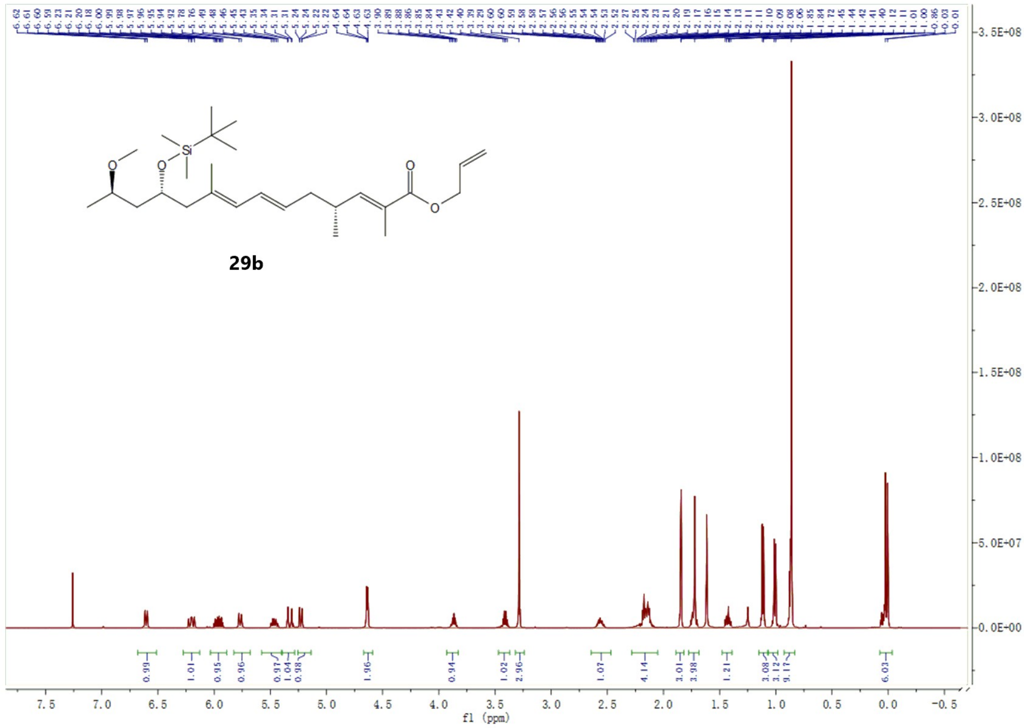

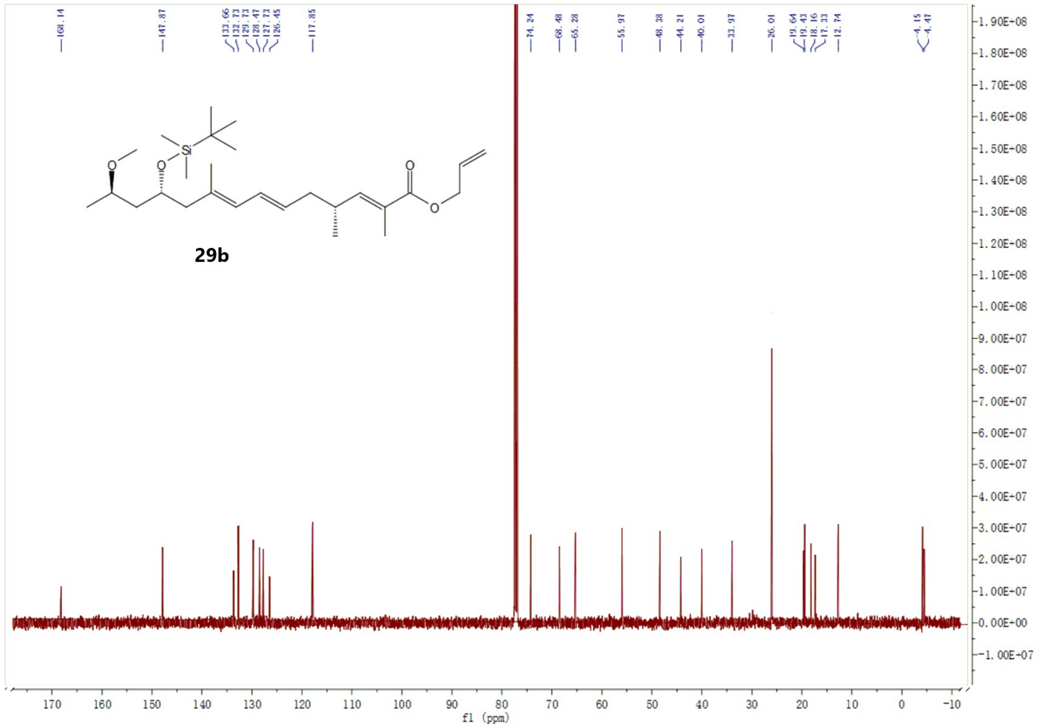

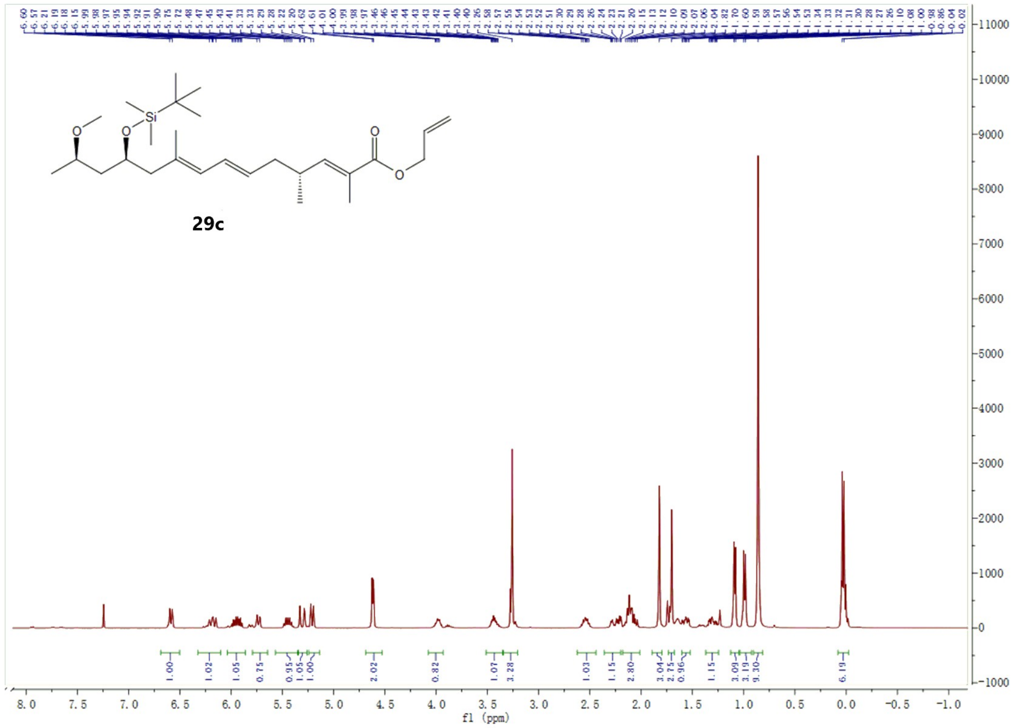
29c
f1 (ppm)

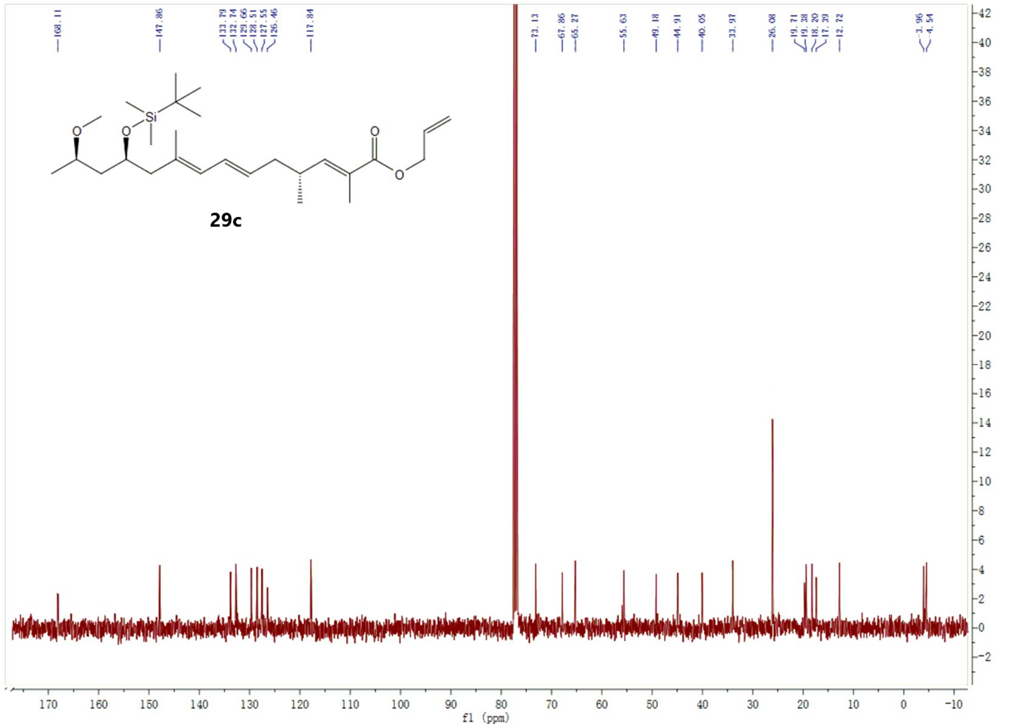
29c
168.11
147.86
133.79
132.74
129.66
128.51
127.55
126.46
117.84
73.13
67.86
65.27
55.63
49.18
44.91
40.05
33.97
26.08
19.71
19.38
18.20
17.39
12.72
-3.96
-4.54
f1 (ppm)

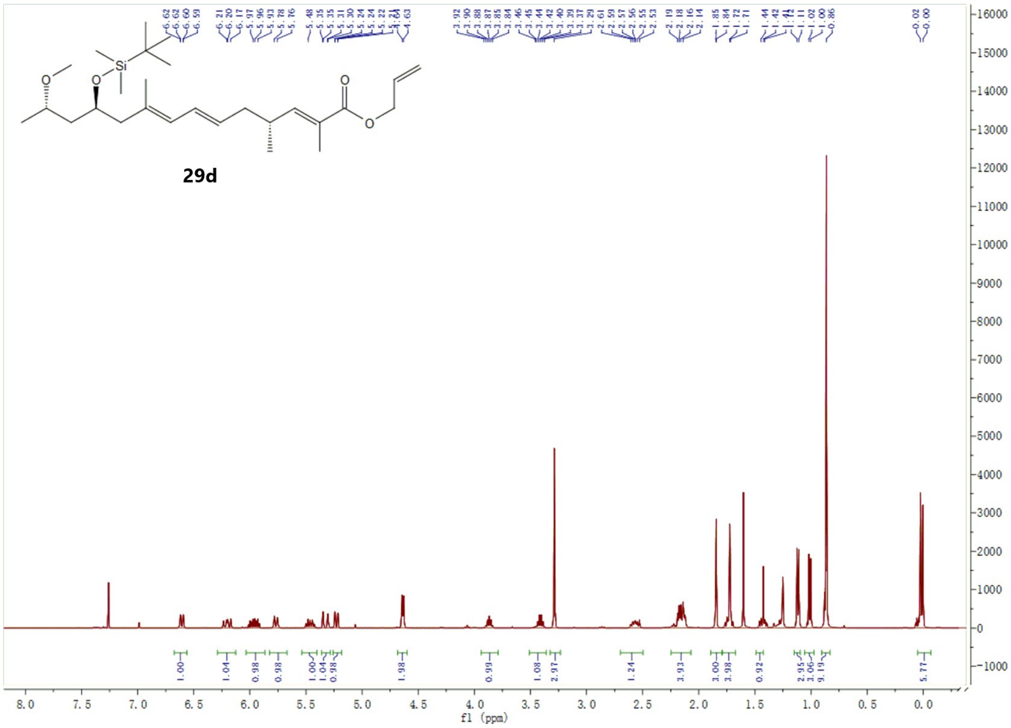

29d
6.62 6.62 6.60 6.59 6.21 6.20 6.17 5.97 5.96 5.93 5.78 5.76 5.48 5.35 5.35 5.31 5.30 5.24 5.24 5.22 5.21 4.64 4.63
3.92 3.90 3.88 3.87 3.85 3.84 3.46 3.45 3.44 3.42 3.40 3.39 3.37 3.29 2.61 2.59 2.57 2.56 2.55 2.53 2.19 2.18 2.16 2.14 1.85 1.84 1.72 1.71 1.44 1.42 1.41 1.12 1.11 1.02 1.00 0.86
0.02 0.00
1.00 1.04 0.98 0.98 1.00 1.04 0.98 1.98 0.99 1.08 2.97 1.24 3.93 3.00 3.98 0.92 2.95 3.06 9.19 5.77
f1 (ppm)

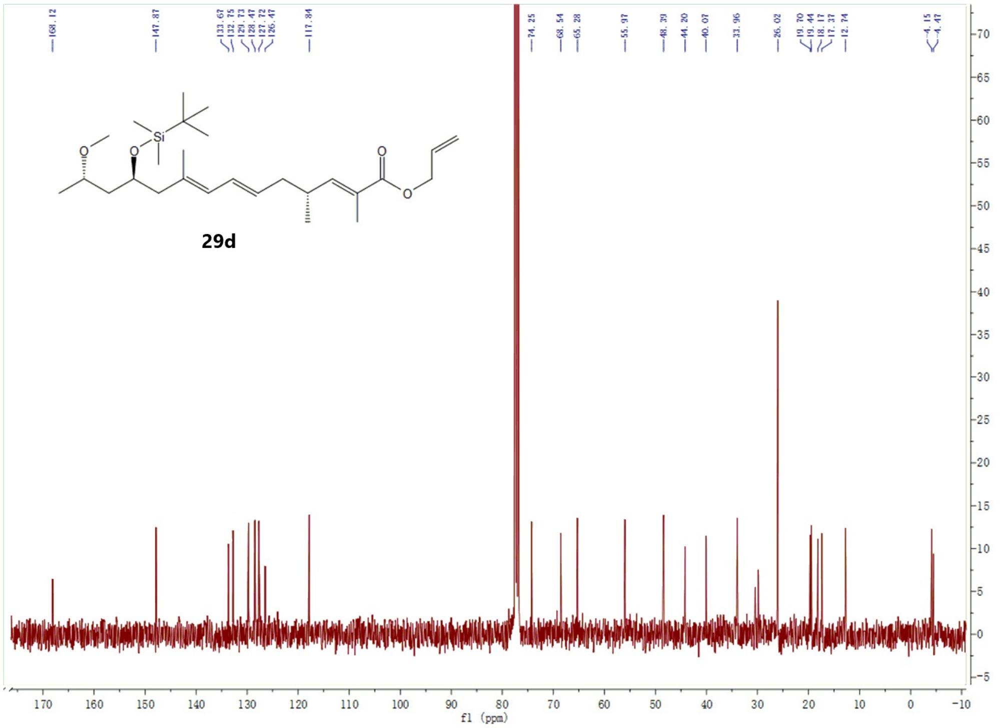

29d
168.12 147.87 133.67 132.75 129.73 128.47 127.72 126.47 117.84
74.25 68.54 65.28 55.97 48.39 44.20 40.07 33.96 26.02 19.70 19.44 18.17 17.37 12.74 -4.15 -4.47
f1 (ppm)

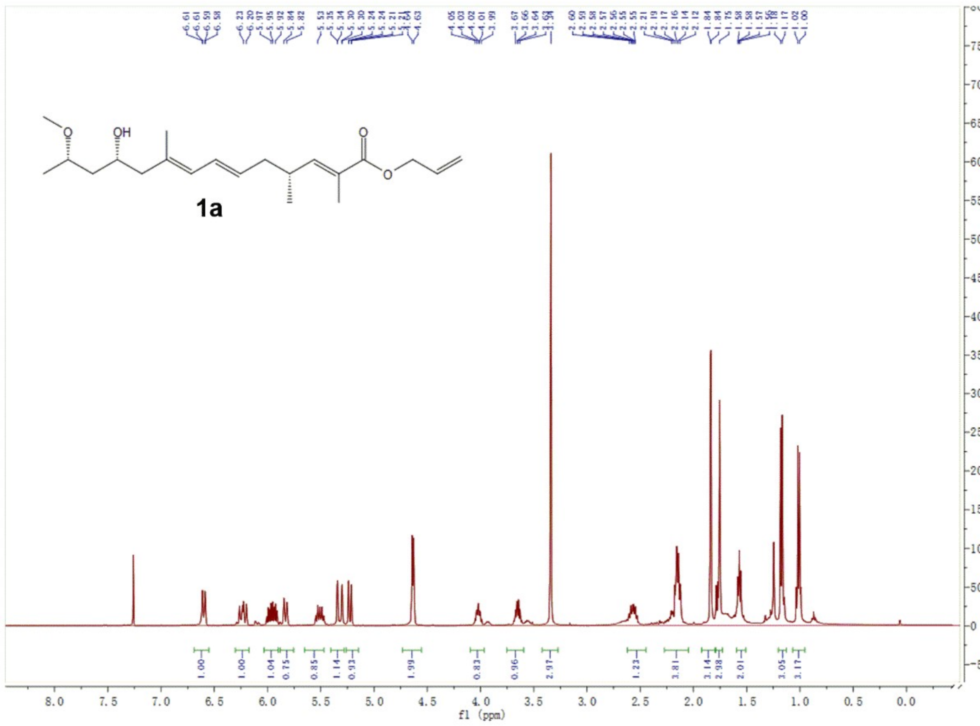
OH
O
1a
f1 (ppm)

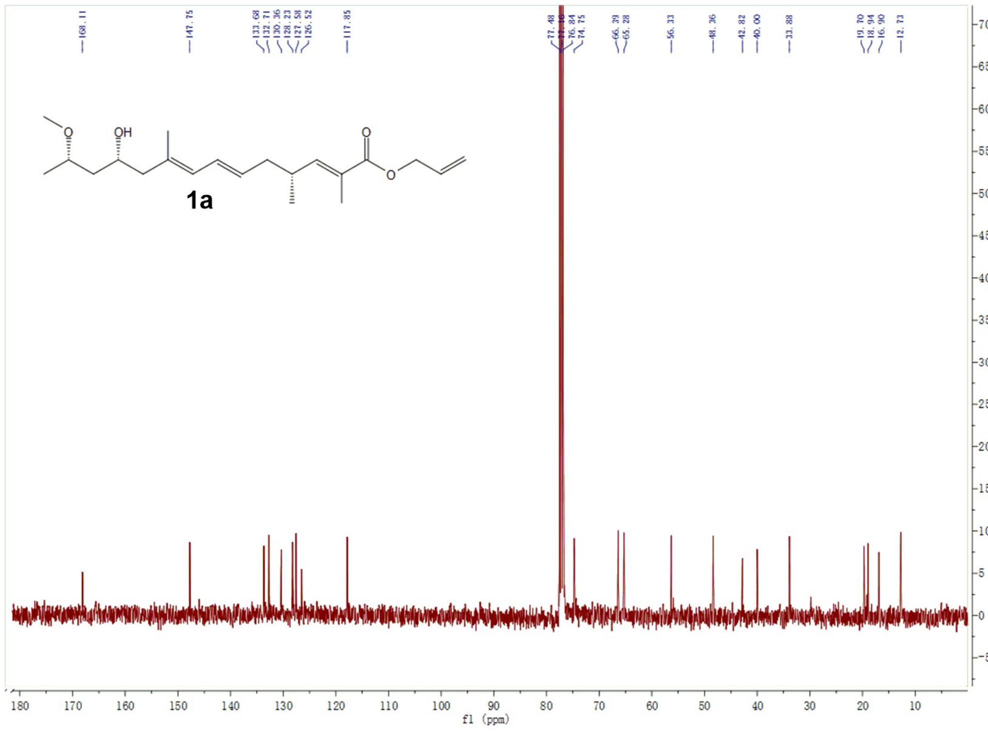
OH
O
1a
f1 (ppm)

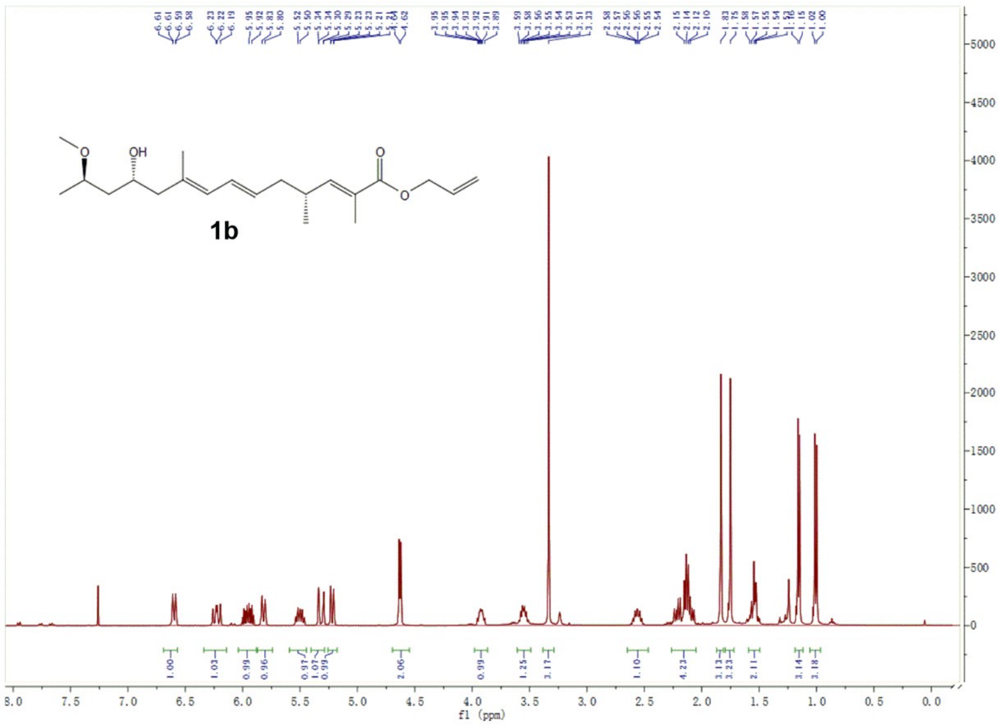
OH
1b
f1 (ppm)

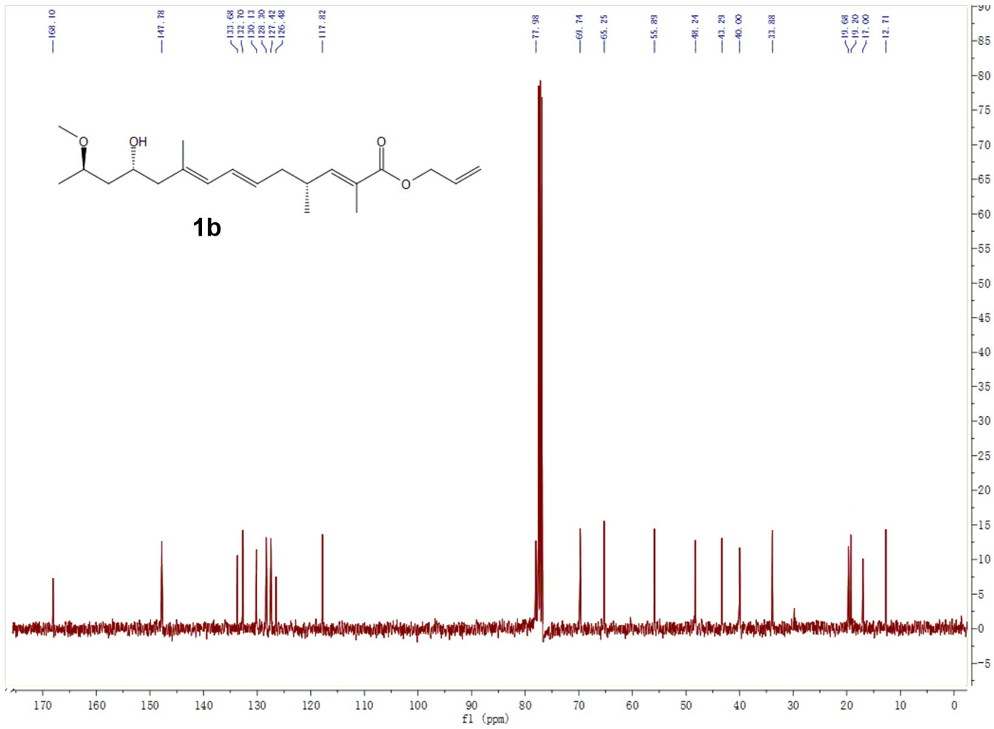
OH
1b
f1 (ppm)

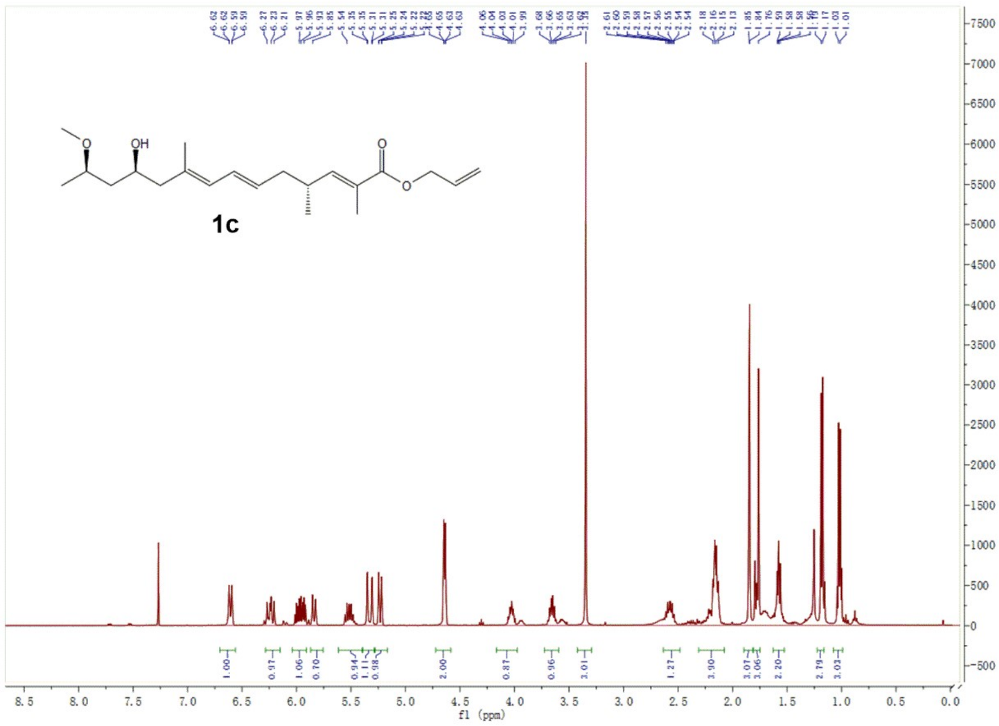
1c
f1 (ppm)

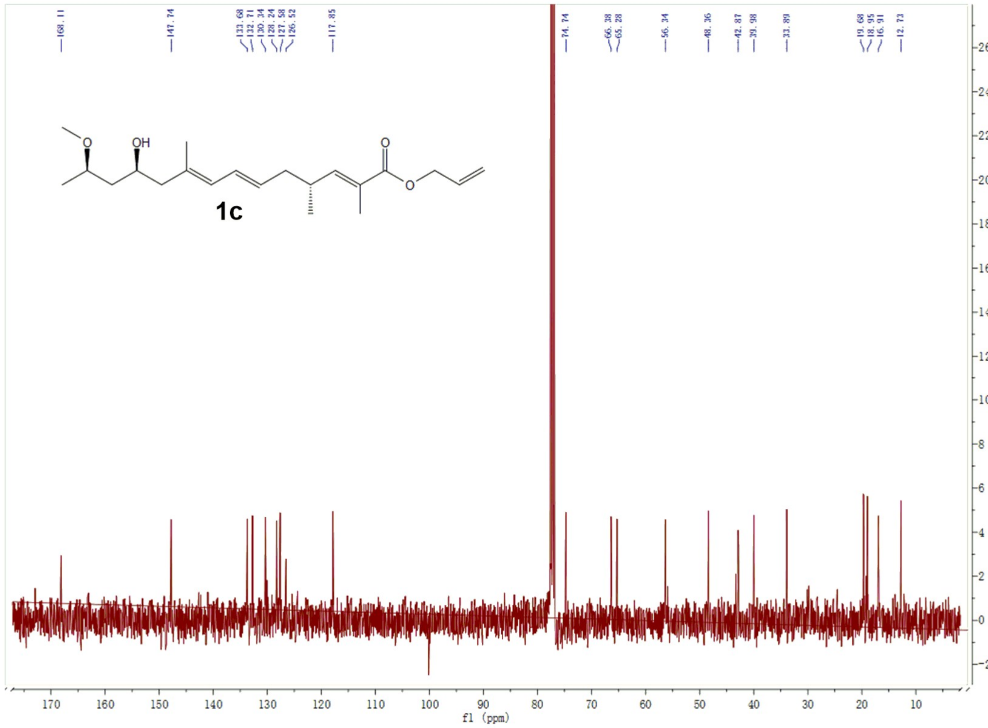
1c
f1 (ppm)

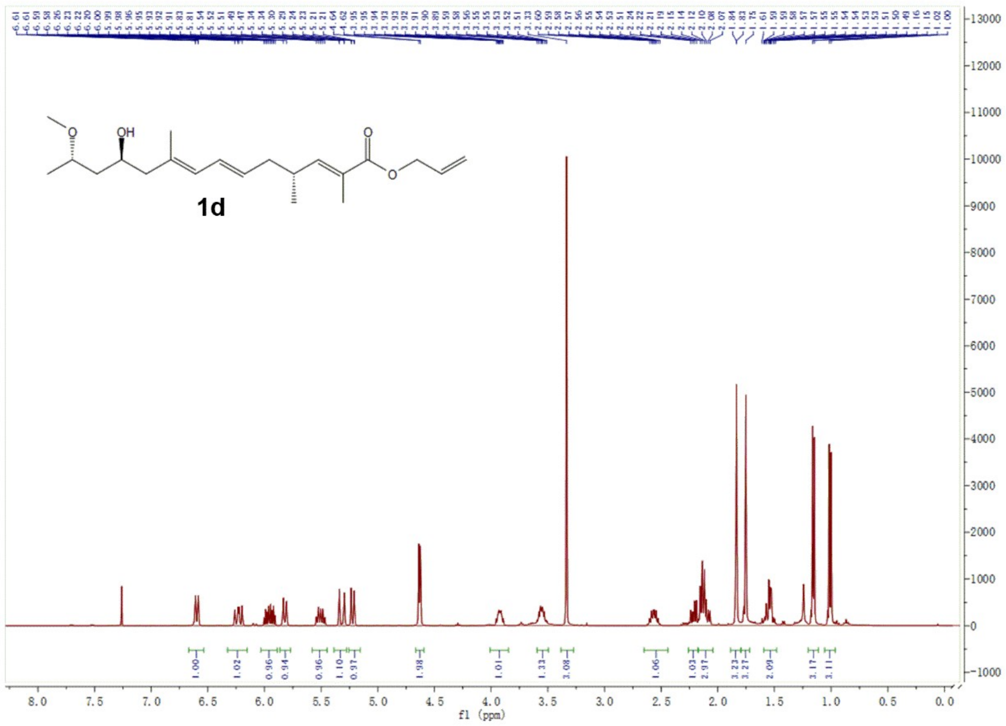
OH
1d
f1 (ppm)

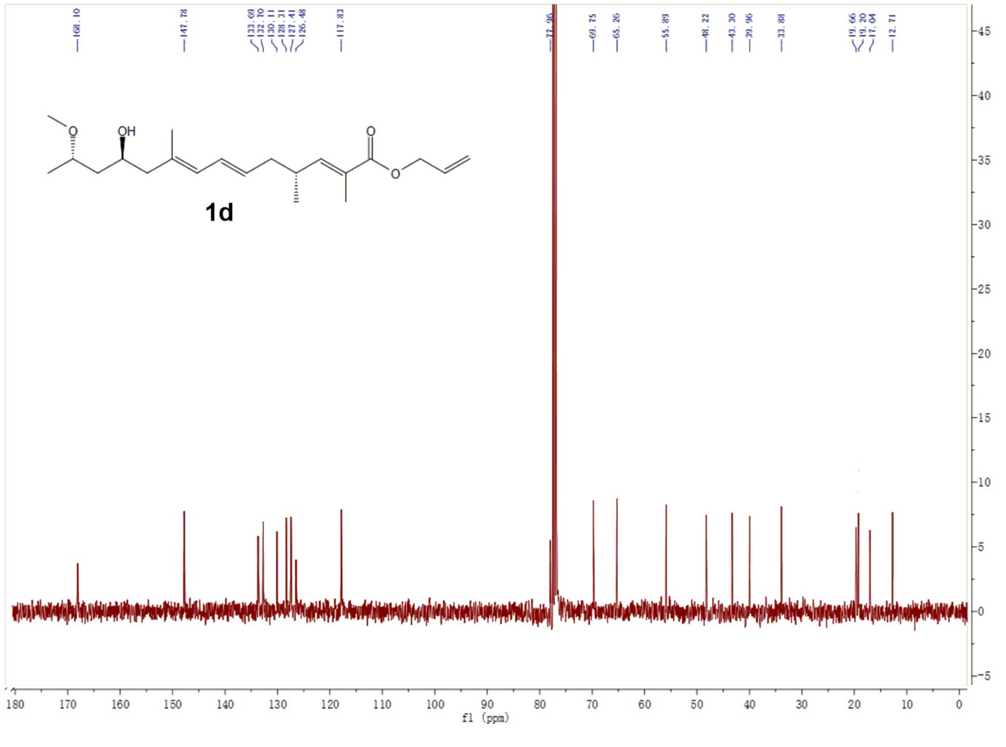
168.10
147.78
133.69
132.70
130.11
128.31
127.41
126.48
117.83
69.75
65.26
55.89
48.22
43.30
39.96
33.88
19.66
19.20
17.04
12.71
OH
1d
f1 (ppm)

NMR data for **1a-1d**

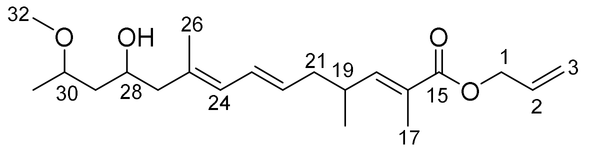

Table 1. NMR data for 1a

Carbon	δC	δH, m, ³J[Hz]
15	168.11	
16	126.52	
17	12.73	1.84 (d, *J* = 1.3 Hz, 3H)
18	147.75	6.60 (dd, *J* = 9.9, 1.3 Hz, 1H)
19	33.89	2.57 (m, 1H)
20	19.70	1.01 (d, *J* = 6.6 Hz, 3H)
21	40.00	2.17 (m, 2H)
22	130.36	5.59 – 5.42 (m, 1H)
23	128.23	6.23 (dd, *J* = 15.0, 10.8 Hz, 1H)
24	127.58	5.83 (d, *J* = 10.8 Hz, 1H)
25	133.68	
26	16.91	1.75 (s, 3H)
27	48.36	2.17 (m, 2H)
28	66.38	4.10 – 3.98 (m, 1H)
29	42.82	1.57 (m, 2H)
30	74.75	3.69-3.61 (m, 1H)
31	18.94	1.17 (d, *J* = 6.2 Hz, 3H)
32	56.34	3.34 (s, 3H)
1	65.28	4.63 (d, *J* = 5.5 Hz, 2H)
2	132.71	5.96 (ddt, *J* = 17.1, 10.5, 5.6 Hz, 1H)
3	117.85	5.32 (dd, *J* = 17.2, 1.5 Hz, 1H)<br>5.22 (dd, *J* = 10.4, 1.2 Hz, 1H)

Table 2. NMR data for 1b

Carbon	δC	δH, m, ³J[Hz]
15	168.10	
16	126.48	
17	12.71	1.83 (d, *J* = 1.3 Hz, 3H)
18	147.78	6.60 (dd, *J* = 9.9, 1.1 Hz, 1H)
19	33.88	2.56 (m, 1H)
20	19.68	1.01 (d, *J* = 6.7 Hz, 3H)
21	40.00	2.14 (m, 2H)
22	130.13	5.50 (dt, *J* = 14.8, 7.3 Hz, 1H)
23	128.30	6.23 (dd, *J* = 15.0, 10.8 Hz, 1H)
24	127.42	5.82 (d, *J* = 10.8 Hz, 1H)
25	133.68	
26	17.00	1.75 (s, 3H)
27	48.24	2.21 (m, 1H), 2.10 (m, 1H)
28	69.74	3.93 (m, 1H)

29	43.29	1.54 (m, 2H)
30	77.98	3.55 (m, 1H)
31	19.20	1.17 (d, J = 6.0 Hz, 3H)
32	55.89	3.33 (s, 3H)
1	65.25	4.63 (d, J = 5.5 Hz, 2H)
2	132.70	5.95 (ddt, J = 17.1, 10.5, 5.6 Hz, 1H)
3	117.82	5.32 (dd, J = 17.2, 1.5 Hz, 1H)<br>5.22 (dd, J = 10.4, 1.2 Hz, 1H)

Table 3. NMR data for 1c

Carbon	δ_C	δ_H, m, ^3J[Hz]
15	168.11	
16	126.52	
17	12.73	1.84 (d, J = 1.3 Hz, 3H)
18	147.74	6.60 (dd, J = 9.9, 1.3 Hz, 1H)
19	33.89	2.57 (m, 1H)
20	19.68	1.01 (d, J = 6.6 Hz, 3H)
21	39.98	2.17 (m, 2H)
22	130.34	5.59 – 5.42 (m, 1H)
23	128.24	6.23 (dd, J = 15.0, 10.8 Hz, 1H)
24	127.58	5.83 (d, J = 10.8 Hz, 1H)
25	133.68	
26	16.91	1.75 (s, 3H)
27	48.36	2.17 (m, 2H)
28	66.38	4.10 – 3.98 (m, 1H)
29	42.87	1.57 (m, 2H)
30	74.74	3.69-3.61 (m, 1H)
31	18.96	1.17 (d, J = 6.2 Hz, 3H)
32	56.34	3.34 (s, 3H)
1	65.28	4.63 (d, J = 5.5 Hz, 2H)
2	132.71	5.96 (ddt, J = 17.1, 10.5, 5.6 Hz, 1H)
3	117.85	5.32 (dd, J = 17.2, 1.5 Hz, 1H)<br>5.22 (dd, J = 10.4, 1.2 Hz, 1H)

Table 4. NMR data for 1d

Carbon	δ_C	δ_H, m, ^3J[Hz]
15	168.10	
16	126.48	
17	12.71	1.83 (d, J = 1.3 Hz, 3H)
18	147.78	6.60 (dd, J = 9.9, 1.1 Hz, 1H)
19	33.88	2.56 (m, 1H)
20	19.66	1.01 (d, J = 6.7 Hz, 3H)
21	39.96	2.14 (m, 2H)
22	130.11	5.50 (dt, J = 14.8, 7.3 Hz, 1H)
23	128.31	6.23 (dd, J = 15.0, 10.8 Hz, 1H)
24	127.41	5.82 (d, J = 10.8 Hz, 1H)
25	133.69	
26	17.04	1.75 (s, 3H)
27	48.22	2.21 (m, 1H), 2.10 (m, 1H)

28	69.75	3.93 (m, 1H)
29	43.30	1.54 (m, 2H)
30	77.96	3.55 (m, 1H)
31	19.20	1.17 (d, *J* = 6.0 Hz, 3H)
32	55.89	3.33 (s, 3H)
1	65.26	4.63 (d, *J* = 5.5 Hz, 2H)
2	132.70	5.95 (ddt, *J* = 17.1, 10.5, 5.6 Hz, 1H)
3	117.83	5.32 (dd, *J* = 17.2, 1.5 Hz, 1H)<br>5.22 (dd, *J* = 10.4, 1.2 Hz, 1H)
